# *N* ^α^-arylsulfonyl histamines as selective β-glucosidase inhibitors†

**DOI:** 10.1039/c8ra06625f

**Published:** 2018-10-24

**Authors:** M. O. Salazar, M. I. Osella, I. A. Ramallo, R. L. E. Furlan

**Affiliations:** Farmacognosia, Departamento de Química Orgánica, Facultad de Ciencias Bioquímicas y Farmacéuticas, Universidad Nacional de Rosario Suipacha 531 Rosario S2002LRK Argentina rfurlan@bioyf.unr.edu.ar

## Abstract

*N* ^α^-benzenesulfonylhistamine, a new semi-synthetic β-glucosidase inhibitor, was obtained by bioactivity-guided isolation from a chemically engineered extract of *Urtica urens* L. prepared by reaction with benzenesulfonyl chloride. In order to identify better β-glucosidase inhibitors, a new series of *N*^α^,*N*^τ^-di-arylsulfonyl and *N*^α^-arylsulfonyl histamine derivatives was prepared. Biological studies revealed that the β-glucosidase inhibition was in a micromolar range for several *N*^α^-arylsulfonyl histamine compounds of the series, *N*^α^-4-fluorobenzenesulfonyl histamine being the most powerful compound. Besides, this reversible and competitive inhibitor presented a good selectivity for β-glucosidase with respect to other target enzymes including α-glucosidase.

## Abstract 2

A selective β-glucosidase inhibitor was discovered using the chemically engineered extracts approach.

## Graphical abstract

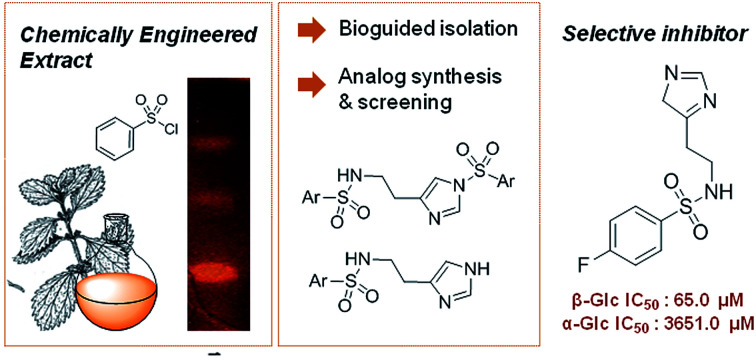

## Introduction

Chemically engineered extracts (CEEs) are mixtures of semi-synthetic compounds produced through the chemical transformation of natural extracts, and represent a still scarcely explored source of bioactive molecules.^1–17^ Depending on the reagents applied, CEEs can contain compounds with elements, such as sulfur and fluorine, which are not very common in natural products (NPs) but are relevant for bioactivity. Organosulfur compounds have an extraordinary medicinal impact; approximately 25% of Top200 drugs prescriptions in EEUU contain this heteroatom.^18^ Sulfonamides are some of the most common sulfur containing drugs.^19^ Although some of these drugs have a natural origin, the average proportion of sulfur in natural products is significantly lower than in drugs.^20^

Natural organofluorine compounds are very rare;^21^ they represent less than 1% of the naturally occurring organohalogens.^22^ Incorporation of fluorine into a molecule can modulate its physicochemical properties and metabolic stability.^23^ The strategic use of fluorine substitution in drug design has led to the production of some of the key drugs available on the market.^24,25^ Consequently, it is not surprising that the average proportion of fluorine in drugs is significantly higher than in NPs,^26^ and that 20–25% of drugs in the pharmaceutical pipeline contain at least one fluorine atom.^23^

We have prepared CEEs enriched in sulfur containing molecules^3^ or in fluorinated molecules.^13^ When certain aryl sulfonyl chlorides were used for herbal extract derivatization, an increase in the inhibition properties towards the enzyme β-glucosidase (β-Glc) was observed.^5^ Bioguided fractionation of the CEE of *Urtica urens* L. (Urticaceae) modified with benzenesulfonyl chloride led to identification of the *N*^α^,*N*^τ^-di-benzenesulfonyl histamine (II-a) as one of the compounds responsible for the observed β-Glc inhibition.^5^ This enzyme catalyzes the hydrolysis of β-bonds in polysaccharides and oligosaccharides, and the breakdown of β-bonds between sugars and aglycones. Compounds that inhibit glucosidases are of great interest^27^ for their potential as drugs in the treatment of diabetes,^28,29^ viral infections,^30,31^ obesity,^32^ hereditary lysosomal diseases,^33,34^ and tumors in general.^35^

With the purpose to explore the value of CEEs as a source of unexpected glucosidase inhibitors, in this work we analyze further the composition and bioactivity of the *U. urens* CEE and identify a new semi-synthetic β-Glc inhibitor that inspired the preparation of a small library of fluorinated arylsulfonyl analogues with improved inhibition properties.

## Results and discussion

### Identification and biological characterization of the new inhibitor from the CEE of *U. urens*

**Fig. 1:**
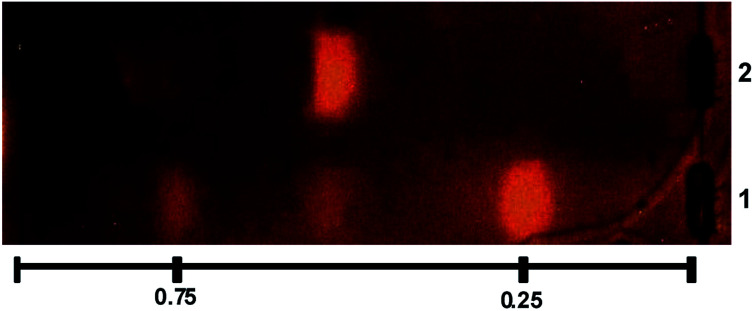
TLC of UU-CEE (line 1) and UU-NE (line 2) revealed for β-Glc activity. The TLC plate was developed in DCM : MeOH (9 : 1).

The β-Glc inhibitory properties of the *U. urens* L. chemically engineered extract (UU-CEE) and the precursor native extract (UU-NE) were analyzed by thin layer chromatography (TLC) coupled to bioautography.^36^ Two inhibition spots were detected in the UU-CEE and were absent in the UU-NE (Fig. 1). Possibly, these inhibition spots were produced by products of the chemical transformation of some natural components of the native extract. The spot with higher *R*_f_ value was produced by *N*^α^,*N*^τ^-di-arylsulfonylated histamine, previously isolated from the UU-CEE (Fig. 1, line 1, *R*_f_ = 0.75) II-a.^5^ In order to identify the product responsible of the spot observed at *R*_f_ = 0.25 (Fig. 1, line 1), the UU-CEE was subjected to bioguided fractionation using chromatography on different supports, and TLC-bioautography.

**Scheme 1:**
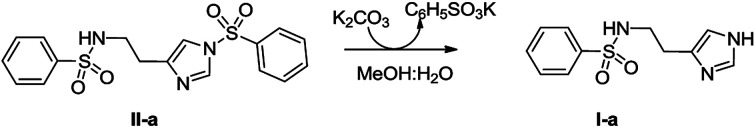
Synthesis of *N*^α^-benzenesulfonyl histamine (I-a) from *N*^α^,*N*^τ^-di-benzenesulfonyl histamine (II-a).

High resolution mass spectrometry (HRMS) analysis of the purified bioactive fraction of UU-CEE evidenced a [M + H]^+^ signal at *m*/*z* = 252.0810 corresponding to a molecular formula C_11_H_14_N_3_O_2_S. MS-MS analysis showed a characteristic fragment generated by a benzenesulfonyl radical (*m*/*z* = 141) suggesting that the active compound could be *N*^α^-benzenesulfonyl histamine (I-a). To confirm the proposed structure, I-a was prepared from II-a in MeOH : H_2_O : K_2_CO_3_ (Scheme 1).^37^ Under these conditions the *N*^τ^ was selectively deprotected to give a main product whose NMR spectra, HRMS isotope pattern and MS-MS profiles, corroborated the structure of I-a. In addition, the β-Glc TLC-bioautography profiles of the UU-CEE purified bioactive fraction, and the synthetic product I-a were coincident.

The inhibitory potency of I-a was determined using a microplate assay based on the *p*-nitrophenyl β-d-glucopiranoside hydrolysis.^38^ The IC_50_ value for I-a was 197.2 μM, in the same order of magnitude of the value reported for II-a (IC_50_ = 250 μM).^5^ Under these experimental conditions, histamine was inactive (IC_50_ > 1000 μM).

It is known that imidazole binds the active site of β-Glc from almonds (*K*_i_ 0.53 mM). Histamine is a much weaker inhibitor (*K*_i_ 2.1 mM);^39^ however, the presence of a benzyl substituent on its amino group increases the inhibitory capacity, suggesting that the aromatic ring could be interacting with a hydrophobic pocket at the active site of the enzyme.^27^ Furthermore, Li *et al.* found that a benzene ring located at four or five links away from the imidazole ring is the most effective distance,^40^ in agreement with the distance observed in I-a and II-a.

### Synthesis and biological screening of a library based in arylsulfonyl histamine moiety

In order to explore the potential of histamine derived arylsulfonylamides, a series of mono and disubstituted derivatives were prepared by reaction of histamine with different arylsulfonyl chlorides (Table 1). Since the CEEs approach points to complement nature biosynthetic machinery by incorporating elements that are rarely found in secondary metabolites, we selected fluorinated reagents.

**Table 1:** Compounds type I (I-b to I-j) and type II (II-b to II-j)

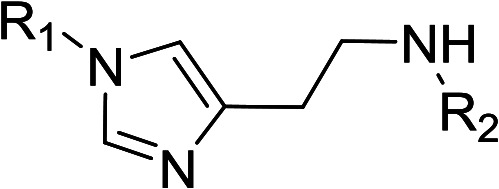	
Type I	Type II
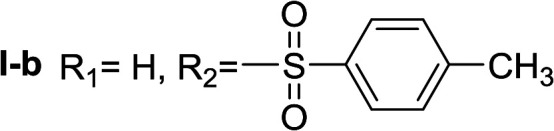	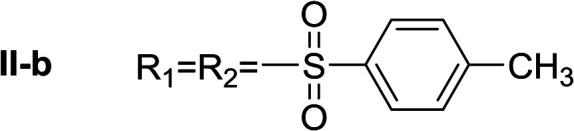
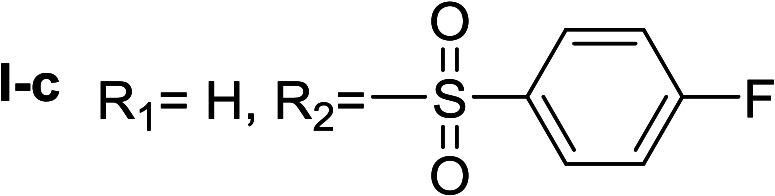	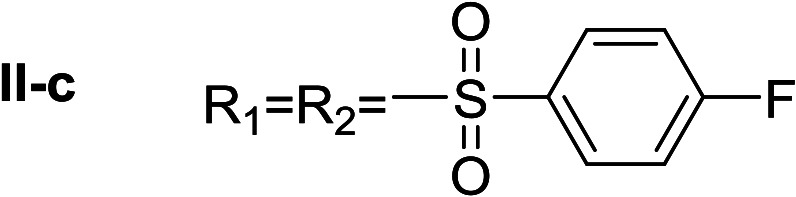
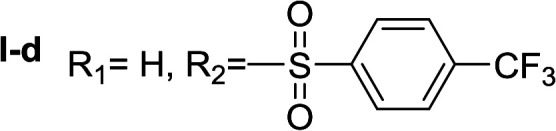	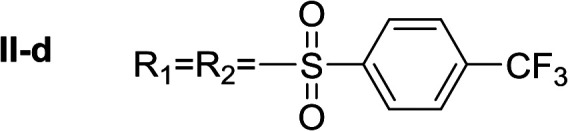
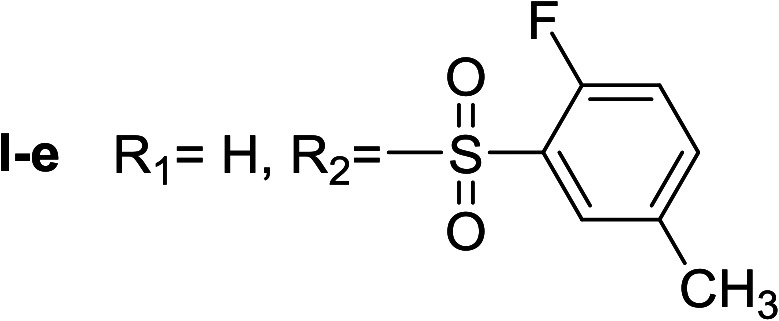	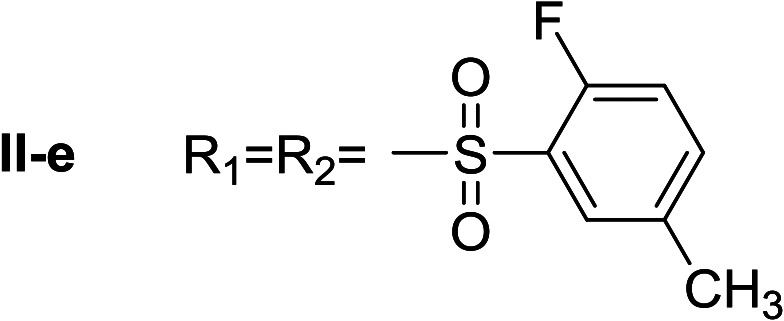
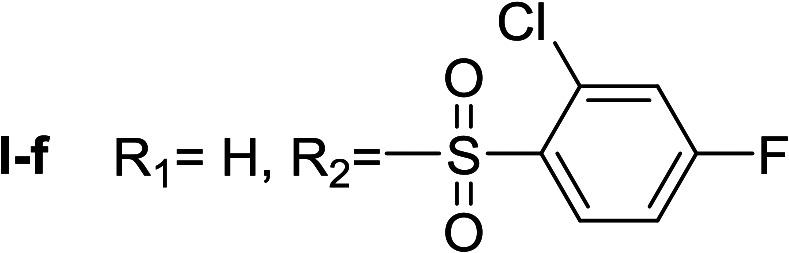	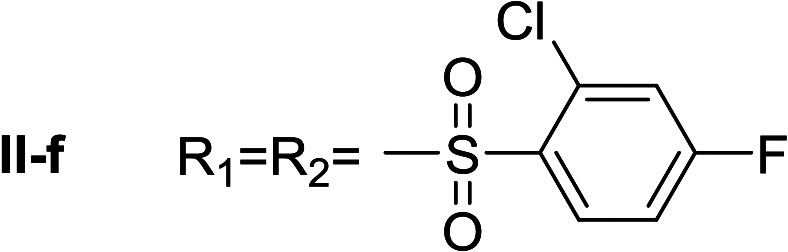
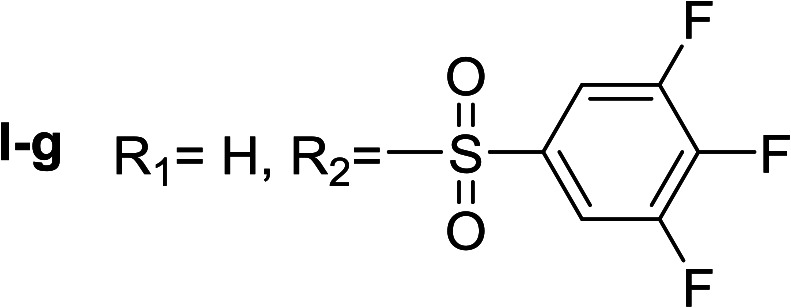	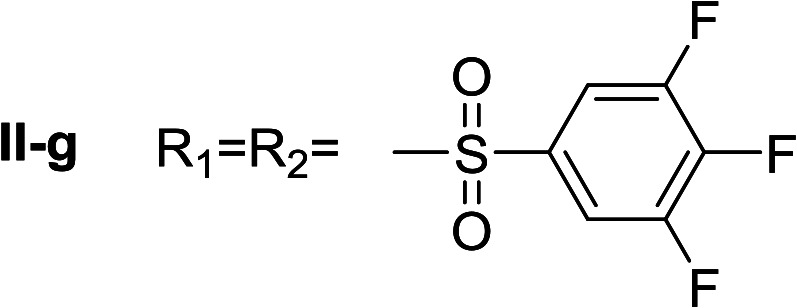
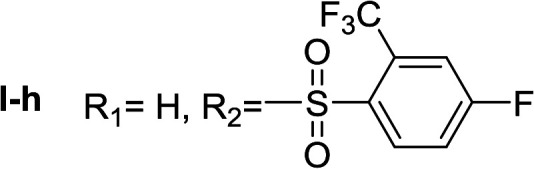	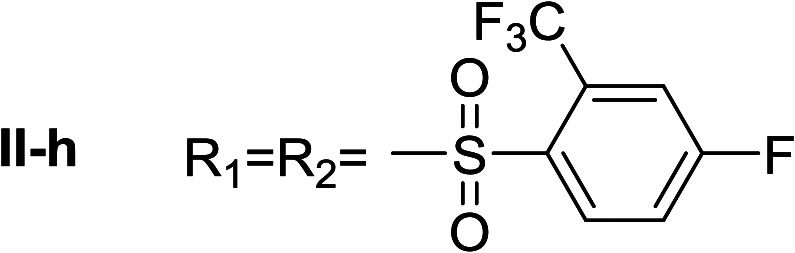
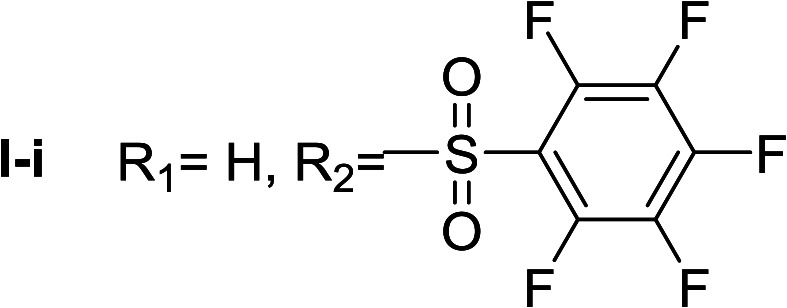	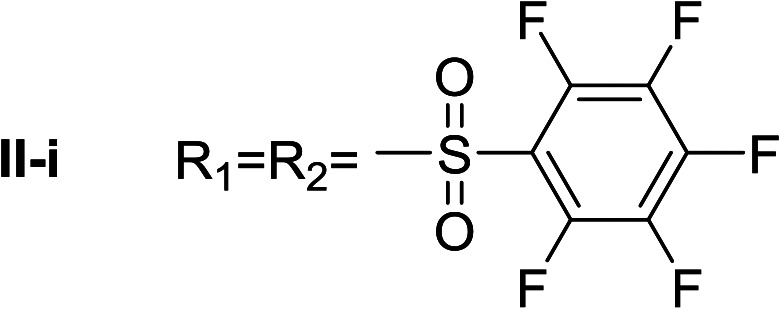
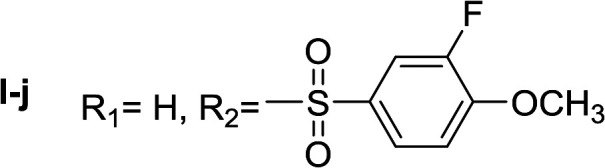	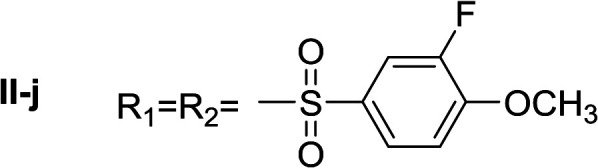

In all cases, the *N*^α^,*N*^τ^-di-arylsulfonylated derivatives (type II compounds, Table 1) were obtained as the main product (45–81% yield). The *N*^α^-arylsulfonyl histamine derivatives (type I compounds, I-b to I-f, Table 1) were not detected in the reaction mixtures, or detected as minor products. However, these derivatives could be obtained by deprotection of the type II compounds.

**Fig. 2:**
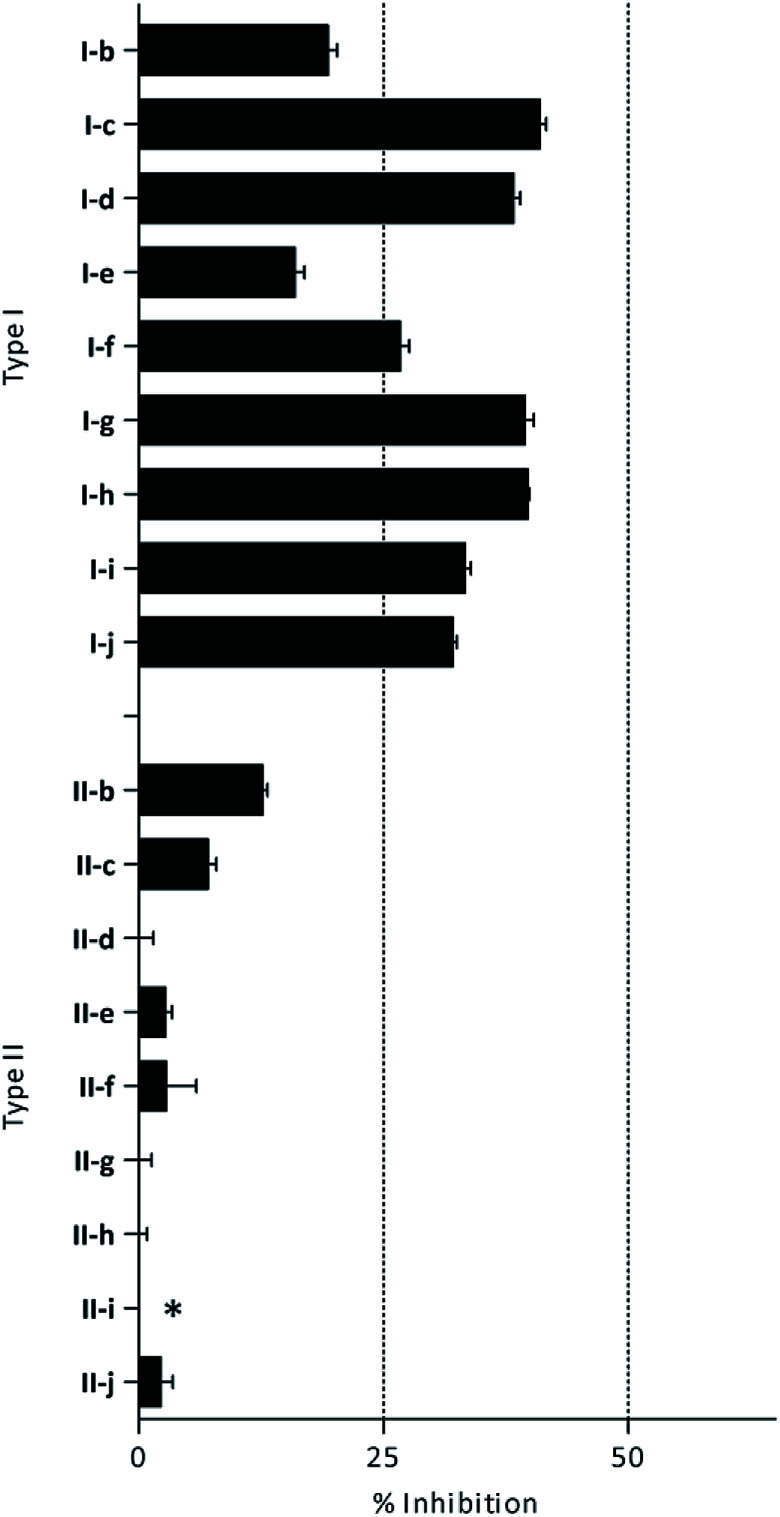
β-Glc inhibition by histamine derivatives (type I and II): % of inhibition at 40 μM. *Turbidity was observed at the tested concentration.

β-Glc inhibition was initially quantified at a fixed compound concentration (40 μM) in a microplate assay. This concentration is approximately half of the reported IC_50_ value for the reference β-Glc inhibitor 1-deoxynojirimycin (1-DNJ, IC_50_ = 81 μM).^41^ All type I compounds showed inhibition higher than 25%, with the exception of compound I-e (Fig. 2). On the contrary, most type II compounds did not produce significant enzyme inhibition.

According to these preliminary results, the presence of an arylsulfonyl substituent on the imidazole ring decreases the inhibitory properties. This could be related to the absence of a proton in *R*_1_ position available to participate in key hydrogen bonding in the active site of the enzyme.^40^

Given the interesting inhibition observed for most compounds of the type I, the series was tested at different concentrations to determine the IC_50_. Although the values ranged between 65–420 μM (Table 2), compounds I-c, I-d, I-g and I-h showed very interesting inhibitory activities, similar to 1-DNJ.

**Table 2:** IC_50_ Values (μM) for *N*^α^-arylsulfonyl histamine derivatives. 1-DNJ:reference inhibitor

Type I compounds	IC_50_ (μM)
I-b	250.00 ± 1.03
I-c	65.08 ± 1.04
I-d	79.50 ± 1.05
I-e	421.6 ± 0.94
I-f	244.2 ± 1.09
I-g	71.43 ± 1.02
I-h	72.69 ± 1.05
I-i	102.20 ± 1.05
I-j	95.55 ± 1.02
1-DNJ	65.18 ± 1.04

Apparently, the observed inhibition is related to the substitution motif present at the aromatic moiety. A *para*-fluor substituted compound (I-c) showed the best inhibitory effect. When a fluorine atom replaces the hydrogen atom in that position, the inhibitory effect improves substantially (I-c respect to I-a). A similar increase is observed when the CH_3_ group (I-b) is replaced with CF_3_ (I-d). Variations of the aromatic substitution pattern did not improve the inhibitory capacity of the compounds compared to I-c. An increase in the number of fluorine substituents generated derivatives with similar (I-g and I-h) or lower inhibitory potency (I-i) than I-c. A decrease in the inhibition capacity was observed when the fluorine atom was placed in *meta* position (I-j), and this effect is still more pronounced when the substituent is in *ortho* position (I-e). In addition, as observed for compound I-f when a chlorine atom is added in position 2 the inhibitory potency decreases.

**Fig. 3:**
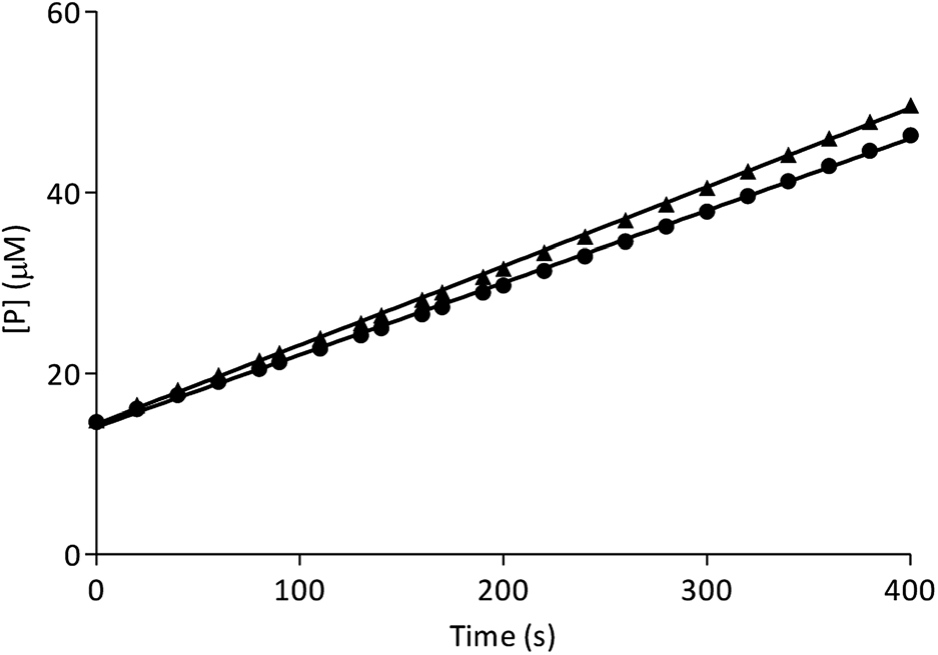
Jump dilution progress curve for control (triangles) and I-c (circles).

In order to characterize the type of β-Glc inhibition, a jump dilution assay was carried out with the best inhibitor I-c.^42^ Enzyme activity recovery was 91%, which indicates a reversible enzyme–inhibitor interaction (Fig. 3).

**Fig. 4:**
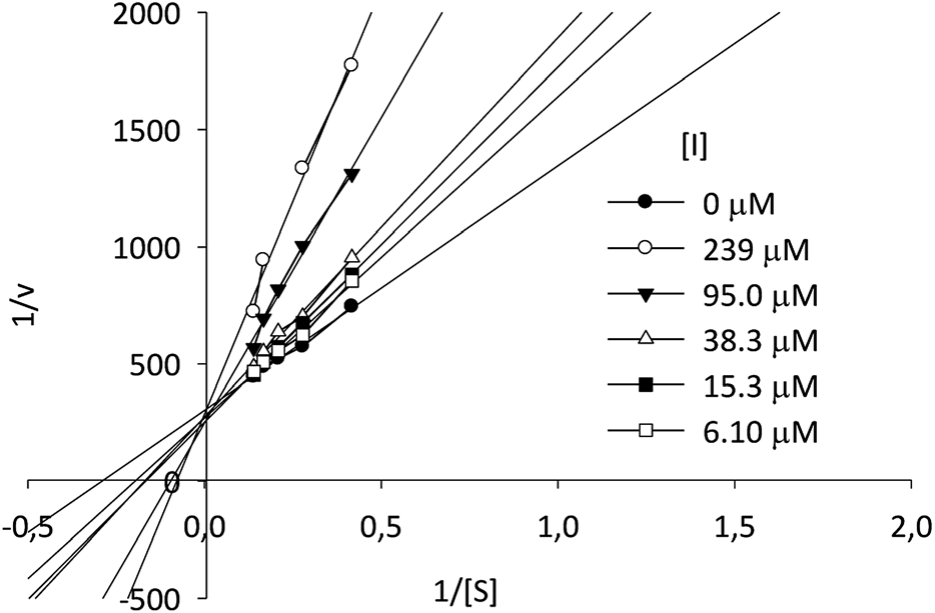
Lineweaver–Burk plot of β-Glc inhibition at different concentrations of *p*-NPG and compound I-c.

Kinetic assays showed a *K*_i_ value of 8.11 μM, almost six times lower than the *K*_i_ value for the reference compound 1-DNJ (*K*_i_ = 47 μM), measured in similar conditions.^43^ According to the Lineweaver–Burk plot, compound I-c displays a typical behavior for a competitive type inhibition (Fig. 4).

**Fig. 5:**
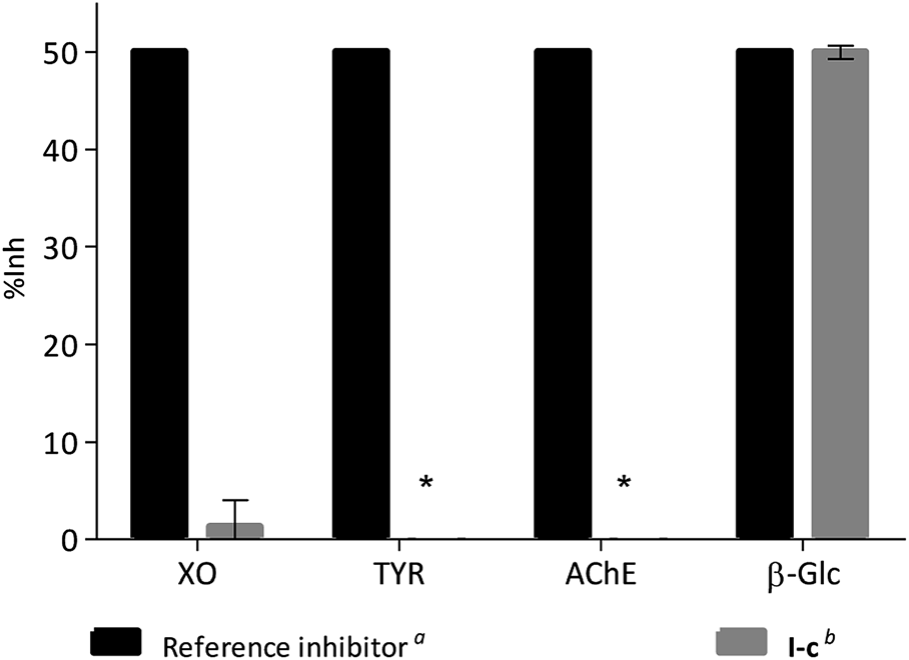
Comparison of the inhibitory potency of compound I-c*vs.* the reference inhibitor for each enzyme tested. ^*a*^Allopurinol for XO (IC_50_ 2.52 μM), kojic acid for TYR (IC_50_ 40.00 μM), eserine for AChE (IC_50_ 1.17 μM) and 1-DNJ for β-Glc (IC_50_ 65.18 μM). ^*b*^Compound I-c was tested at 3 μM in XO assay, at 40 μM in TYR assay, 1.5 μM in AChE assay and 65 μM in β-Glc. *No inhibition was observed for TYR or AChE.

In order to evaluate if this kind of compounds are promiscuous enzyme inhibitors, the activity of compound I-c was measured using two oxidases, xanthine oxidase (XO) and tyrosinase (TYR), and another hydrolase, acetyl cholinesterase (AChE). In each case compound I-c was tested at a concentration similar to the IC_50_ value of a reference inhibitor for the enzyme (Fig. 5). Whilst 49.94% inhibition was observed for β-Glc, no inhibition was observed for TYR or AChE, and only 1.31% inhibition was observed for XO. Similar results were observed for compounds I-d, I-g, I-h and I-j (Fig. S1†). Moreover, when I-c was tested against α-glucosidase (α-Glc) weak inhibition was observed (IC_50_ = 3651 ± 1.08 μM), whereas no inhibition was observed against β-galactosidase (β-Gal, up to 3.500 mM). Similarly, compounds I-d, I-g, I-h and I-j all are better β-Glc inhibitors than α-Glc and β-Gal (Fig. S2†).

## Experimental

Chemical reagents and enzymes were purchased at commercial sources and were used without further purification unless noted otherwise. Solvents were analytical grade or were purified by standard procedures prior to use.

^1^H NMR spectra were recorded on a Bruker advance II at 300 MHz in CDCl_3_, CD_3_OD or acetone-*d*_6_, in the presence of TMS (0.00 ppm) as the internal standard. ^13^C NMR spectra were recorded on the same apparatus at 75 MHz with CDCl_3_, CD_3_OD or acetone-*d*_6_, in the presence of TMS (0.00 ppm) as the internal standard; ^13^C NMR assignments were made on the basis of chemical shifts and proton multiplicities (COSY ^1^H–^1^H, HSQC and HMBC).

High Resolution Mass spectra were recorded on a Bruker micrOTOF-Q II spectrometer (Bruker-Daltonics) or Xevo G2S QTOF mass spectrometer (Waters Corporation, Manchester, UK) with an electrospray ionization (ESI) source. Acetonitrile (acquired from Carlo Erba) was used for samples preparation. MS and MS-MS Parameters: source type, ESI; ion polarity, positive; set nebuliser, 0.4 Bar; set dry heater, 180 °C; set dry gas, 4.0 L min^−1^; set capillary, 4500 V; set end plate offset, 500 V; set collision cell radio frequency, 150.0 V_pp_. ISC collision energy for MS-MS experiments: 30 eV.

### β-Glc autography assay

Staining solution for β-Glc. Agar (52.5 mg) was dissolved at 80 °C in sodium acetate buffer (0.1 M, pH 5, 7.5 mL). The solution was allowed to cool to 60 °C and ferric chloride solution (0.5% w/v in 0.1 M acetate buffer, 0.9 mL) was added and the whole mixed by inversion. At 40 °C, β-Glc solution in 0.1 M acetate buffer (2.5 U mL^−1^, 25 μL) was added and the obtained solution mixed by inversion.

Typical procedure for the detection of β-Glc inhibition. An aliquot of approximately 6.5 mL of β-Glc staining solution was distributed over the TLC layer (10 × 5 cm). After the staining solution had solidified, the TLC plate was incubated at 37 °C for 120 min and immersed in 0.2% w/v solution of esculin in 0.1 M acetate buffer, and again incubated at 37 °C for 120 min. Clear spots (representing areas exhibiting inhibition of β-Glc activity) were observed against a dark brown background.

### Bioguided fractionation de UU-CEE

5.52 g of UU-CEE was chromatographed on silica gel (hexane : ethyl acetate gradient, from 99 : 1 to pure ethyl acetate). The active fraction *F*_1_ (541.4 mg) was chromatographed on Sephadex® (chloroform : hexane : MeOH, 10 : 10 : 20) followed by preparative TLC to obtain 10.4 mg of F1a. The high resolution mass spectrum of F1a showed a main signal with *m*/*z* = 252.08096, calculated *m*/*z* for C_11_H_14_N_3_O_2_S [M + H]^+^ 252.08012 (2.4 ppm error). The observed isotope pattern was *m*/*z* 252.08096 (abundance 100%), 253.08245 (abundance 14.00%), 254.07730 (abundance 5.34%), 255.07951 (abundance 0.69%). The calculated isotope pattern is *m*/*z* 252.08012 (abundance 100%), 253.082792 (abundance 14.04%), 254.07800 (abundance 5.85%), 255.07996 (abundance 0.69%). MS-MS showed the fragment ion corresponding to benzenesulfonyl radical at *m*/*z* = 141.

### *N* ^α^,*N*^τ^-di-arylsulfonyl histamine analogues synthesis

#### General procedure

A solution of histamine (0.5 mmol), arylsulfonyl chloride (1.0 mmol) and K_2_CO_3_ (1.4 mmol) in acetone (10 mL) was stirred under reflux during 24 h. The solvent was eliminated under reduced pressure, and the resulting residue was chromatographed on silica gel (hexane : AcOEt gradient) to obtain type II compounds.

##### *N* ^α^,*N*^τ^-di-benzenesulfonyl histamine (II-a)

The general procedure was followed employing 1.0 eq. of benzenesulfonyl chloride to afford 85.8 mg of II-a (45% final yield). ^1^H RMN (300 MHz, CDCl_3_) *δ* = 7.89 (1H, s, N=CH–N), 7.00 (1H, s, N–CH=C), 2.65 (2H, t, *J*_1_ = *J*_2_ = 6.25 Hz, CH_2_–CH_2_-imidazole), 3.23 (2H, q, *J*_1_ = *J*_2_ = 6.2 Hz y *J*_3_ = 6.2 Hz, CH_2_-imidazole), 5.56 (1H, t, *J*_1_ = *J*_2_ = 5.8 Hz, NH), 7.93 (2H, d, *J* = 7.9 Hz, Ar), 7.70 (1H, t, *J*_1_ = *J*_2_ = 7.3 Hz, Ar), 7.53–7.61 (3H, m, Ar), 7.82 (2H, d, *J* = 7.6 Hz, Ar), 7.47 (2H, t, *J*_1_ = *J*_2_ = 7.45 Hz, Ar). ^13^C RMN (75 MHz, CDCl_3_) *δ* = 136.40 (CH, N=CH–N), 114.12 (CH, N–CH=C), 141.90 (C, N–CH=CN), 27.49 (CH_2_, CH_2_-imidazole), 42.21 (CH_2_, CH_2_–CH_2_-imidazole), 137.78 (C, Ar), 140.06 (C, Ar), 127.33 (2 CH, Ar), 126.95 (2 CH, Ar), 129.91 (2 CH, Ar), 129.07 (2CH, Ar), 134.97 (CH, Ar), 132.56 (CH, Ar). HRMS: found *m*/*z* = 392.07282, calculated *m*/*z* for C_17_H_18_N_3_O_4_S_2_ [M + H]^+^ 392.07332 (0.5 mDa error). MS-MS showed the fragment ion corresponding to benzenesulfonyl histamine at *m*/*z* = 252 and to the benzenesulfonyl radical at *m*/*z* = 141.

##### *N* ^α^,*N*^τ^-di-*p*-toluenesulfonyl histamine (II-b)

The general procedure was followed employing 1.0 eq. of *p*-toluenesulfonyl chloride to afford 101.3 mg of II-a (48% final yield).

^1^H RMN (300 MHz, CDCl_3_) *δ* = 7.87 (1H, s, N=CH–N), 6.98 (1H, s, N–CH=C), 2.64 (2H, t, *J*_1_ = *J*_2_ = 6.2 Hz, CH_2_–CH_2_-imidazole), 3.20 (2H, q, *J*_1_ = *J*_2_ = 6.2 Hz y *J*_3_ = 6.2 Hz, CH_2_-imidazole), 5.48 (1H, t, *J*_1_ = *J*_2_ = 5.8 Hz, NH), 2.41 (3H, s,CH_3_–Ar), 2.44 (3H, s,CH_3_–Ar), 7.80 (2H, d, *J* = 8.10 Hz, Ar), 7.69 (2H, d, *J* = 8.1 Hz, Ar), 7.36 (2H, d, *J* = 8.1 Hz, Ar), 7.26 (2H, d, *J* = 8.1 Hz, Ar). ^13^C RMN (75 MHz, CDCl_3_) *δ* = 136.31 (CH, N=CH–N), 114.05 (CH, N–CH=C), 141.78 (C, N–CH=CN), 27.48 (CH_2_, CH_2_–CH_2_-imidazole), 42.19 (CH_2_, CH_2_-imidazole), 21.50 (CH_3_, Ar-CH_3_), 21.74 (CH_3_, Ar–CH_3_), 137.05 (C, Ar), 143.32 (C, Ar), 127.01 (2 CH, Ar), 129.67 (2 CH, Ar), 127.39 (2 CH, Ar), 130.50 (2 CH, Ar), 134.76 (C, Ar), 146.43 (C, Ar). HRMS: found *m*/*z* = 420.1048, calculated *m*/*z* for C_19_H_22_N_3_O_4_S_2_ [M + H]^+^ 420.1046 (0.2 mDa error). MS-MS showed the fragment ion corresponding to *p*-toluensulfonyl histamine at *m*/*z* = 266 and to the *p*-toluenesulfonyl radical at *m*/*z* = 155.

##### *N* ^α^,*N*^τ^-di-4-fluorobenzenesulfonyl histamine (II-c)

The general procedure was followed employing 1.0 eq. of 4-fluorobenzenesulfonyl chloride to afford 116.3 mg of II-c (55% final yield). ^1^H RMN (300 MHz, CDCl_3_) *δ* = 7.89 (1H, s, N=CH–N), 7.03 (1H, s, N–CH=C), 2.67 (2H, t, *J*_1_ = *J*_2_ = 6.2 Hz, CH_2_–CH_2_-imidazole), 3.23 (2H, q, *J*_1_ = *J*_2_ = 6.2 Hz y *J*_3_ = 6.2 Hz, CH_2_-imidazole), 5.61 (1H, t, *J*_1_ = *J*_2_ = 5.8 Hz, NH), 7.97 (2H, m, Ar), 7.84 (2H, m, Ar), 7.26 (2H, m, Ar), 7.15 (2H, m, Ar). ^13^C RMN (75 MHz, CDCl_3_) *δ* = 136.33 (CH, N=CH–N), 114.07 (CH, N–CH=C), 142.04 (C, N–CH=CN), 27.44 (CH_2_, CH_2_–CH_2_-imidazole), 42.16 (CH_2_, CH_2_-imidazole), 133.73 (C, d, *J* = 3.4 Hz, Ar), 136.16 (C, d, *J* = 3.4 Hz, Ar), 129.69 (2 CH, d, *J* = 9.3 Hz, Ar), 130.43 (2 CH, d, *J* = 9.9 Hz, Ar), 116.28 (2 CH, d, *J* = 22.4 Hz, Ar), 117.43 (2CH, d, *J* = 23.0 Hz, Ar), 164.99 (CF, d, *J* = 254.6 Hz, Ar), 166.37 (CF, d, *J* = 259.6 Hz, Ar). ^19^F NMR (282 MHz, CDCl_3_): *δ* = −100.16 (m, 1F), −105.46 (m, 1F). HRMS: found *m*/*z* = 428.0544, calculated *m*/*z* for C_17_H_16_F_2_N_3_O_4_S_2_ [M + H]^+^ 428.0545 (0.1 mDa error). MS-MS showed the fragment ion corresponding to 4-fluorobenzenesulfonyl histamine at *m*/*z* = 269 and to the 4-fluorobenzenesulfonyl radical at *m*/*z* = 159.

##### *N* ^α^,*N*^τ^-di-4-(trifluoromethyl)benzenesulfonyl histamine (II-d)

The general procedure was followed employing 1.0 eq. of 4-(trifluoromethyl)benzenesulfonyl chloride, to afford 122.0 mg of II-d (51% final yield). ^1^H RMN (300 MHz, CDCl_3_) *δ* = 7.96 (1H, s, N=CH–N), 7.06 (1H, s, N–CH=C), 2.70 (2H, t, *J*_1_ = *J*_2_ = 6.2 Hz, CH_2_–CH_2_-imidazole), 3.27 (2H, q, *J*_1_ = *J*_2_ = 6.2 Hz y *J*_3_ = 6.2 Hz, CH_2_-imidazole), 5.71 (1H, t, *J*_1_ = *J*_2_ = 5.83 Hz, NH), 7.76 (2H, d, *J* = 8.3 Hz, Ar), 7.86 (2H, d, *J* = 8.3 Hz, Ar), 7.92 (2H, d, *J* = 8.3 Hz, Ar) 8.07 (2H, d, *J* = 8.3 Hz, Ar). ^13^C RMN (75 MHz, CDCl_3_) *δ* = 136.59 (CH, N=CH–N), 114.28 (CH, N–CH=C), 142.45 (C, N–CH=CN), 27.59 (CH_2_, CH_2_–CH_2_-imidazole), 42.30 (CH_2_, CH_2_-imidazole), 141.32 (C, Ar), 134.49 (CCF_3_, m, Ar), 127.62 (2 CH, Ar), 126.41 (2 CH, m, Ar), 143.92 (C, Ar), 136.78 (CCF_3_, m, Ar), 128.11 (2 CH, Ar), 127.29 (2CH, m, Ar), 121.28 (CF_3_, d, *J* = 273.0 Hz), 124.92 (CF_3_, d, *J* = 273.0 Hz). ^19^F NMR (282 MHz, CDCl_3_): *δ* = −63.13 (s, 3F), −63.45 (s, 3F). HRMS: found *m*/*z* = 550.0295, calculated *m*/*z* for C_19_H_16_F_6_NaN_3_O_4_S_2_ [M + Na]^+^ 550.0300 (0.5 mDa error). MS-MS showed the fragment ion corresponding to 4-(trifluoromethyl)benzenesulfonyl histamine at *m*/*z* = 319 and to the 4-(trifluoromethyl)benzenesulfonyl radical at *m*/*z* = 209.

##### *N* ^α^,*N*^τ^-di-2-fluor-5-methylbenzenesulfonyl histamine (II-e)

The general procedure was followed employing 1.0 eq. of 2-fluor-5-methylbenzenesulfonyl chloride to afford 115.8 mg of II-e (81% final yield). ^1^H RMN (300 MHz, CDCl_3_) *δ* = 7.96 (1H, s, N=CH–N), 7.06 (1H, s, N–CH=C), 2.71 (2H, t, *J*_1_ = *J*_2_ = 6.2 Hz, CH_2_–CH_2_-imidazole), 3.30 (2H, q, *J*_1_ = *J*_2_ = 6.2 Hz y *J*_3_ = 6.2 Hz, CH_2_-imidazole), 5.52 (1H, t, *J*_1_ = *J*_2_ = 5.8 Hz, NH), 2.37 (3H, s,CH_3_–Ar), 2.43 (3H, s, CH_3_–Ar), 7.02 (1H, dd, *J*_1_ = *J*_2_ = 8.3 Hz y *J*_3_ = 10.0 Hz Ar), 7.12 (1H, dd, *J*_1_ = *J*_2_ = 8.6 Hz y *J*_3_ = 10.1 Hz, Ar), 7.33 (1H, m, Ar), 7.48 (1H, m, Ar), 7.67 (1H, dd, *J*_1_ = *J*_2_ = 2.1 Hz y *J*_3_ = 6.9 Hz, Ar), 7.80 (1H, dd, *J*_1_ = *J*_2_ = 2.1 Hz y *J*_3_ = 6.9 Hz, Ar). ^13^C RMN (75 MHz, CDCl_3_) *δ* = 136.94 (CH, N=CH–N), 114.27 (CH, N–CH=C), 141.29 (C, N–CH=CN), 27.66 (CH_2_, CH_2_–CH_2_-imidazole), 42.18 (CH_2_, CH_2_-imidazole), 125.26 (C, d, *J* = 13.1 Hz, Ar), 156.92 (CF, d, *J* = 252.0 Hz, Ar), 116.59 (CH, d, *J* = 21.3 Hz, Ar), 135.24 (CH, d, *J* = 8.4 Hz, Ar), 134.38 (C, d, *J* = 3.8 Hz, Ar), 129.88 (CH, Ar), 127.36 (C, d, *J* = 13.7 Hz, Ar), 157.28 (CF, d, *J* = 252.0 Hz, Ar), 117.65 (CH, d, *J* = 21.3 Hz, Ar), 138.05 (CH, d, *J* = 8.4 Hz, Ar), 135.39 (C, d, *J* = 3.8 Hz, Ar), 130.29 (CH, Ar), 20.58 (ArCH_3_), 20.64 (ArCH_3_). ^19^F NMR (282 MHz, CDCl_3_): *δ* = −112.59 (m, F), −116.21 (m, F). HRMS: found *m*/*z* = 456.0859, calculated *m*/*z* for C_19_H_20_F_2_N_3_O_4_S_2_ [M + H]^+^ 456.0858 (0.1 mDa error). MS-MS showed the fragment ion corresponding to 2-fluor-5-methylbenzenesulfonyl histamine at *m*/*z* = 283 and to the 2-fluor-5-methylbenzenesulfonyl radical at *m*/*z* = 173.

##### *N* ^α^,*N*^τ^-di-2-chloro-4-fluorobenzenesulfonyl histamine (II-f)

The general procedure was followed employing 1.0 eq. of 2-chloro-4-fluorobenzenesulfonyl chloride to afford 83.8 mg of II-f (81% final yield). ^1^H RMN (300 MHz, CDCl_3_) *δ* = 8.03 (1H, s, N=CH–N), 6.99 (1H, s, N–CH=C), 2.70 (2H, t, *J*_1_ = *J*_2_ = 6.25 Hz, CH_2_–CH_2_-imidazole), 3.25 (2H, q, *J*_1_ = *J*_2_ = 6.2 Hz y *J*_3_ = 6.2 Hz, CH_2_-imidazole), 5.80 (1H, t, *J*_1_ = *J*_2_ = 5.83 Hz, NH), 7.11 (1H, m, Ar), 7.20–7.24 (2H, m, Ar), 7.31 (1H, dd, *J*_1_ = *J*_3_ 2.6 Hz y *J*_2_ = 8.0 Hz, Ar), 8.09 (1H, dd, *J*_1_ = *J*_2_ 5.7 Hz y *J*_3_ = 8.8 Hz, Ar), 8.26 (1H, dd, *J*_1_ = *J*_2_ 5.7 Hz y *J*_3_ = 8.8 Hz, Ar). ^13^C RMN (75 MHz, CDCl_3_) *δ* = 131.58 (CH, N=CH–N), 114.49 (CH, N–CH=C), 141.36 (C, N–CH=CN), 27.50 (CH_2_, CH_2_–CH_2_-imidazole), 42.17 (CH_2_, CH_2_-imidazole), 131.58 (C, *J* = 3.3 Hz, Ar), 133.35 (CCl, Ar), 119.20 (CH, d, *J* = 25.5 Hz, Ar), 164.63 (CF, d, *J* = 258 Hz, Ar), 114.44 (CH, d, *J* = 21.4 Hz, Ar), 133.28 (CH, d, *J* = 9.6 Hz, Ar), 133.62 (C, d, *J* = 3.3 Hz, Ar), 135.31 (CCl, d, *J* = 10.3 Hz, Ar), 120.49 (CH, d, *J* = 25.6 Hz, Ar), 165.95 (CF, d, *J* = 263 Hz, Ar), 115.42 (CH, d, *J* = 21.9 Hz, Ar), 133.93 (CH, d, *J* = 10.3 Hz, Ar). ^19^F NMR (282 MHz, CDCl_3_): *δ* = −98.55 (m, F), −103.82 (m, F). HRMS: found *m*/*z* = 517.9586, calculated *m*/*z* for C_17_H_14_Cl_2_F_2_N_3_NaO_4_S_2_ [M + Na]^+^ 517.9585 (0.1 mDa error). MS-MS showed the fragment ion corresponding to 2-chloro-4-fluorobenzenesulfonyl histamine at *m*/*z* = 301 and to the 2-chloro-4-fluorobenzenesulfonyl radical at *m*/*z* = 192.

##### *N* ^α^,*N*^τ^-di-3,4,5-trifluorobenzenesulfonyl histamine (II-g)

The general procedure was followed employing 0.9 eq. of 3,4,5-trifluorobenzenesulfonyl chloride to afford 140.4 mg of II-g (63% final yield). ^1^H RMN (300 MHz, acetone-*d*_6_) *δ* = 8.14 (1H, s, N=CH–N), 7.42 (1H, s, N–CH=C), 2.71 (2H, t, *J*_1_ = *J*_2_ = 6.2 Hz, CH_2_–CH_2_-imidazole), 3.28 (2H, q, *J*_1_ = *J*_2_ = 6.2 Hz y *J*_3_ = 6.2 Hz, CH_2_-imidazole), 6.86 (1H, t, *J*_1_ = *J*_2_ = 5.8 Hz, NH), 7.63 (2H, m, Ar), 8.02 (2H, m, Ar). ^13^C RMN (75 MHz, acetone-*d*_6_) *δ* = 137.71 (CH, N=CH–N), 115.46 (CH, N–CH=C), 143.20 (C, N–CH=CN), 28.96 (CH_2_, CH_2_–CH_2_-imidazole), 42.95 (CH_2_, CH_2_-imidazole), 134.86 (C, m, Ar), 138.50 (C, m, Ar), 113.01 (2 CH, m, Ar), 114.37 (2 CH, m, Ar), 150.07 (CF, m, Ar), 150.45 (CF, m, Ar), 153.42 (CF, m, Ar), 153.85 (CF, m, Ar), Ar), 142.24 (CF, Ar), 145.76 (CF, Ar). ^19^F NMR (282 MHz, acetone-*d*_6_): *δ* = −130.66 (m, 2F), −132.85 (m, 2F), −150.90 (m, F), −156.16 (m, F). HRMS: found *m*/*z* = 500.0167, calculated *m*/*z* for C_17_H_12_F_6_N_3_O_4_S_2_ [M + H]^+^ 500.0168 (0.1 mDa error). MS-MS showed the fragment ion corresponding to 3,4,5-trifluorobenzenesulfonyl histamine at *m*/*z* = 303 and to the 3,4,5-trifluorobenzenesulfonyl radical at *m*/*z* = 195.

##### *N* ^α^,*N*^τ^-di-2-(trifluoromethyl)-4-fluorobenzenesulfonyl histamine (II-h)

The general procedure was followed employing 0.9 eq. of 2-(trifluoromethyl)-4-fluorobenzenesulfonyl chloride to afford 150.9 mg of II-h (60% final yield). ^1^H RMN (300 MHz, CDCl_3_) *δ* = 7.91 (1H, s, N=CH–N), 7.00 (1H, s, N–CH=C), 2.72 (2H, t, *J*_1_ = *J*_2_ = 6.2 Hz, CH_2_–CH_2_-imidazole), 3.28 (2H, q, *J*_1_ = *J*_2_ = 6.2 Hz y *J*_3_ = 6.2 Hz, CH_2_-imidazole), 5.58 (1H, t, *J*_1_ = *J*_2_ = 5.8 Hz, NH), 7.36 (1H, m, Ar), 7.50 (1H, m, Ar), 7.54 (1H, m, Ar), 7.65 (1H, dd, *J*_1_ = *J*_3_ 2.6 Hz y *J*_2_ = 8.6 Hz, Ar), 8.20–8.27 (2H, m, Ar). ^13^C RMN (75 MHz, CDCl_3_) *δ* = 137.04 (CH, N=CH–N), 114.47 (CH, N–CH=C), 141.52 (C, N–CH=CN), 27.55 (CH_2_, CH_2_–CH_2_-imidazole), 42.22 (CH_2_, CH_2_-imidazole), 116.74 (CH, m, Ar), 117.54 (CH, m, Ar), 118.88 (CH, d, *J* = 21.4 Hz, Ar), 120.23 (CH, d, *J* = 21.4 Hz, Ar), 119.83 (C, d, *J* = 60.1 Hz, Ar), 123.49 (C, d, *J* = 60.1 Hz, Ar), 130.27 (CF_3_, m, Ar), 131.67 (CF_3_, m, Ar), 132.67 (C, d, *J* = 3.3 Hz, Ar), 134.88 (C, d, *J* = 3.3 Hz, Ar), 134.56 (CH, d, *J* = 9.3 Hz, Ar), 135.28 (CH, d, *J* = 9.3 Hz, Ar), 164.15 (CF, d, *J* = 257.3 Hz, Ar), 165.51 (CF, d, *J* = 262.4 Hz, Ar). ^19^F NMR (282 MHz, CDCl_3_): *δ* = −58.10 (m, CF_3_), −58.68 (m, CF_3_), −97.98 (m, F), −103.51 (m, F). HRMS: found *m*/*z* = 564.0297, calculated *m*/*z* for C_19_H_14_F_8_N_3_O_4_S_2_ [M + H]^+^ 564.0292 (0.5 mDa error). MS-MS showed the fragment ion corresponding to 2-(trifluoromethyl)-4-fluorobenzenesulfonyl histamine at *m*/*z* = 337 and to the 2-(trifluoromethyl)-4-fluorobenzenesulfonyl radical at *m*/*z* = 227.

##### *N* ^α^,*N*^τ^-di-pentafluorobenzenesulfonyl histamine (II-i)

The general procedure was followed employing 1.0 eq. of pentafluorobenzenesulfonyl chloride to afford 140.2 mg of II-i (49% final yield). ^1^H RMN (300 MHz, acetone-*d*_6_) *δ* = 8.16 (1H, s, N=CH–N), 7.43 (1H, s, N–CH=C), 2.81 (2H, t, *J*_1_ = *J*_2_ = 6.2 Hz, CH_2_–CH_2_-imidazole), 3.45 (2H, q, *J*_1_ = *J*_2_ = 6.2 Hz y *J*_3_ = 6.2 Hz, CH_2_-imidazole), 7.54 (1H, t, *J*_1_ = *J*_2_ = 5.83 Hz, NH). ^13^C RMN (75 MHz, acetone-*d*_6_) *δ* = 137.69 (CH, N=CH–N), 115.41 (CH, N–CH=C), 143.09 (C, N–CH=CN), 28.89 (CH_2_, CH_2_–CH_2_-imidazole), 42.92 (CH_2_, CH_2_-imidazole), 114.56 (C, m, Ar), 117.80 (C, m, Ar), 137.00–137.93 (2 CF, m, Ar), 140.35–141.27 (2 CF, m, Ar), 142.51–145.28 (3 CF, m, Ar), 146.02–148.74 (3CF, m, Ar).^19^F NMR (282 MHz, acetone-*d*_6_): *δ* = −136.91 (m, 2F), −139.34 (m, 2F), −144.49 (m, F), −150.32 (m, F), −160.33 (m, 2F), −162.08 (m, 2F). HRMS: found *m*/*z* = 571.9788, calculated *m*/*z* for C_17_H_8_F_10_N_3_O_4_S_2_ [M + H]^+^ 571.9791 (0.3 mDa error). MS-MS showed the fragment ion corresponding to pentafluorobenzenesulfonyl histamine at *m*/*z* = 341 and to the pentafluorobenzenesulfonyl radical at *m*/*z* = 230.

##### *N* ^α^,*N*^τ^-di-3-fluoro-4-methoxybenzenesulfonyl histamine (II-j)

The general procedure was followed employing 1.0 eq. of 3-fluoro-4-methoxybenzenesulfonyl chloride to afford 100.2 mg of II-j (46% final yield). ^1^H RMN (300 MHz, CDCl_3_) *δ* = 7.88 (1H, s, N=CH–N), 6.99 (1H, s, N–CH=C), 2.67 (2H, t, *J*_1_ = *J*_2_ = 6.2 Hz, CH_2_–CH_2_-imidazole), 3.22 (2H, q, *J*_1_ = *J*_2_ = 6.2 Hz y *J*_3_ = 6.2 Hz, CH_2_-imidazole), 5.26 (1H, t, *J*_1_ = *J*_2_ = 5.8 Hz, NH), 3.95 (3H, s, CH_3_O–Ar), 3.97 (3H, s, CH_3_O–Ar), 7.02 (1H, t, *J* = 8.3 Hz, Ar), 7.10 (1H, t, *J* = 8.3 Hz, Ar), 7.52 (1H, m, Ar), 7.57–7.64 (2H, m, Ar), 7.73 (1H, m, Hz, Ar). ^13^C RMN (75 MHz, CDCl_3_) *δ* = 136.28 (CH, N=CH–N), 114.00 (CH, N–CH=C), 141.95 (C, N–CH=CN), 27.33 (CH_2_, CH_2_–CH_2_-imidazole), 42.20 (CH_2_, CH_2_-imidazole), 132.04 (C, *J* = 5.4 Hz, Ar), 115.17 (CH, Ar), 151.80 (CF, d, *J* = 254.7 Hz, Ar), 153.46 (C, d, *J* = 10.5 Hz, Ar), 113.55 (CH, Ar), 125.22 (CH, d, *J* = 3.5 Hz, Ar), 128.96 (C, *J* = 5.9 Hz, Ar), 151.66 (CF, d, *J* = 254.6 Hz, Ar), 151.30 (C, d, *J* = 10.9 Hz, Ar), 112.88 (CH, Ar), 124.18 (CH, d, *J* = 3.5 Hz, Ar), 56.65 (OCH_3_), 56.41 (OCH_3_). ^19^F NMR (282 MHz, CDCl_3_): *δ* = −129.48 (m, F), −131.93 (m, F). HRMS: found *m*/*z* = 488.0760, calculated *m*/*z* for C_19_H_20_F_2_N_3_O_6_S_2_ [M + H]^+^ 488.0756 (0.4 mDa error). MS-MS showed the fragment ion corresponding to 3-fluoro-4-methoxybenzenesulfonyl histamine at *m*/*z* = 299 and to the 3-fluoro-4-methoxybenzenesulfonyl radical at *m*/*z* = 189.

### *N* ^α^-arylsulfonyl histamine analogues synthesis

#### General procedure

A solution of *N*^α^,*N*^τ^-di-arylsulfonyl histamine (0.0635 mmol) and K_2_CO_3_ (0.0635 mmol) in ethanol : water (1 : 1) (5 mL) was stirred under reflux during 2 h. The solvent was eliminated under reduced pressure and the resulting residue, chromatographed on silica gel in isocratic condition with DCM : MeOH (9 : 1) to obtain I-a to I-j.

##### *N* ^α^-benzenesulfonyl histamine (I-a)

The general procedure was followed employing 0.1 eq of *N*^α^,*N*^τ^-di-benzenesulfonyl histamine to afford 101.2 mg of I-a (82% final yield). ^1^H RMN (300 MHz, acetone-*d*_6_) *δ* = 7.85 (2H, m, Ar), 7.54–7.66 (4H, m, Ar), 6.83 (1H, s, N–CH=C), 2.70 (2H, t, *J*_1_ = *J*_2_ = 6.6 Hz, CH_2_–CH_2_-imidazole), 3.16 (2H, q, *J*_1_ = *J*_2_ = 6.6 Hz y *J*_3_ = 5.7 Hz, CH_2_-imidazole), 6.95 (1H, NH). ^13^C RMN (75 MHz, acetone-*d*_6_) *δ* = 135.78 (CH, N=CH–N), 115.62 (CH, N–CH=C), 137.59 (C, N–CH=CN), 27.89 (CH_2_, CH_2_–CH_2_-imidazole), 44.38 (CH_2_, CH_2_-imidazole), 141.95 (C, Ar), 127.80 (2CH, Ar), 129.96 (2 CH, Ar), 133.13 (CH, Ar). HRMS: found *m*/*z* = 252.0798, calculated *m*/*z* for C_11_H_14_N_3_O_2_S [M + H]^+^ 252.0801 (0.3 mDa error). MS-MS showed the fragment ion corresponding benzenesulfonyl radical at *m*/*z* = 141.

##### *N* ^α^-*p*-toluenesulfonyl histamine (I-b)

The general procedure was followed employing 0.1 eq. of *N*^α^,*N*^τ^-di-*p*-toluenesulfonyl histamine to afford 17.1 mg of I-b (64% final yield). ^1^H RMN (300 MHz, acetone-*d*_6_) *δ* = 7.57 (1H, s, N=CH–N), 6.84 (1H, s, N–CH=C), 2.70 (2H, t, *J*_1_ = *J*_2_ = 6.7 Hz, CH_2_–CH_2_-imidazole), 3.13 (2H, q, *J*_1_ = *J*_2_ = 6.7 Hz y *J*_3_ = 5.2 Hz, CH_2_-imidazole), 6.92 (1H, NH), 7.72 (2H, d, *J* = 8.3 Hz, Ar), 7.37 (2H, d, *J* = 8.3 Hz, Ar), 2.40 (3H, s, CH_3_-Ar). ^13^C RMN (75 MHz, acetone-*d*_6_) *δ* = 135.70 (CH, N=CH–N), 115.68 (CH, N–CH=C), 137.38 (C, N–CH=CN), 27.84 (CH_2_, CH_2_–CH_2_-imidazole), 44.30 (CH_2_, CH_2_-imidazole), 139.10 (C, Ar), 127.82 (2 CH, Ar), 130.39 (2 CH, Ar), 143.69 (C, Ar), 21.38 (CH_3_, Ar-CH_3_). HRMS: found *m*/*z* = 266.0961, calculated *m*/*z* for C_12_H_16_N_3_O_2_S [M + H]^+^ 266.0958 (0.3 mDa error). MS-MS showed the fragment ion corresponding to the *p*-toluenesulfonyl radical at *m*/*z* = 155.

##### *N* ^α^-4-fluorobenzenesulfonyl histamine (I-c)

The general procedure was followed employing 0.1 eq. of *N*^α^,*N*^τ^-di-4-fluorobenzenesulfonyl histamine to afford 116.7 mg of I-c (60% final yield). ^1^H RMN (300 MHz, CD_3_OD) *δ* = 7.54 (1H, s, N=CH–N), 6.80 (1H, s, N–CH=C), 2.72 (2H, t, *J* = 7.3 Hz, CH_2_-imidazole), 3.12 (2H, t, *J* = 7.3 Hz, CH_2_–CH_2_-imidazole), 7.28 (2H, m, Ar), 7.87 (2H, m, Ar). ^13^C RMN (75 MHz, CD_3_OD) *δ* = 136.08 (CH, N=CH–N), 117.49 (CH, N–CH=C), 135.93 (C, N–CH=CN), 28.49 (CH_2_, CH_2_–CH_2_-imidazole), 43.99 (CH_2_, CH_2_-imidazole), 138.31 (C, *J* = 3.0 Hz, Ar), 130.87 (2CH, *J* = 9.5 Hz, Ar), 117.14 (2CH, *J* = 22.6 Hz, Ar), 166.32 (CF, *J* = 252.1 Hz, Ar). ^19^F NMR (282 MHz, CD_3_OD): *δ* = −108.75 (m, 1F). HRMS: found *m*/*z* = 270.0710, calculated *m*/*z* for C_11_H_13_FN_3_O_2_S [M + H]^+^ 270.0707 (0.3 mDa error). MS-MS showed the fragment ion corresponding to the 4-fluorobenzenesulfonyl radical at *m*/*z* = 159.

##### *N* ^α^-4-(trifluoromethyl)benzenesulfonyl histamine (I-d)

The general procedure was followed employing 0.1 eq. of *N*^α^,*N*^τ^-di-4-(trifluoromethyl)benzenesulfonyl histamine to afford 122.0 mg of I-d (61% final yield). ^1^H RMN (300 MHz, acetone-*d*_6_) *δ* = 7.56 (1H, s, N=CH–N), 6.85 (1H, s, N–CH=C), 2.73 (2H, t, *J*_1_ = 6.90 Hz, CH_2_-imidazole), 3.22 (2H, t, *J*_1_ = 6.90 Hz, CH_2_–CH_2_-imidazole), 7.93 (2H, d, *J* = 8.10 Hz, Ar), 8.06 (2H, d, *J* = 8.10 Hz, Ar). ^13^C RMN (75 MHz, acetone-*d*_6_) *δ* = 134.87 (CH, N=CH–N), 114.71 (CH, N–CH=C), 136.40 (C, N–CH=CN), 26.99 (CH_2_, CH_2_–CH_2_-imidazole), 43.37 (CH_2_, CH_2_-imidazole), 144.98 (C, Ar), 133.01 (CCF_3_, *J* = 32.5 Hz, Ar), 127.69 (2 CH, Ar), 126.19 (2 CH, *J* = 3.7 Hz, Ar), 123.74 (CF_3_, *J* = 272.1 Hz). ^19^F NMR (282 MHz, acetone-*d*_6_): *δ* = −63.54 (s, 3F, CF_3_). HRMS: found *m*/*z* = 320.0670, calculated *m*/*z* for C_12_H_13_F_3_N_3_O_2_S [M + H]^+^ 320.0675 (0.5 mDa error). MS-MS showed the fragment ion corresponding to the 4-(trifluoromethyl)benzenesulfonyl radical at *m*/*z* = 209.

##### *N* ^α^-2-fluor-5-methylbenzenesulfonyl histamine (I-e)

The general procedure was followed employing 0.1 eq. of *N*^α^,*N*^τ^-di-2-fluor-5-methylbenzenesulfonyl histamine to afford 98.0 mg of I-e (89% final yield). ^1^H RMN (300 MHz, CD_3_OD) *δ* = 7.52 (1H, s, N=CH–N), 6.78 (1H, s, N–CH=C), 2.73 (2H, t, *J*_1_ = 7.3 Hz, CH_2_-imidazole), 3.19 (2H, t, *J*_1_ = 7.3 Hz, CH_2_–CH_2_-imidazole), 2.38 (3H, s, CH_3_–Ar), 7.14 (1H, dd, *J*_1_ = *J*_2_ = 8.6 Hz y *J*_3_ = 10.2 Hz, Ar), 7.43 (1H, m, Ar), 7.63 (1H, dd, *J*_1_ = *J*_2_ = 2.1 Hz y *J*_3_ = 6.4 Hz, Ar). ^13^C RMN (75 MHz, CD_3_OD) *δ* = 136.07 (CH, N=CH–N), 117.64 (CH, N–CH=C), 136.07 (C, N–CH=CN), 28.57 (CH_2_, CH_2_–CH_2_-imidazole), 43.89 (CH_2_, CH_2_-imidazole), 129.33 (C, *J* = 13.7 Hz, Ar), 158.38 (CF, *J* = 250.9 Hz, Ar), 117.77 (CH, *J* = 22.1 Hz, Ar), 136.38 (CH, *J* = 8.3 Hz, Ar), 135.83 (C, Ar), 131.09 (CH, Ar), 20.54 (Ar–CH_3_). ^19^F NMR (282 MHz, CD_3_OD): *δ* = −117.24 (m, F). HRMS: found *m*/*z* = 284.08639, calculated *m*/*z* for C_12_H_15_FN_3_O_2_S [M + H]^+^ 284.08635 (0.04 mDa error). MS-MS showed the fragment ion corresponding to the 2-fluor-5-methylbenzenesulfonyl radical at *m*/*z* = 173.

##### *N* ^α^-2-Chloro-4-fluorobenzenesulfonyl histamine (I-f)

The general procedure was followed employing 0.1 eq. of *N*^α^,*N*^τ^-di-2-chloro-4-fluorobenzenesulfonyl histamine to afford 85.8 mg of I-f (70% final yield). ^1^H RMN (300 MHz, acetone-*d*_6_) *δ* = 7.60 (1H, s, N=CH–N), 6.87 (1H, s, N–CH=C), 2.74 (2H, t, *J* = 6.4 Hz, CH_2_-imidazole), 3.19 (2H, t, *J* = 6.4 Hz, CH_2_–CH_2_-imidazole), 7.33 (1H, m, Ar), 7.47 (1H, dd, *J*_1_ = *J*_2_ = 2.4 Hz y *J*_3_ = 8.6 Hz, Ar), 8.12 (1H, dd, *J*_1_ = *J*_2_ = 6.0 Hz y *J*_3_ = 8.9 Hz, Ar). ^13^C RMN (75 MHz, acetone-*d*_6_) *δ* = 135.77 (CH, N=CH–N), 115.38 (CH, N–CH=C), 137.46 (C, N–CH=CN), 27.69 (CH_2_, CH_2_–CH_2_-imidazole), 44.16 (CH_2_, CH_2_-imidazole), 135.61 (C, *J* = 3.5 Hz, Ar), 133.99 (CCl, Ar), 115.28 (CH, *J* = 21.6 Hz, Ar), 165.22 (CF, *J* = 254.6 Hz, Ar), 119.86 (CH, *J* = 25.9 Hz, Ar), 134.19 (CH, *J* = 9.8 Hz, Ar). ^19^F NMR (282 MHz, acetone-*d*_6_): *δ* = −106.97 (m, F). HRMS: found *m*/*z* = 304.0320, calculated *m*/*z* for C_11_H_12_ClFN_3_O_2_S [M + H]^+^ 304.0317 (0.3 mDa error). MS-MS showed the fragment ion corresponding to the 2-chloro-4-fluorobenzenesulfonyl radical at *m*/*z* = 192.

##### *N* ^α^-3,4,5-trifluorobenzenesulfonyl histamine (I-g)

The general procedure was followed employing 0.1 eq. of *N*^α^,*N*^τ^-di-3,4,5-trifluorobenzenesulfonyl histamine to afford 21.0 mg of I-g (69% final yield). ^1^H RMN (300 MHz, acetone-*d*_6_) *δ* = 7.58 (3H, m, N=CH–N and 2H Ar), 6.87 (1H, s, N–CH=C), 2.74 (2H, t, *J* = 6.9 Hz, CH_2_-imidazole), 3.25 (2H, t, *J* = 6.9 Hz, CH_2_–CH_2_-imidazole), 7.62 (2H, m, Ar). ^13^C RMN (75 MHz, acetone-*d*_6_) *δ* = 135.79 (CH, N=CH–N), 115.78 (CH, N–CH=C), 137.13 (C, N–CH=CN), 27.91 (CH_2_, CH_2_–CH_2_-imidazole), 42.22 (CH_2_, CH_2_-imidazole), 138.54 (C, m, Ar), 113.03 (2CH, m, Ar), 142.92 (CF, m, Ar), 151.64 (2CF, m, Ar). ^19^F NMR (282 MHz, acetone-*d*_6_): *δ* = −133.02 (m, 2F), −156.48 (m, F). HRMS: found *m*/*z* = 306.0532, calculated *m*/*z* for C_11_H_11_F_3_N_3_O_2_S [M + H]^+^ 306.0524 (0.8 mDa error). MS-MS showed the fragment ion corresponding to the 3,4,5-trifluorobenzenesulfonyl radical at *m*/*z* = 195.

##### *N* ^α^-2-(trifluoromethyl)-4-fluorobenzenesulfonyl histamine (I-h)

The general procedure was followed employing 0.1 eq. of *N*^α^,*N*^τ^-di-2-(trifluoromethyl)-4-fluorobenzenesulfonyl histamine to afford 31.3.0 mg of I-h (85% final yield). ^1^H RMN (300 MHz, acetone-*d*_6_) *δ* = 8.26 (1H, dd, *J*_1_ = *J*_2_ = 5.5H y *J*_3_ = 5.8 Hz, Ar), 7.73 (1H, m, Ar), 7.55–7.65 (1H, m, Ar), 7.58 (1H, s, N=CH–N), 6.86 (1H, s, N–CH=C), 3.28 (2H, t, *J*_1_ = *J*_2_ = 6.8 Hz, CH_2_-imidazole), 2.77 (2H, t, *J*_1_ = *J*_2_ = 6.8 Hz, CH_2_–CH_2_-imidazole). ^13^C RMN (75 MHz, CDCl_3_) *δ* = 135.53 (CH, N=CH–N), 115.40 (CH, N–CH=C), 136.85 (C, N–CH=CN), 27.63 (CH_2_, CH_2_–CH_2_-imidazole), 44.99 (CH_2_, CH_2_-imidazole), 136.67 (C, *J* = 3.3 Hz, Ar), 130.35 (CCF_3_, m, Ar), 116.91 (CH, m, Ar), 164.42 (CF, *J* = 253.9 Hz, Ar), 119.82 (CH, *J* = 21.5 Hz, Ar), 134.94 (CH, *J* = 8.9 Hz, Ar), 123.21 (CF_3_, *J* = 275.7 Hz, Ar). ^19^F NMR (282 MHz, acetone-*d*_6_): *δ* = −58.56 (m, CF_3_), −106.67 (m, F). HRMS: found *m*/*z* = 338.0591, calculated *m*/*z* for C_12_H_12_F_4_N_3_O_2_S [M + H]^+^ 338.0586 (0.5 ppm mDa). MS-MS showed the fragment ion corresponding to the 2-(trifluoromethyl)-4-fluorobenzenesulfonyl radical at *m*/*z* = 227.

##### *N* ^α^-pentafluorobenzenesulfonyl histamine (I-i)

The general procedure was followed employing 0.1 eq. of *N*^α^,*N*^τ^-di-2 pentafluorobenzenesulfonyl histamine to afford 21.1 mg of I-i (61% final yield). ^1^H RMN (300 MHz, acetone-*d*_6_) *δ* = 6.90 (1H, s, N=CH–N), 7.58 (1H, s, N–CH=C), 2.81 (2H, t, *J*_1_ = *J*_2_ = 6.8 Hz, CH_2_–CH_2_-imidazole), 3.43 (2H, t, *J*_1_ = *J*_2_ = 6.8 Hz, CH_2_–CH_2_-imidazole). ^13^C RMN (75 MHz, acetone-*d*_6_) *δ* = 134.87 (CH, N=CH–N), 114.87 (CH, N–CH=C), 142.79 (C, N–CH=CN), 26.81 (CH_2_, CH_2_–CH_2_-imidazole), 43.23 (CH_2_, CH_2_-imidazole), 142.79 (C, Ar), 117.10 (2 CF, m, Ar), 139.54 (2CF, m, Ar), 146.36 (CF, Ar). ^19^F NMR (282 MHz, acetone-*d*_6_): *δ* = −141.08 (m, 2F), −150.73 (m, F), 157.64 (m, 2F). HRMS: found *m*/*z* = 342.0334, calculated *m*/*z* for C_17_H_12_F_6_N_3_O_4_S_2_ [M + H]^+^ 342.0330 (0.4 mDa error). MS-MS showed the fragment ion corresponding to the pentafluorobenzenesulfonyl radical at *m*/*z* = 231.

##### *N* ^α^-3-fluoro-4-methoxybenzenesulfonyl histamine (I-j)

The general procedure was followed employing 0.1 eq. of *N*^α^,*N*^τ^-di-3-fluoro-4-methoxybenzenesulfonyl histamine to afford 22.7 mg of I-j (74% final yield). ^1^H RMN (300 MHz, acetone-*d*_6_) *δ* = 7.59 (1H, s, N=CH–N), 6.86 (1H, s, N–CH=C), 2.72 (2H, t, *J*_1_ = *J*_2_ = 6.25 Hz, CH_2_–CH_2_-imidazole), 3.16 (2H, q, *J*_1_ = *J*_2_ = 6.19 Hz y *J*_3_ = 6.22 Hz, CH_2_–CH_2_-imidazole), 7.06 (1H, m, NH), 3.97 (3H, s, CH_3_O–Ar), 7.29 (H, t, *J* = 8.4 Hz, Ar), 7.54 (1H, m, Ar), 7.59 (1H, m, Ar), 7.63 (1H, m, Ar). ^13^C RMN (75 MHz, acetone-*d*_6_) *δ* = 135.73 (CH, N=CH–N), 115.79 (CH, N–CH=C), 137.16 (C, N–CH=CN), 27.82 (CH_2_, CH_2_–CH_2_-imidazole), 42.21 (CH_2_, CH_2_–CH_2_-imidazole), 133.83 (C, d, *J* = 5.4 Hz, Ar), 115.42 (CH, d, *J* = 20.8 Hz, Ar), 152.25 (CF, d, *J* = 248.9 Hz, Ar), 151.82 (C, d, *J* = 10.5 Hz, Ar), 125.13 (CH, d, *J* = 3.7 Hz, Ar), 114.22 (CH, d, *J* = 1.4 Hz, Ar), 56.82 (CH_3_). ^19^F NMR (282 MHz, acetone-*d*_6_): *δ* = −134.22 (m, F). HRMS: found *m*/*z* = 300.0808, calculated *m*/*z* for C_12_H_15_FN_3_O_2_S [M + H]^+^ 300.0813 (0.5 mDa error). MS-MS showed the fragment ion corresponding to the 3-fluoro-4-methoxybenzenesulfonyl radical at *m*/*z* = 189.

### Microplate assay protocols

#### β-Glc

The hydrolysis of *p*-nitrophenyl-β-*O*-d-glucopyranoside (β-PNPG) was continuously measured in 96-well microplate according to Arnaldos *et al.*^38^ Wells were filled in triplicate with β-Glc (from almonds) in 0.1 M buffer phosphate, pH 7 (7.10 μU mL^−1^ final concentration in well); α-cyclodextrin, in 0.1 M buffer phosphate, pH 7 (1.22 mM end concentration in well) and 10 μL of test compound in dimethylsulfoxide (DMSO) solution (40 μM or 65 μM end concentration in well). Wells containing the corresponding volume of DMSO without inhibitor were used as reference of maximum enzymatic rates whilst 1-DNJ in DMSO (65 μM end concentration in well) was used as enzyme inhibition control. The final volume in well was 270 μL. The enzymatic reaction was initiated by addition of β-PNPG (1.63 mM end concentration in well). The plate was shaken for 2 s and the increase in absorbance at 405 nm was monitored at 37 °C for 10 min. For IC_50_ determination, ten serial dilutions of the compounds were prepared in DMSO, following equally spaced points in neperian logarithm scale, starting at 16.2 mM and finishing at 1.7 μM. IC_50_ was calculated using Prism V5.01 (GraphPad Software Inc., La Jolla, CA, USA) requesting the software a non-linear regression curve fit for a log[Inhibitor] *vs.* normalized answer model with variable slope.

#### TYR

According to the reported method by Atta-ur-Rahman,^44^ the formation of the DOPAchrome was continuously measured in 96-well microplate. Wells were filled in triplicates with: 10 μL test compound in DMSO solution (1.48 μM end concentration in well) and mushrooms TYR in 0.1 M buffer phosphate, pH 7 (15.55 U mL^−1^, end concentration in well). Wells containing the corresponding volume of DMSO without inhibitor were used as reference of maximum enzymatic rates, whilst kojic acid in DMSO (1.48 μM end concentration in well) was used as enzyme inhibition control. The final volume in well was 270 μL. The enzymatic reaction was initiated by addition of the substrate l-Tyr (0.63 mM, end concentration in well). The plate was shaken for 2 s and the increase in absorbance at 475 nm was monitored at 37 °C for 30 min.

#### XO

Following the method previously reported by Chu,^45^ the formation of uric acid was continuously measured in 96-well microplate. Each well was filled in triplicates with: of 10 μL test compound in DMSO (0.11 μM end concentration in well) and XO (bovine) in 0.2 M buffer phosphate, pH 7.5 (2.81 μU mL^−1^, end concentration in well). Wells containing the corresponding volume of DMSO without inhibitor were used as reference of maximum enzymatic rates, and allopurinol in DMSO (0.11 μM end concentration in well) was used as enzyme inhibition control. The final volume in well was 270 μL. The enzymatic reaction was initiated by addition of the xanthine substrate (0.04 mM, end concentration in well). The plate was shaken for 2 s and the increase in absorbance at 295 nm was monitored at 30 °C for 20 min.

#### AChE

AChE activity measurement was carried on based on Ellman's method.^46^ Wells were filled in triplicate with AChE (electric eel) in 0.1 M buffer phosphate, pH 7.5 (13.7 μU mL^−1^ end concentration in well), Ellman's reagent (DTNB) (5,5-dithio-bis-(2-nitrobenzoic acid), same buffer solution (0.31 mM end concentration in well) and 10 μL of test compound in DMSO solution (0.05 μM end concentration in well). Wells containing the corresponding volume of DMSO without inhibitor were used as reference of maximum enzymatic rates, and eserine in DMSO (0.05 μM end concentration in well) was used as control for enzyme inhibition. The final volume in well was 270 μL. The enzymatic reaction was initiated by addition of acetylthiocholine iodide (ATCI) (0.46 mM end concentration in well). The plate was shaken for 2 s and the increase in absorbance at 405 nm was monitored at 37 °C for 15 min.

#### α-Glc

Similar to the method applied in β-Glc assay, the hydrolysis of *p*-nitrophenyl-α-*O*-d-glucopyranoside (α-PNPG) was continuously measured in 96-well microplate. Wells were filled in triplicate with α-Glc (yeast) in 0.1 M buffer phosphate, pH 7 (7.10 μU mL^−1^ final concentration in well); α-cyclodextrin, in 0.1 M buffer phosphate, pH 7 (1.22 mM end concentration in well) and 10 μL of test compound in dimethylsulfoxide (DMSO) solution (383 μM or 2397 μM end concentration in well). Wells containing the corresponding volume of DMSO without inhibitor were used as reference of maximum enzymatic rates whilst 1-DNJ in DMSO (333 μM end concentration in well) was used as enzyme inhibition control. The final volume in well was 270 μL. The enzymatic reaction was initiated by addition of β-PNPG (1.63 mM end concentration in well). The plate was shaken for 2 s and the increase in absorbance at 405 nm was monitored at 37 °C for 10 min. For IC_50_ determination, ten serial dilutions of the compounds were prepared in DMSO, following equally spaced points in neperian logarithm scale, starting at 64.73 mM and finishing at 6.4 μM. IC_50_ was calculated using Prism V5.01 (GraphPad Software Inc., La Jolla, CA, USA) requesting the software a non-linear regression curve fit for a log[Inhibitor] *vs.* normalized answer model with variable slope.

#### β-Gal

Similar to the method applied in β-Glc assay, the hydrolysis of *o*-nitrophenyl-β-*O*-d-galactopyranoside (β-ONPG) was continuously measured in 96-well microplate. Wells were filled in triplicate with β-Gal (*Kluyveromyces lactis*) in 50 mM buffer phosphate, pH 7 (7.20 μU mL^−1^ final concentration in well) and 10 μL of test compound in dimethylsulfoxide (DMSO) solution (100 μM or 500 μM end concentration in well). The final volume in well was 270 μL. The enzymatic reaction was initiated by addition of β-ONPG (3.23 mM end concentration in well). The plate was shaken for 2 s and the increase in absorbance at 405 nm was monitored at 37 °C for 10 min. For IC_50_ determination, eighteen serial dilutions of the compounds were prepared in DMSO, following equally spaced points in neperian logarithm scale, starting at 94.50 mM and finishing at 1.44 μM. IC_50_ was calculated using Prism V5.01 (GraphPad Software Inc., La Jolla, CA, USA) requesting the software a non-linear regression curve fit for a log[Inhibitor] *vs.* normalized answer model with variable slope.

### Jump dilution assay

A DMSO solution of compound I-c was incubated at 10-fold its IC_50_, with the enzyme solution at 100-fold over the concentration used in the microplate assay. After 30 minutes of incubation, the mixture was diluted 100-fold and mixed with the substrate and α-CD to start the reaction. The progress curve of this sample was measured and compared to a sample of enzyme incubated and diluted in the absence of inhibitor. Each incubation and dilution were prepared and measured in triplicate. The percentage of activity recovery was obtained using Prism V5.01 (GraphPad Software Inc.).

### Kinetic experiments

Microplate assay was carried on varying substrate concentration between 0.022 and 7.2 mM; as well as the inhibitor concentration was varied between 0.239 and 0.0061 mM. Initial velocities *vs.* [S] curves were obtained with Michaelis–Menten equation adjustment. After that, Lineweaver–Burk linearization plot and the secondary linearization of Slopes *vs.* [I] reveled type of inhibition and *K*_i_ value respectively. All the data acquired was treated through Prism V5.01 (GraphPad Software Inc.).

## Conclusions

Chemical treatment of an herbal extract and bioguided fractionation lead to the identification of a new histamine derived sulfonamide as promising β-Glc inhibitor. This is the second bioactive compound generated in chemically engineered extracts through sulfonylation reaction. This new compound and its previously reported predecessor, isolated from the same CEE, inspired the creation of a small library of eighteen arylbenzenesulfonyl histamine derivatives. In general *N*^α^-arylbenzenesulfonyl histamine derivatives displayed better β-Glc inhibition properties than the *N*^α^,*N*^τ^-di-arylbenzenesulfonyl derivatives. Four library members, compounds I-c, I-d, I-g and I-h, showed β-Glc inhibition comparable to the reference inhibitor 1-DNJ. Three of them possess a fluorine atom in the *para*-position on the aromatic ring whereas the forth compound possesses a trifluormethyl substituent in that position. The best inhibitor I-c, is a reversible and competitive inhibitor with a *K*_i_ value that is almost six times lower than 1-DNJ (reference compound). In addition, I-c shows an interesting selectivity for β-Glc, it did not inhibit other glycosidases such as α-Glc and β-Gal in the range of concentration tested. Although these three glycoside hydrolases represent a small proportion of a big enzyme family, the observed selectivity is an interesting starting point for further studies with glycosidases related to lysosomal diseases.^47^ This finding illustrates how the directed chemical transformation of complex natural mixtures can lead to the discovery of unplanned enzyme inhibitors.

## Conflicts of interest

The authors have declared no conflicts of interest.

## Supplementary Material

RA-008-C8RA06625F-s001
