# Supplementary material for: N α-arylsulfonyl histamines as selective β-glucosidase inhibitors
Source: RSC Adv. 2018 Oct 24;8(63):36209–18. doi: 10.1039/c8ra06625f (PMC9088825; doi:10.1039/c8ra06625f)
Supplement: RA-008-C8RA06625F-s001 [file RA-008-C8RA06625F-s001.pdf]

Electronic Supplementary Material (ESI) for RSC Advances.
This journal is © The Royal Society of Chemistry 2018

Supplementary Information for

N^{α}-arylsulfonyl histamines as powerful and selective β-glucosidase inhibitors

M. O. Salazar,† M. I. Osella,† I. A. Ramallo and R. L. E. Furlan*

Farmacognosia, Departamento de Química Orgánica, Facultad de Ciencias Bioquímicas y Farmacéuticas, Universidad Nacional de Rosario, Suipacha 531, Rosario S2002LRK, Argentina

*Corresponding author: E-Mail: E-Mail: rfurlan@fbioyf.unr.edu.ar (R. L. E. Furlan).

† These authors contributed equally to this work

Contents

Figure S1 .... S2
Figure S1 .... S3
Mass spectrum of compounds Ia-Ij S4
Mass spectrum of compounds IIa-IIj S9
^{1}H NMR(300 MHz) of compounds Ia-Ij S14
^{1}H NMR(300 MHz) of compounds IIa-IIj S18
^{13}C NMR(300 MHz) of compounds Ia-Ij S22
^{13}C NMR(300 MHz) of compounds IIa-IIj S26
^{19}F NMR(300 MHz) of compounds Ic-Ij S30
^{19}F NMR(300 MHz) of compounds IIc-IIj S33
Plots of % of inhibition of β-glucosidase versus log [I] of compounds Ia-Ij S36

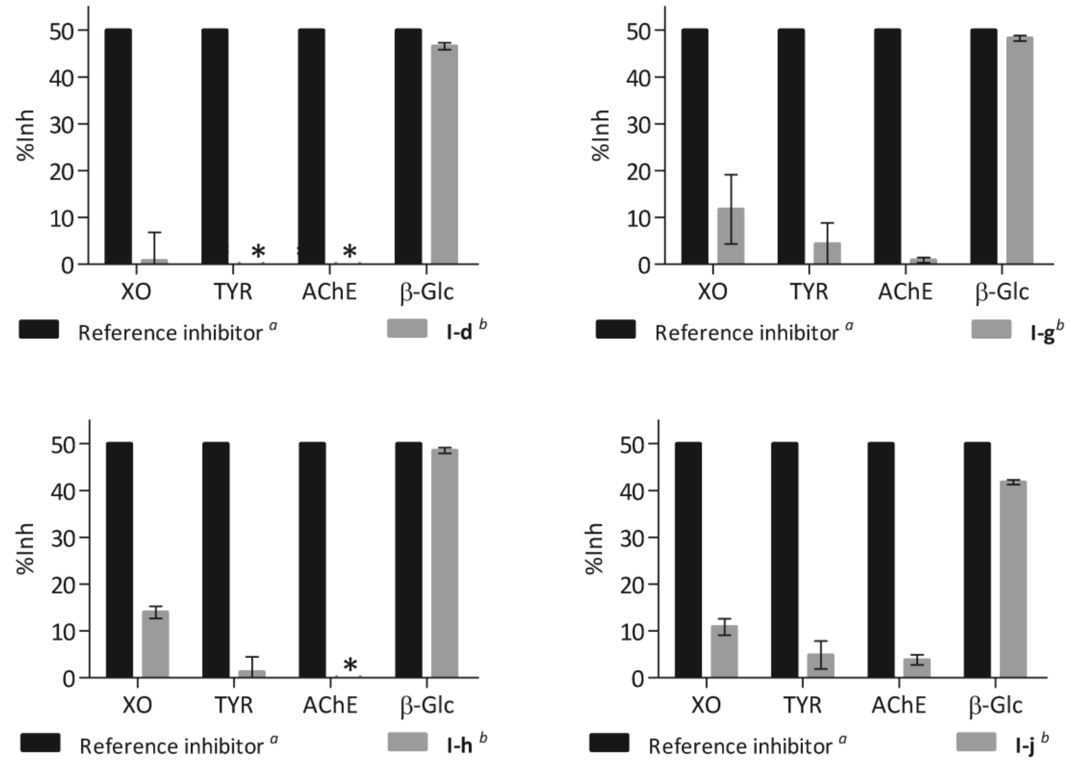

Fig, S1. Comparison of the inhibitory potency of compounds **I-d**, **I-g**, **I-h**, and **I-j** *vs* the reference inhibitor for each enzyme tested. [a] allopurinol for XO (IC 50 2.52 μM), kojic acid for TYR (IC 50 40.00 μM), eserine for AChE (IC 50 1.17 μM) and 1-DNJ for βGlc (IC 50 65.18 μM). [b] Compounds **I-d**, **I-g**, **I-h**, and **I-j** were tested at 3 μM in XO assay, at 40 μM in TYR assay, 1.5 μM in AChE assay and 65 μM in β-Glc. *No inhibition was observed.

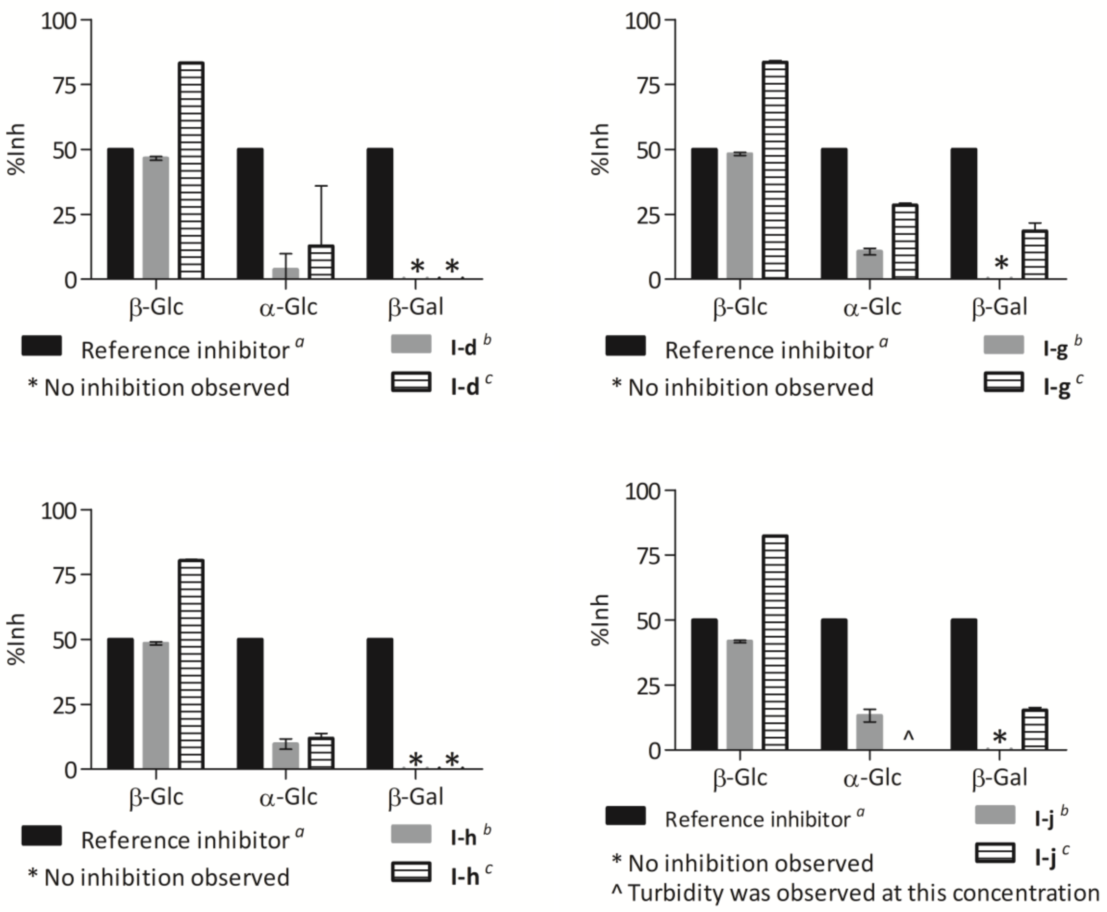

Fig, S2. Comparison of the inhibitory potency of compound compounds **I-d**, **I-g**, **I-h**, and **I-j** *vs* the reference inhibitor for each enzyme tested. [a] 1-DNJ for β-Glc (IC_{50} = 65.18 µM), 1-DNJ for α-Glc (IC_{50} = 333,00 µM) and *galacto*-DNJ for β-Gal (IC_{50} = 90 µM).[1] [b-c] Compouns **I-d**, **I-g**, **I-h**, and **I-j** were tested at, 65 and 600 µM in β-Glc, 383 and 2397 µM in α-Glc assay and 100 and 500 µM in β-Gal assay. *No inhibition was observed.

[1] A. Kato, N. Kato, E. Kano, I. Adachi, K. Ikeda, L. Yu, T. Okamoto, Y. Banba, H. Ouchi, H. Takahata, and N. Asano, J. Med. Chem., **2005,** *48,* 2036-2044

Mass spectrum

Compound Ia

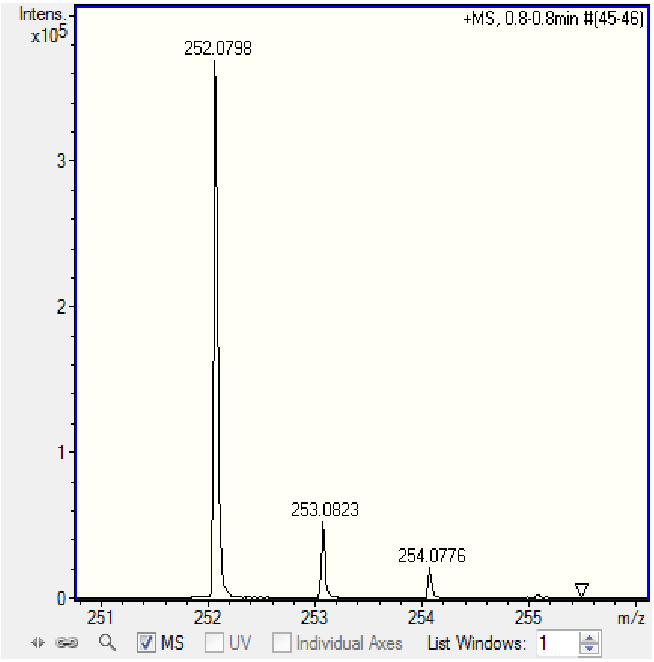

Compound Ib

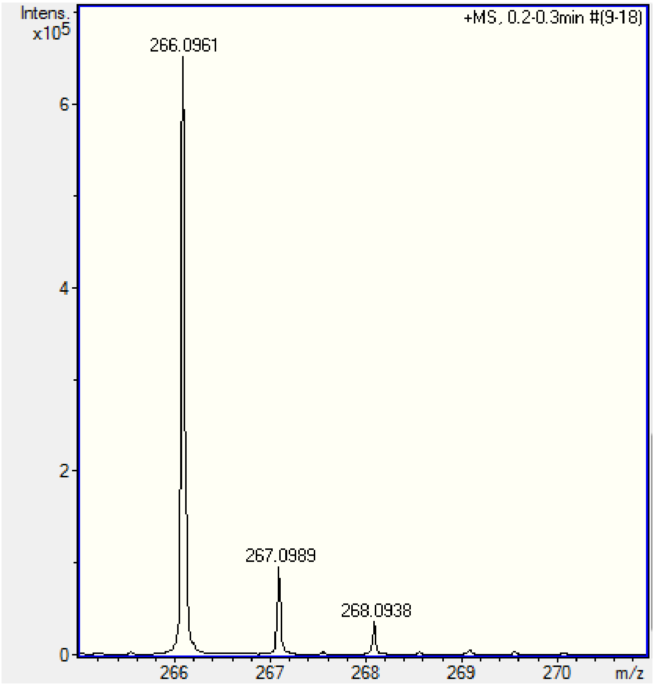

Compound Ic

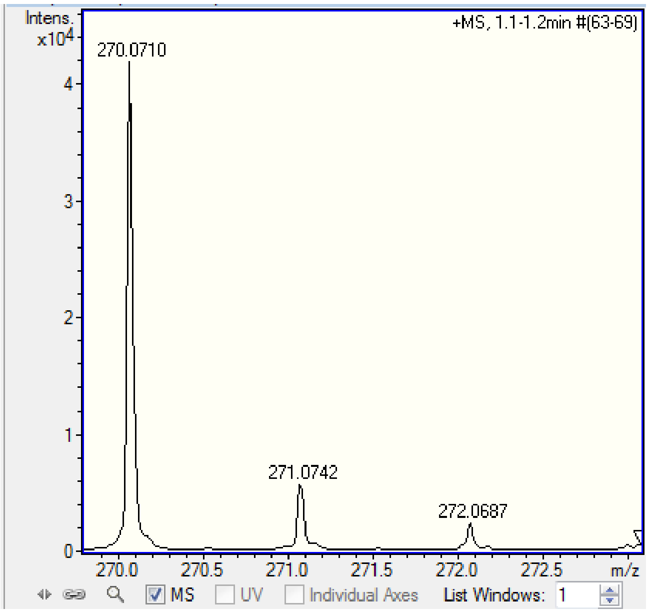

Compound Id

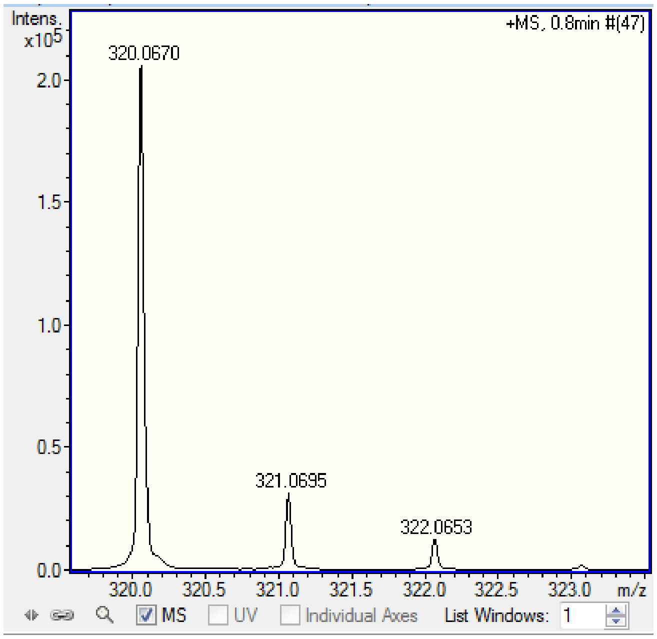

Compound Ie

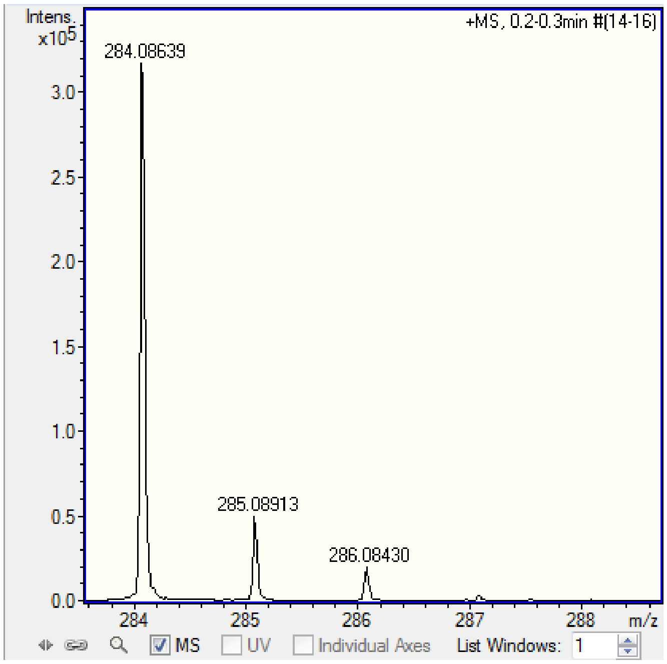

Compound If

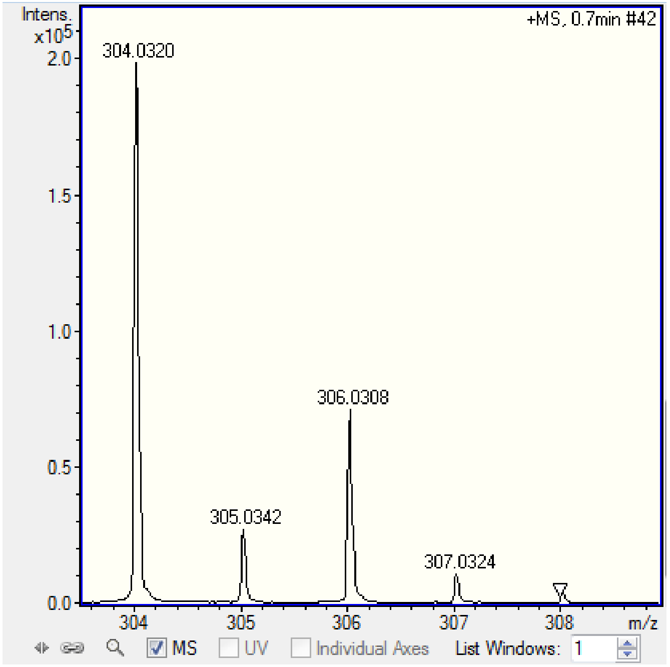

Compound Ig

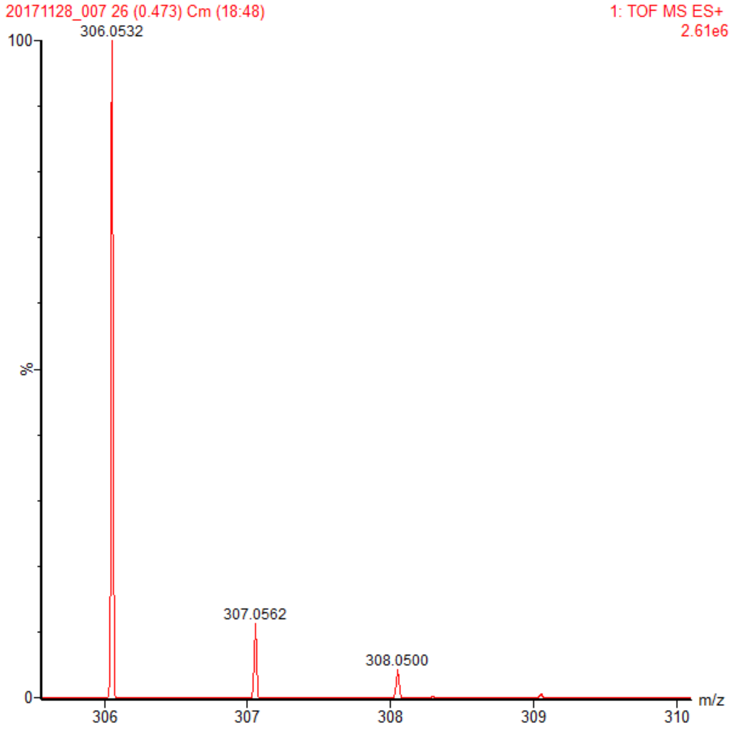

Compound Ih

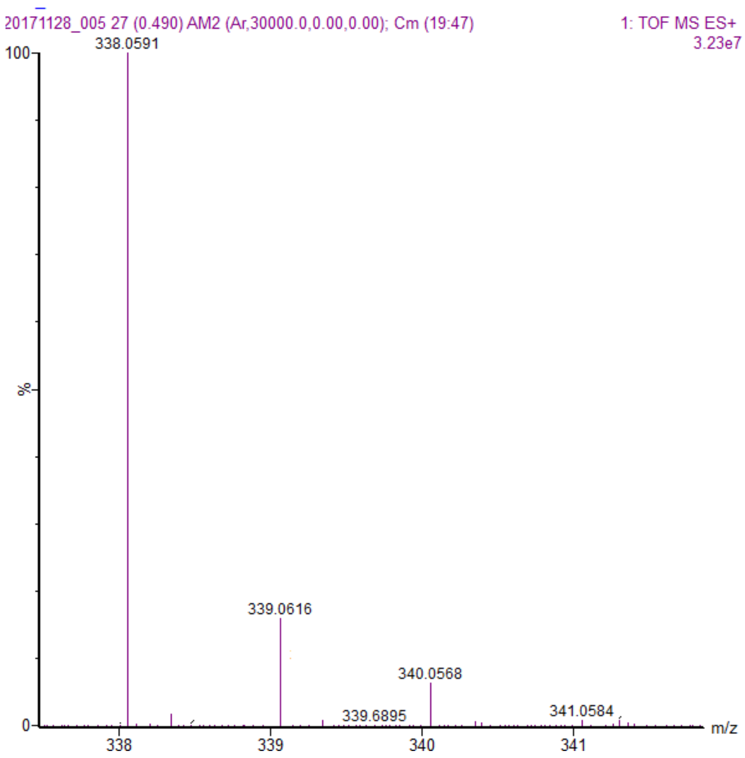

Compound Ii

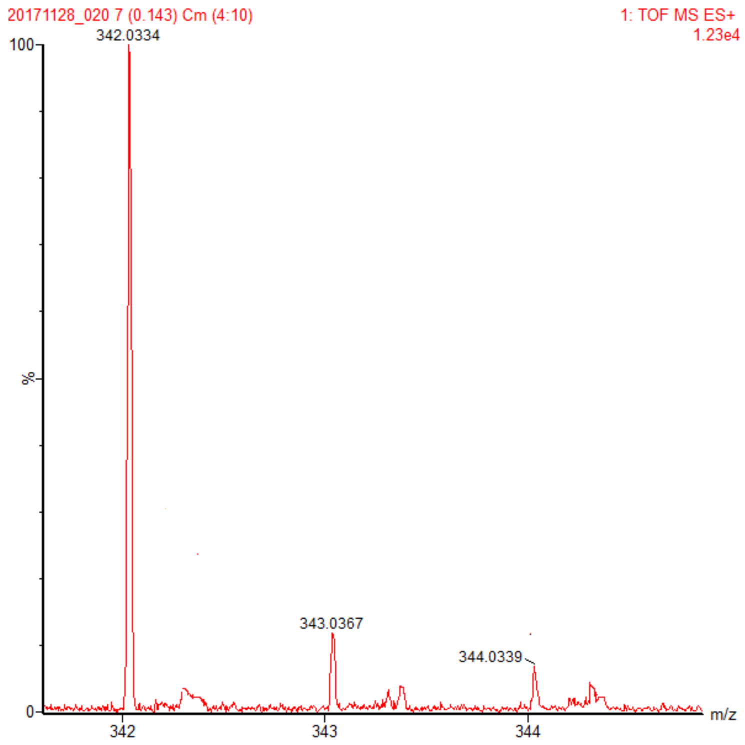

Compound Ij

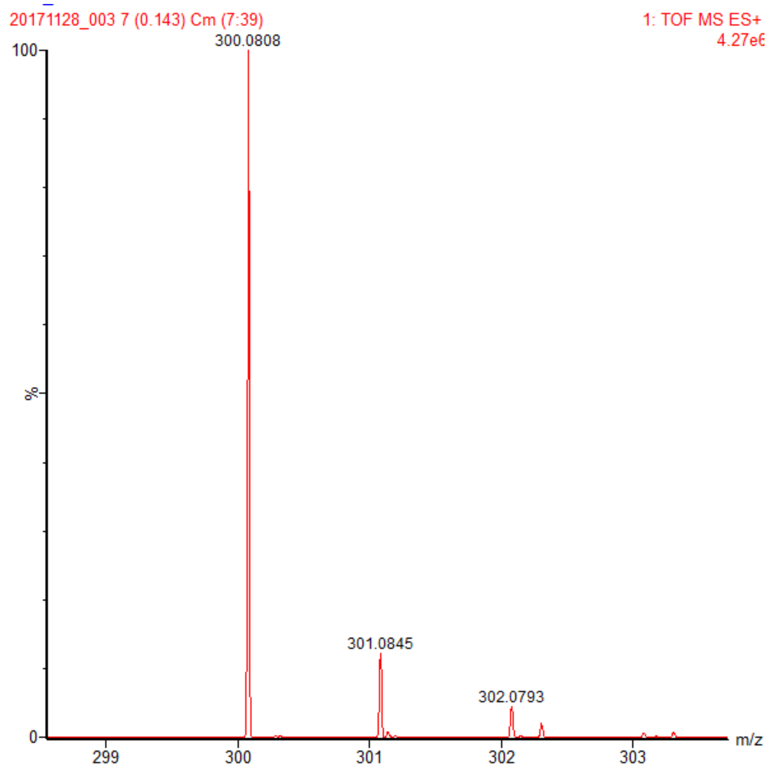

Compound IIa

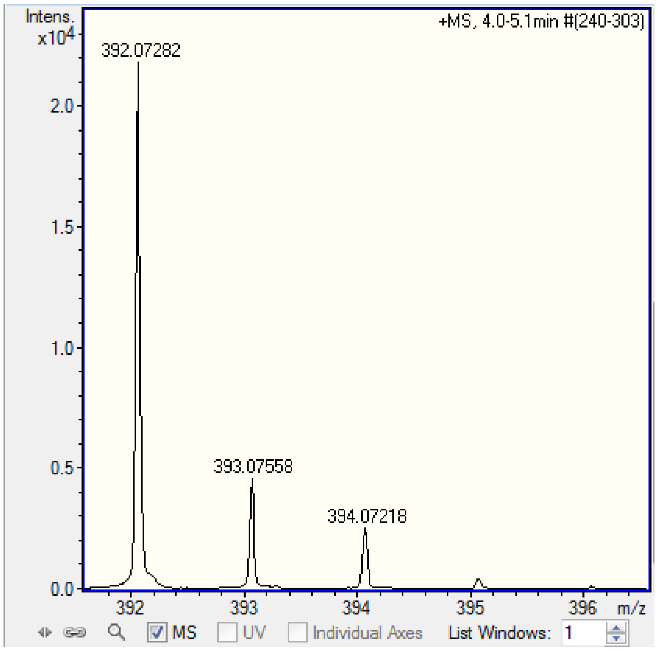

Compound IIb

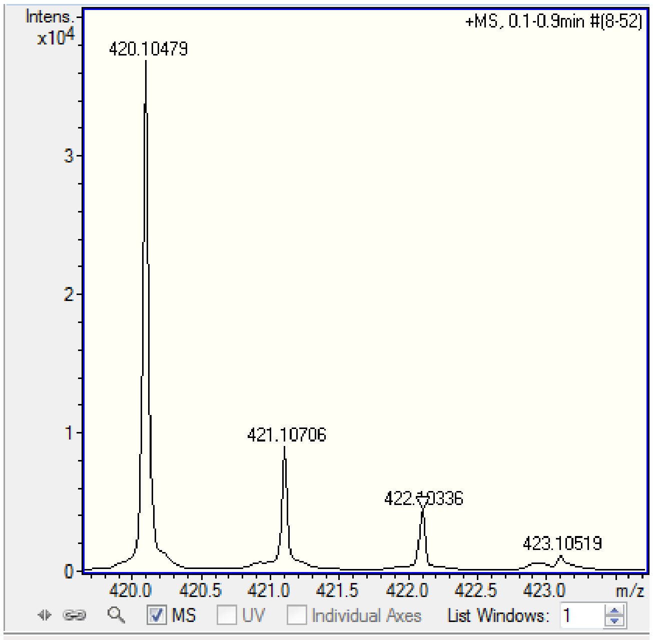

Compound IIc

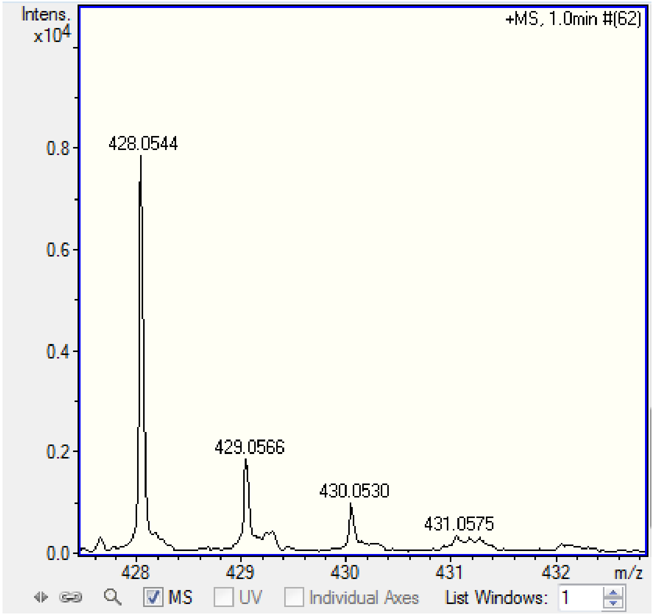

Compound IId

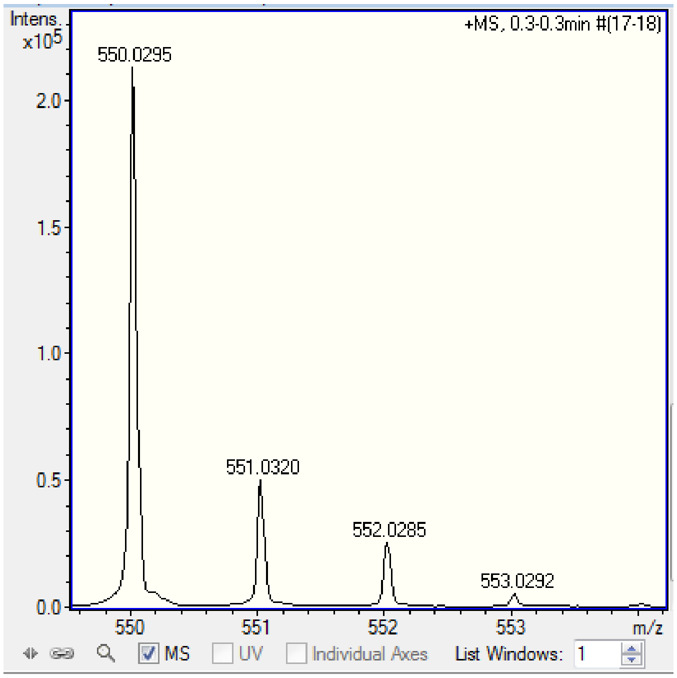

Compound IIe

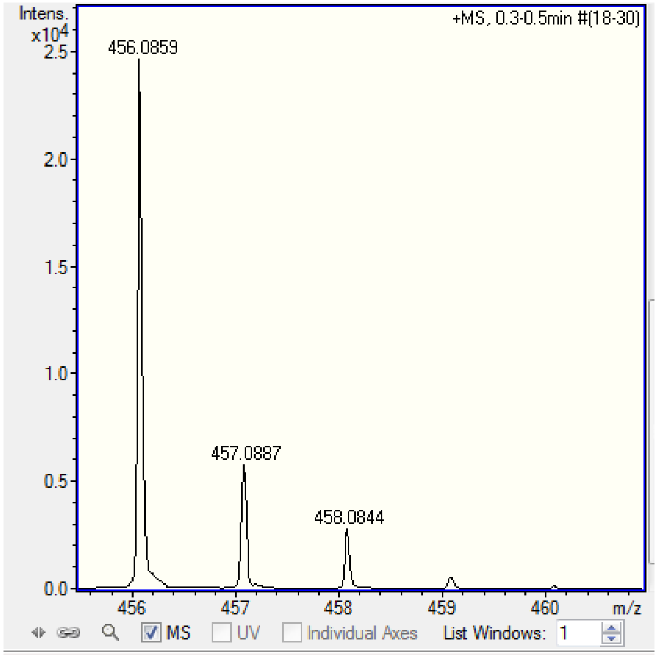

Compound IIf

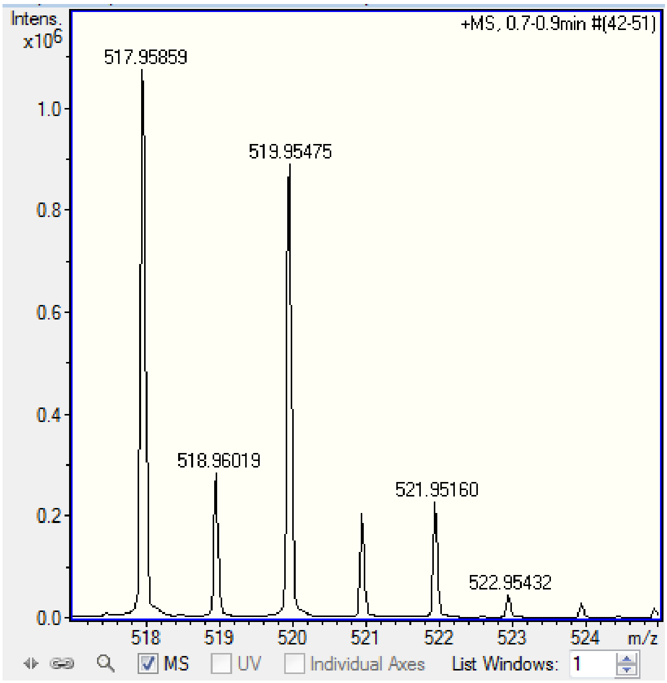

Compound IIg

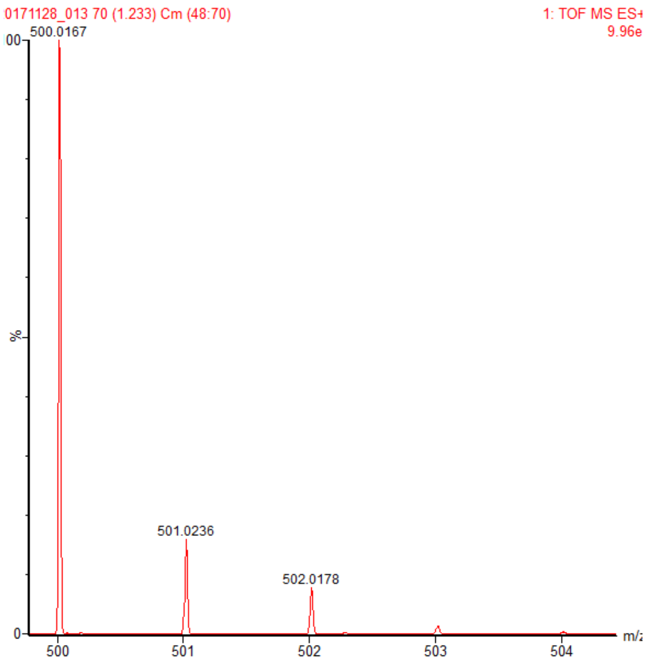

Compound IIh

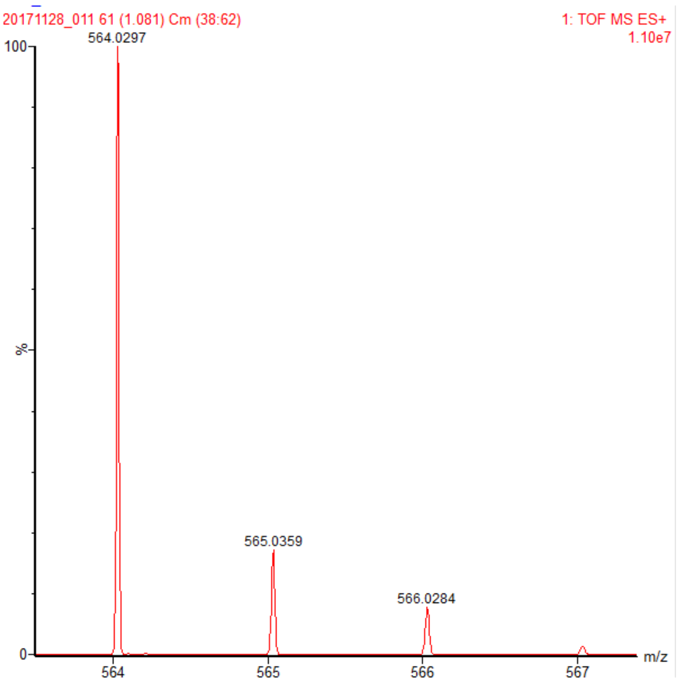

Compound IIi

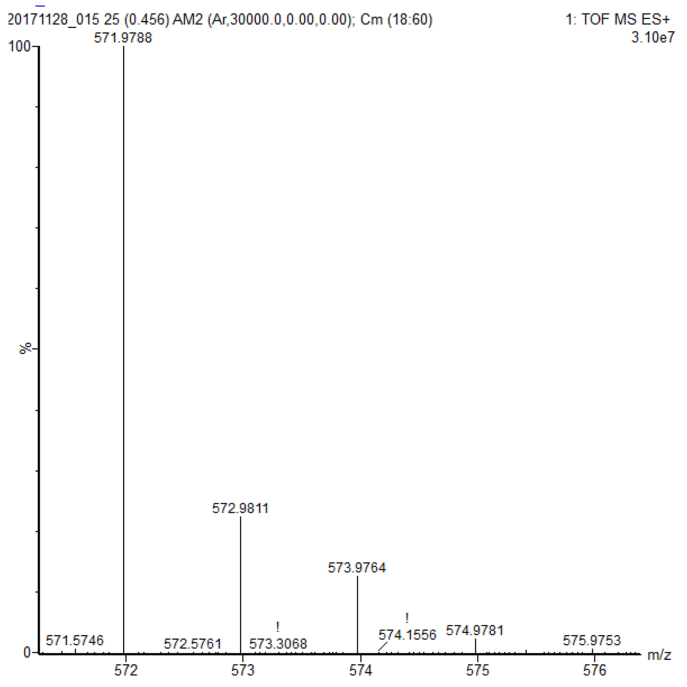

Compound IIj

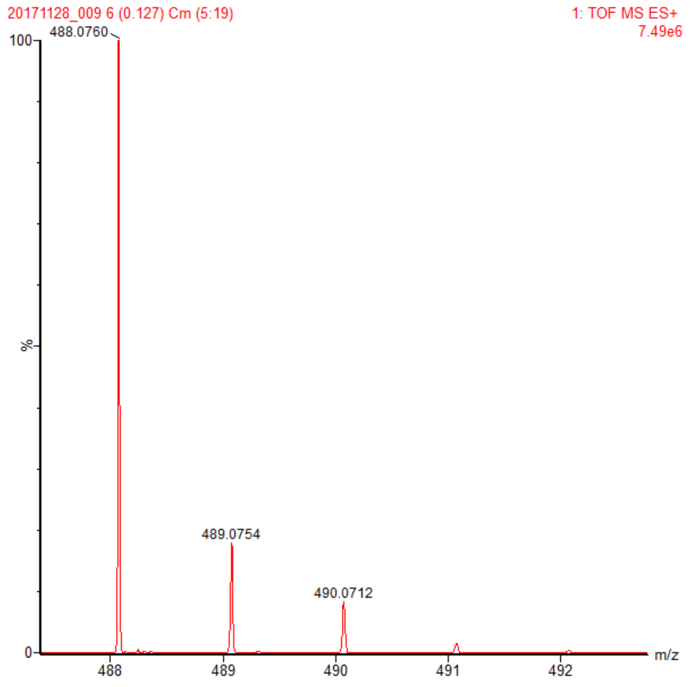

[1] H NMR(300 MHz) of compounds Ia-Ij..S50
Compound Ia

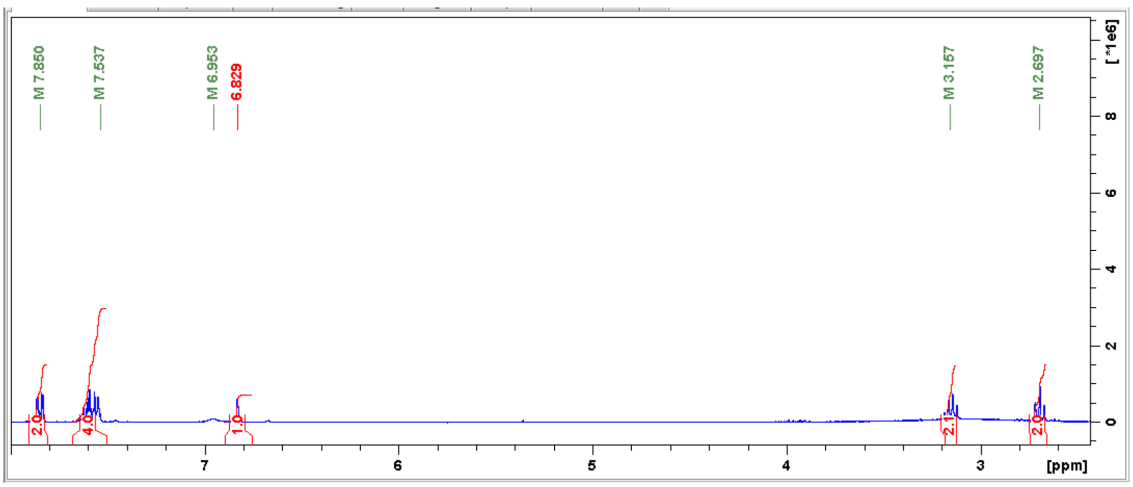

Compound Ib

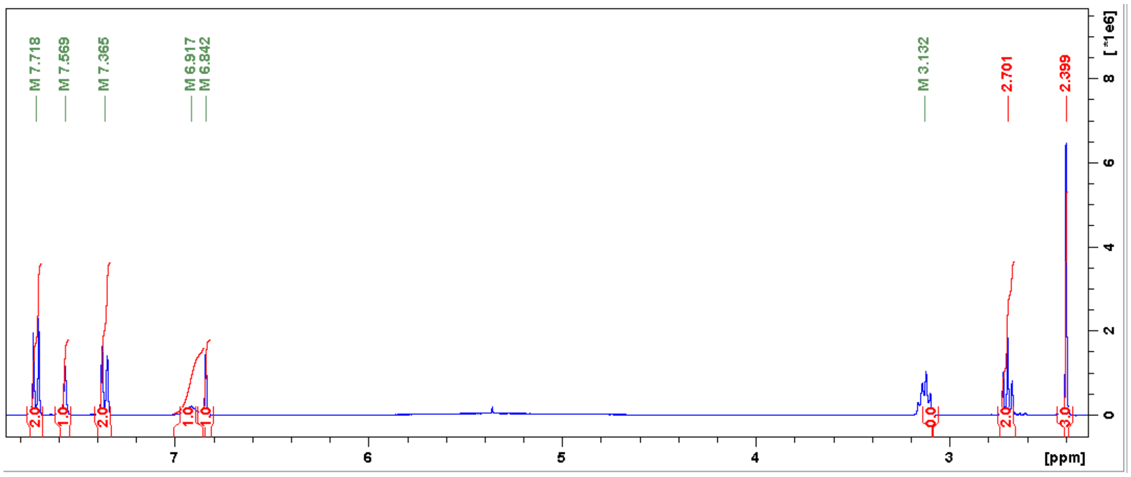

Compound Ic

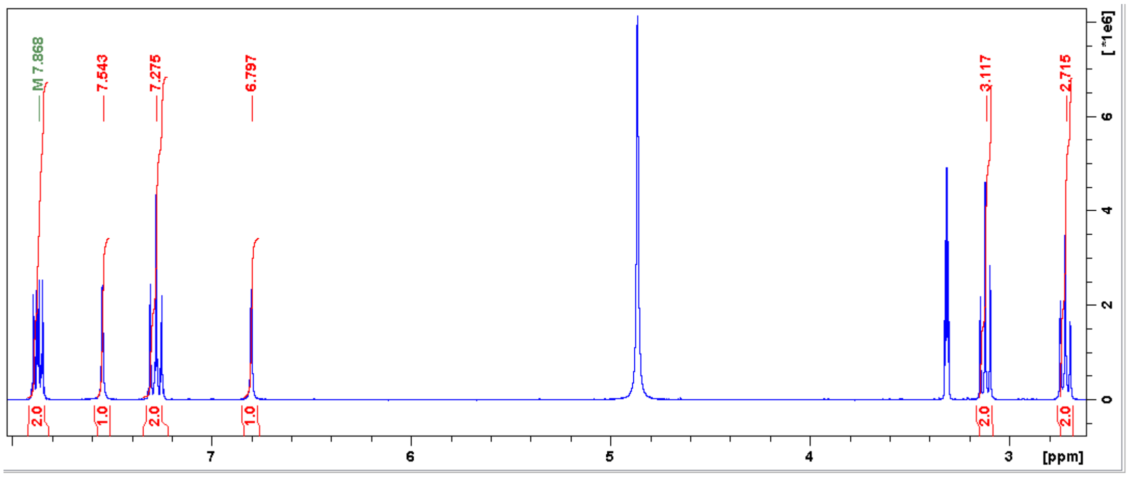

Compound Id

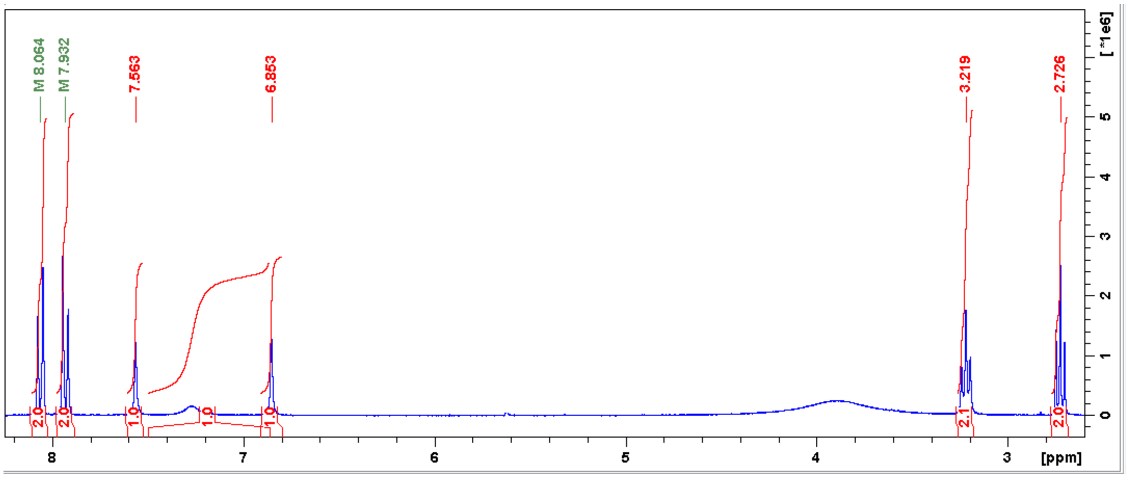

Compound Ie

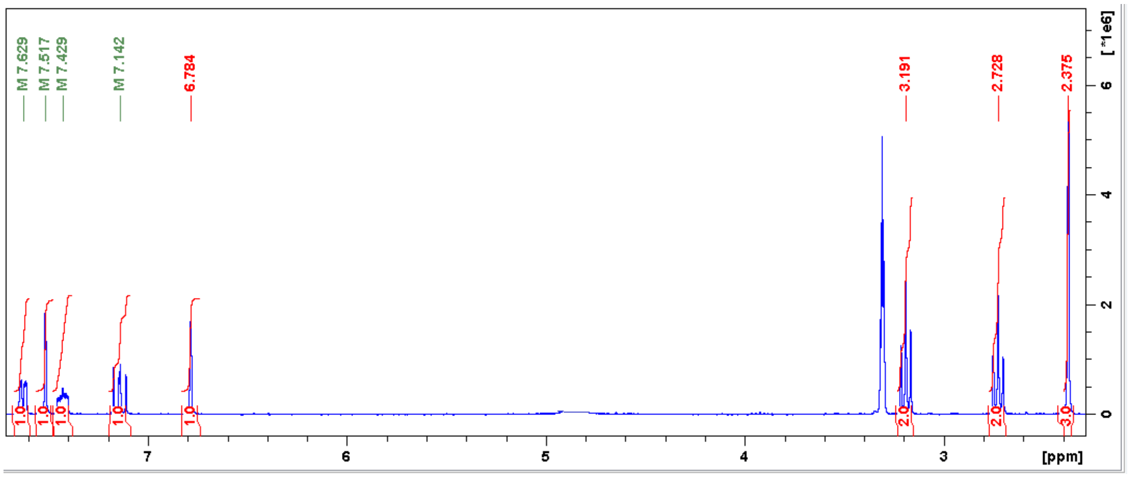

Compound If

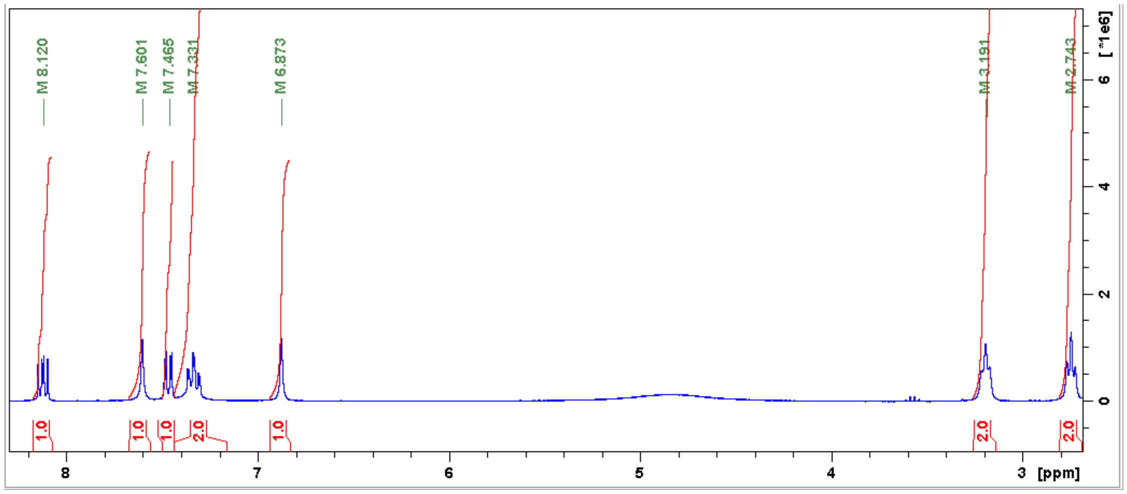

Compound Ig

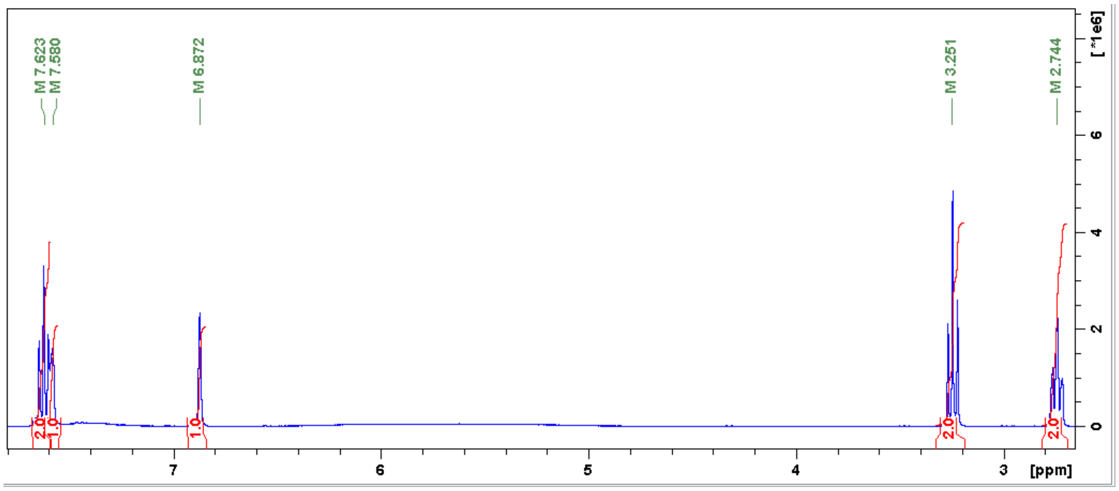

Compound Ih

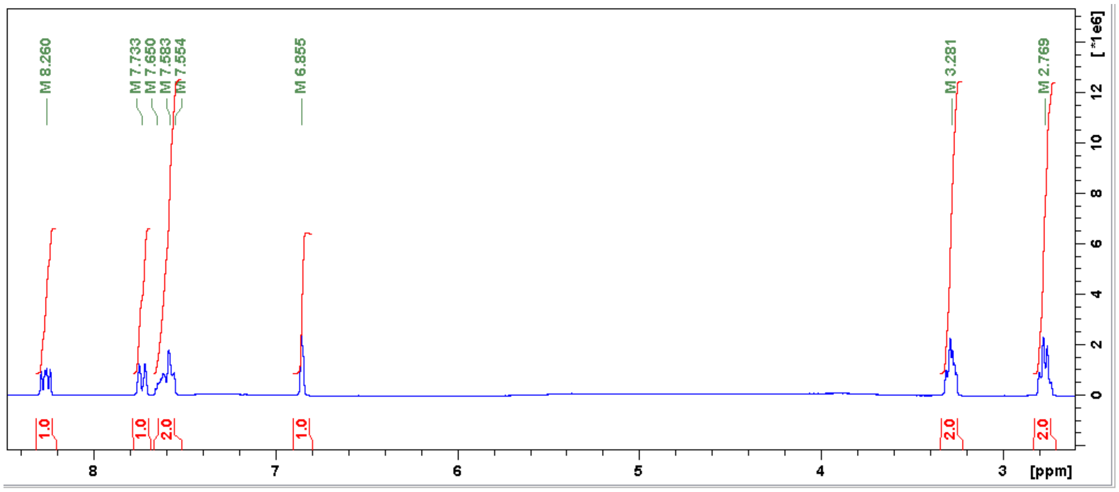

Compound Ii

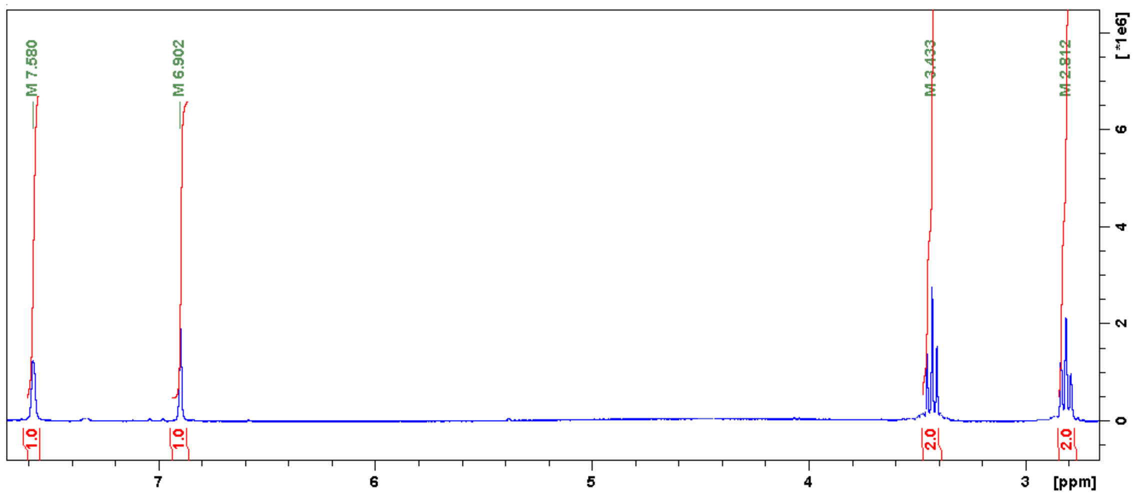

Compound Ij

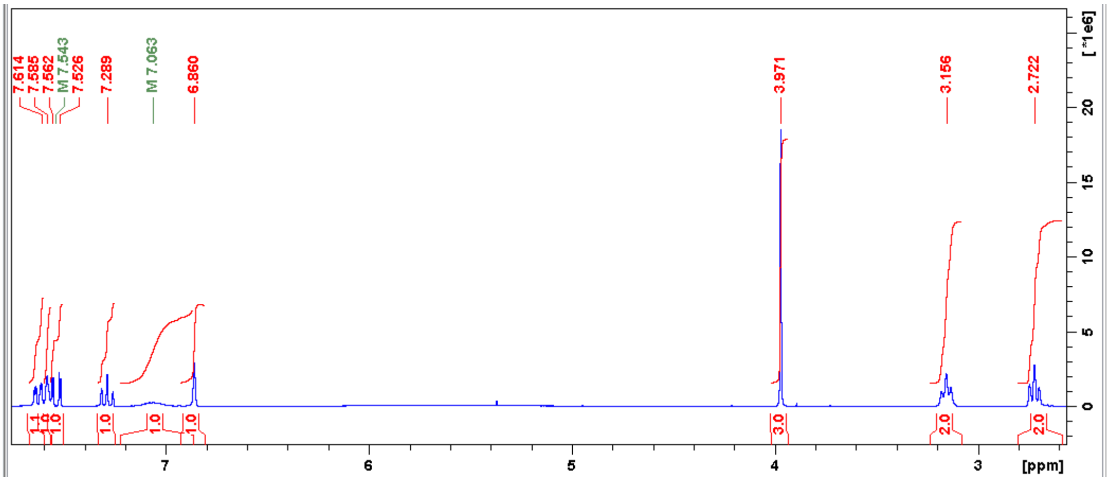

7.614
7.585
7.562
M 7.543
7.526
7.289
M 7.063
6.860
3.971
3.156
2.722
1.1
1.0
1.0
1.0
1.0
1.0
3.0
2.0
2.0
7
6
5
4
3
[ppm]
[*1e6]

^{1}H NMR(300 MHz) of compounds IIa-IIj

Compound IIa

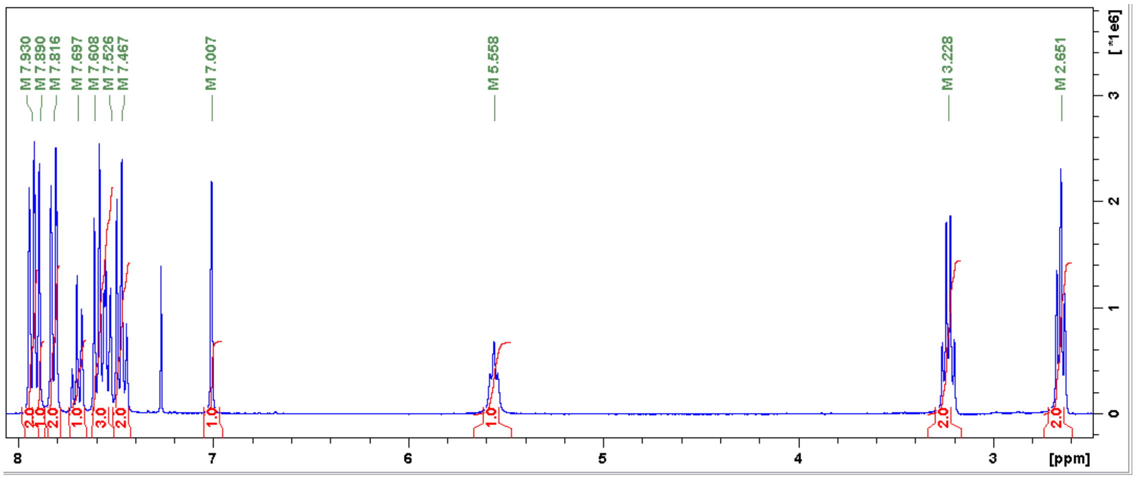

Compound IIb

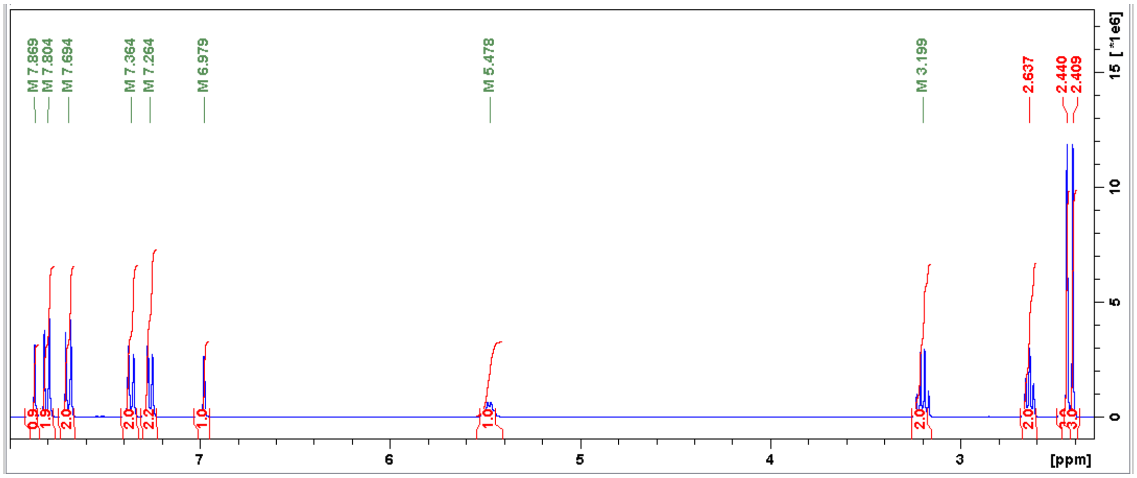

Compound IIc

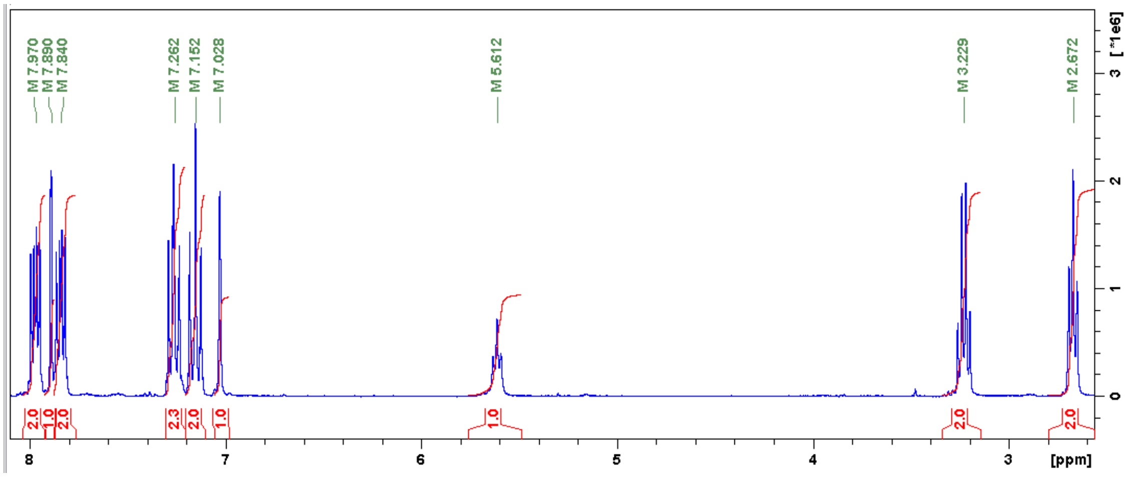

Compound IId

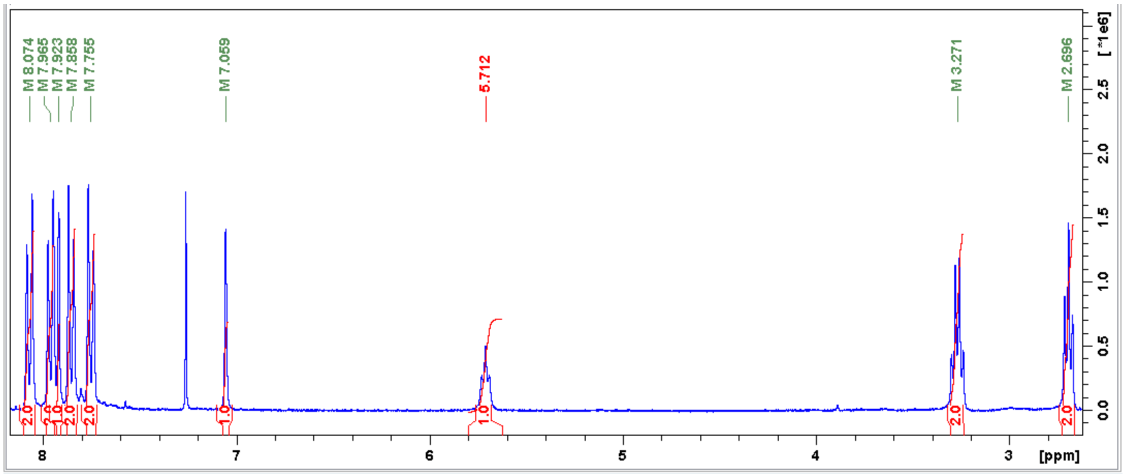

Compound IIe

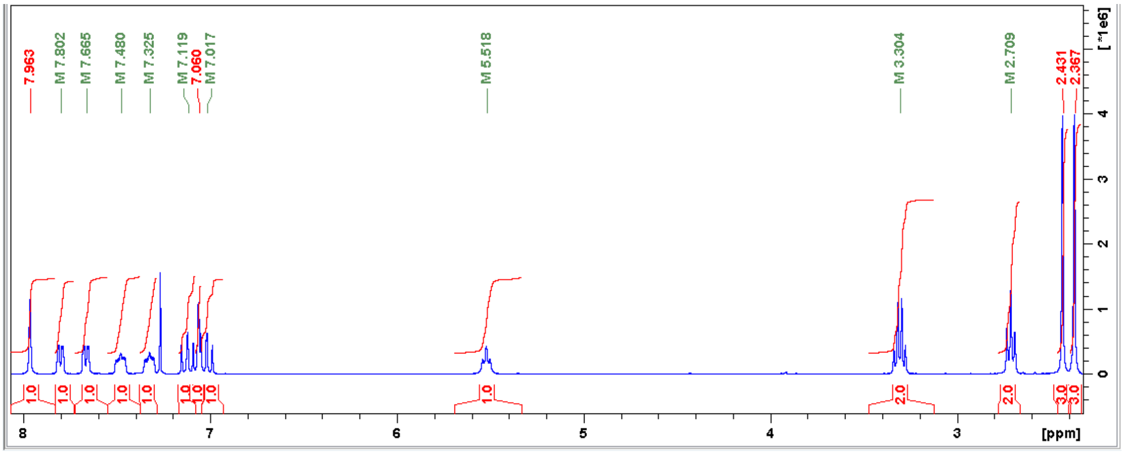

Compound IIf

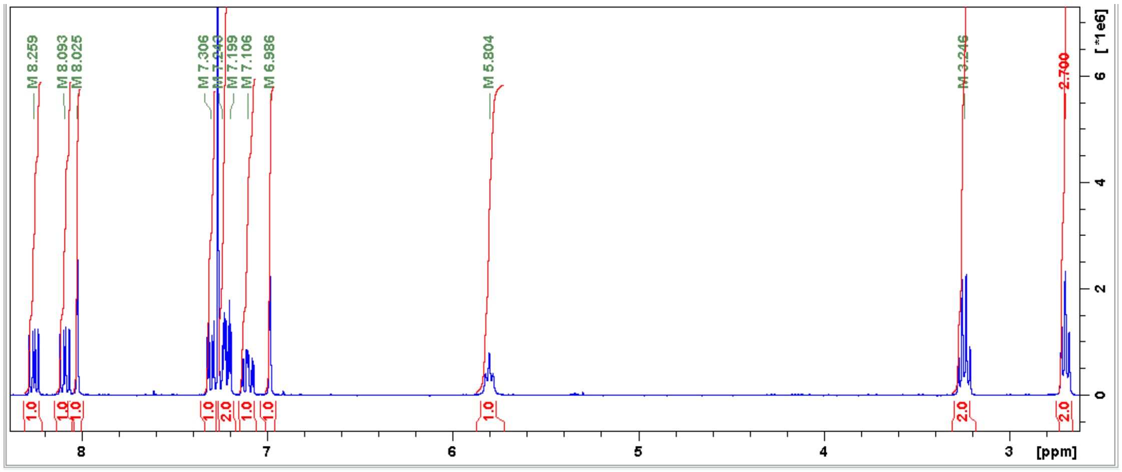

Compound IIg

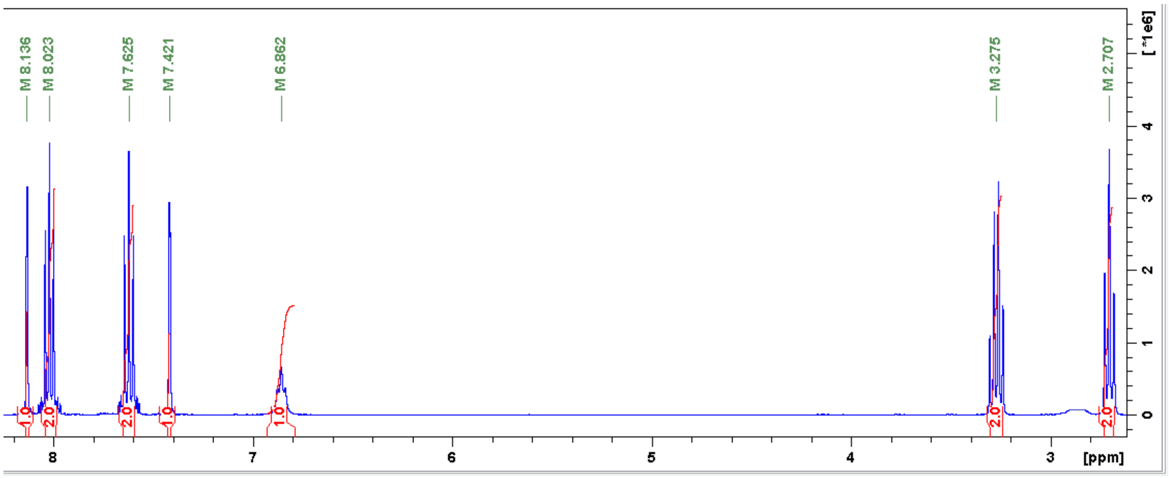

Compound IIh

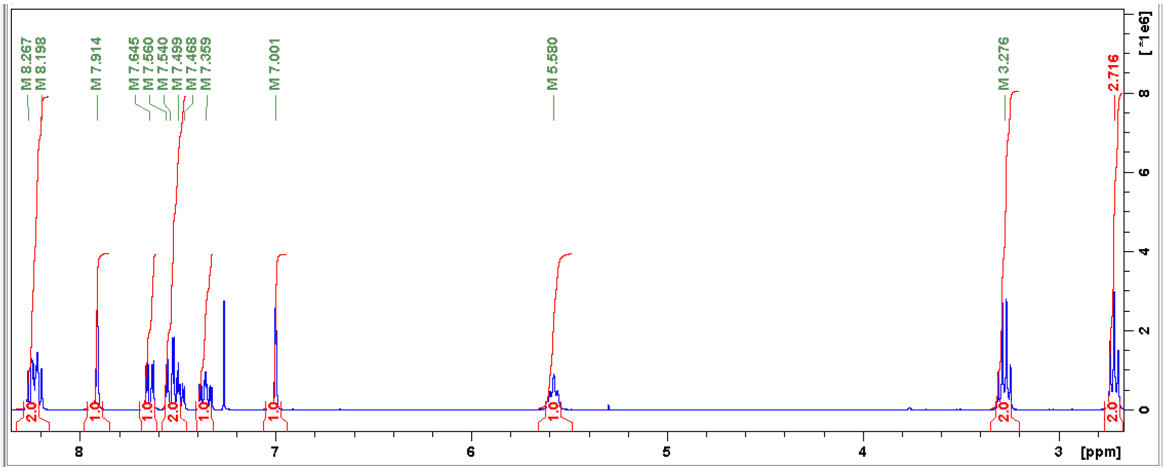

Compound IIi

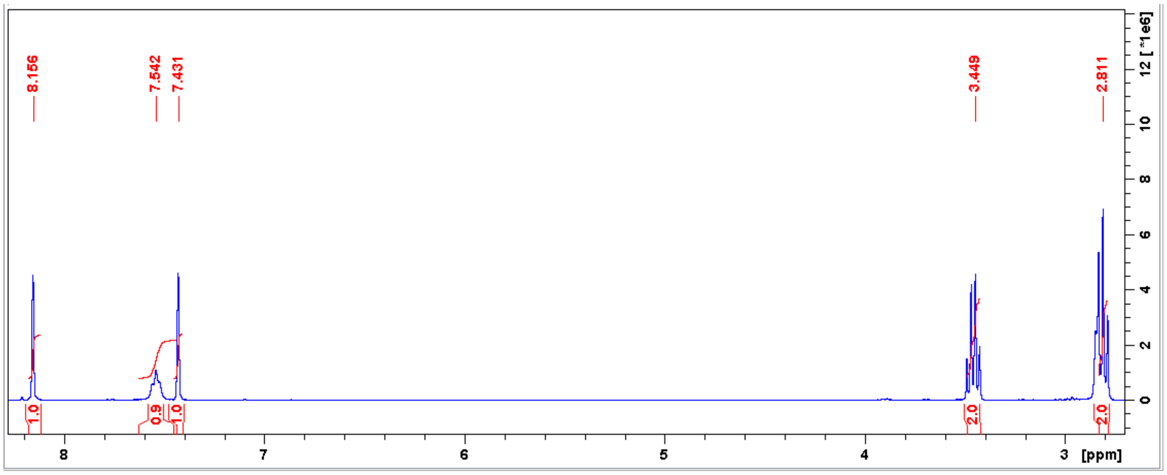

Compound IIj

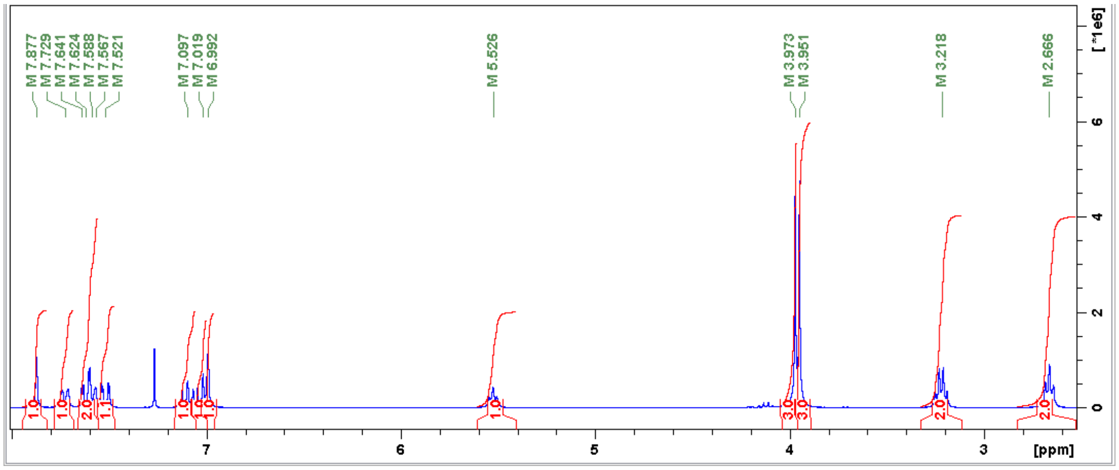

^{13}C NMR(300 MHz) of compounds Ia-Ij...S66

Compound Ia

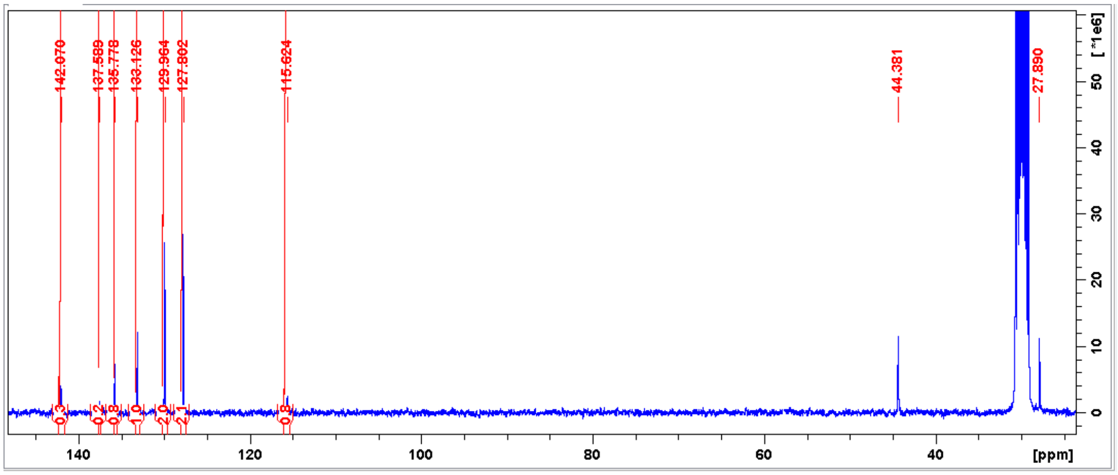

Compound Ib

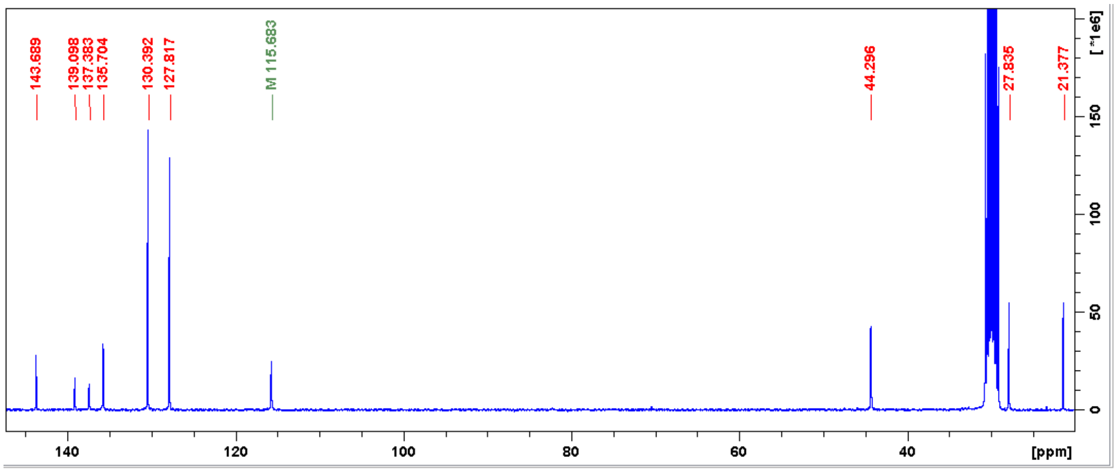

Compound Ic

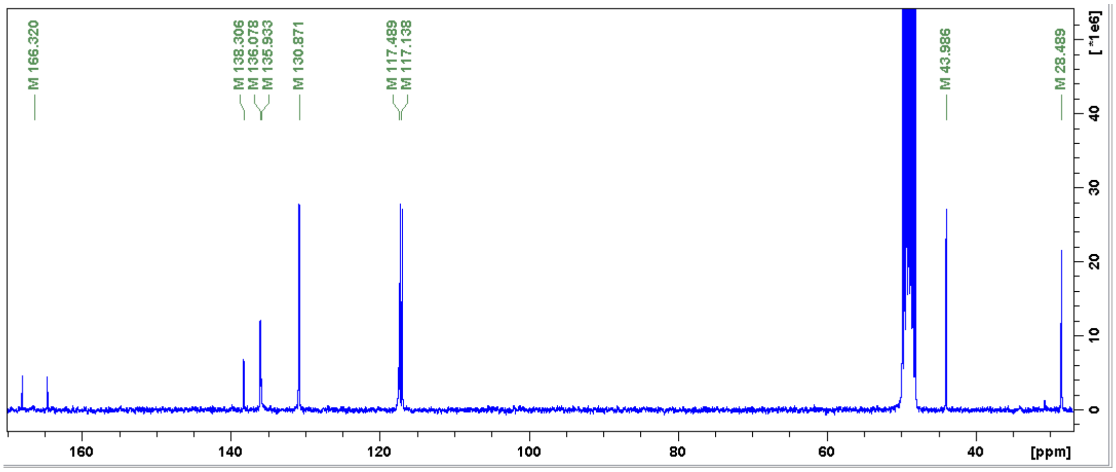

Compound Id

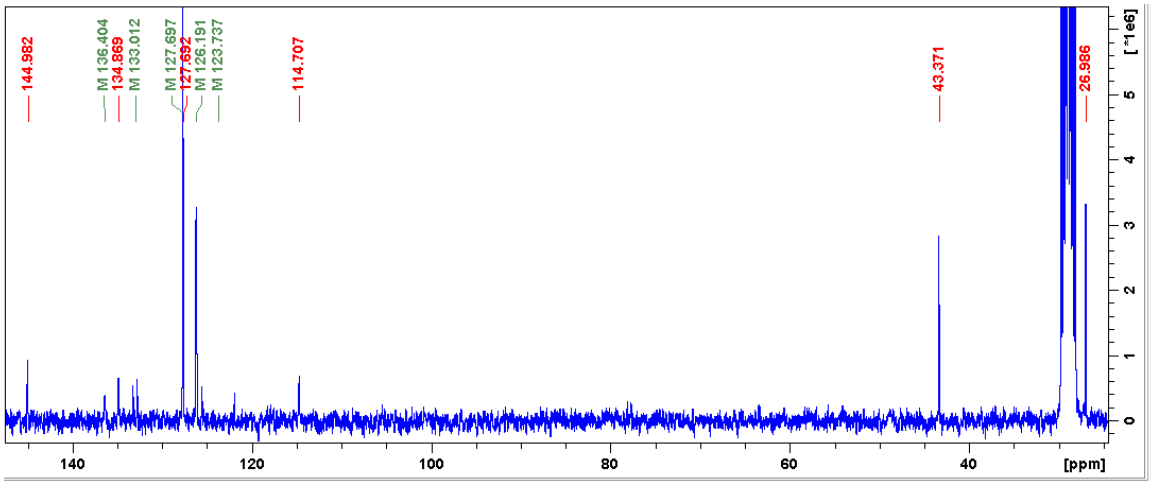

Compound Ie

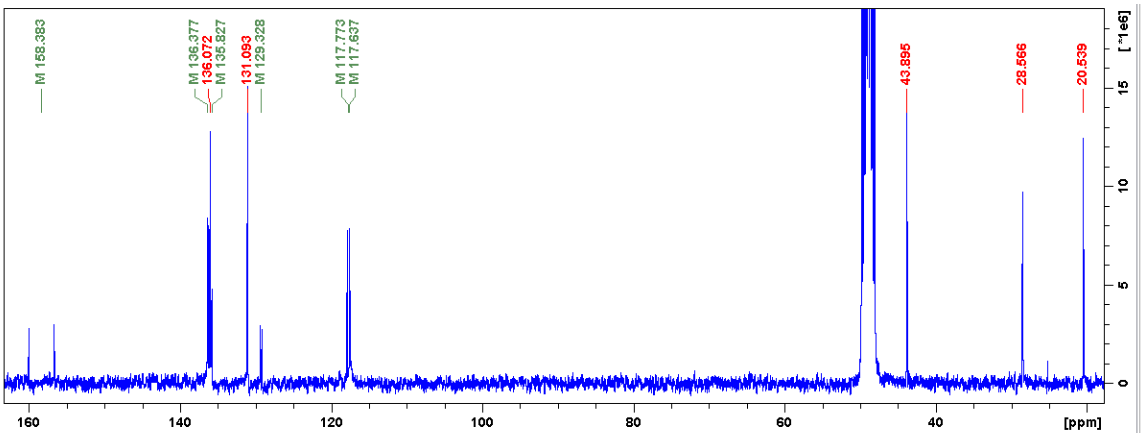

Compound If

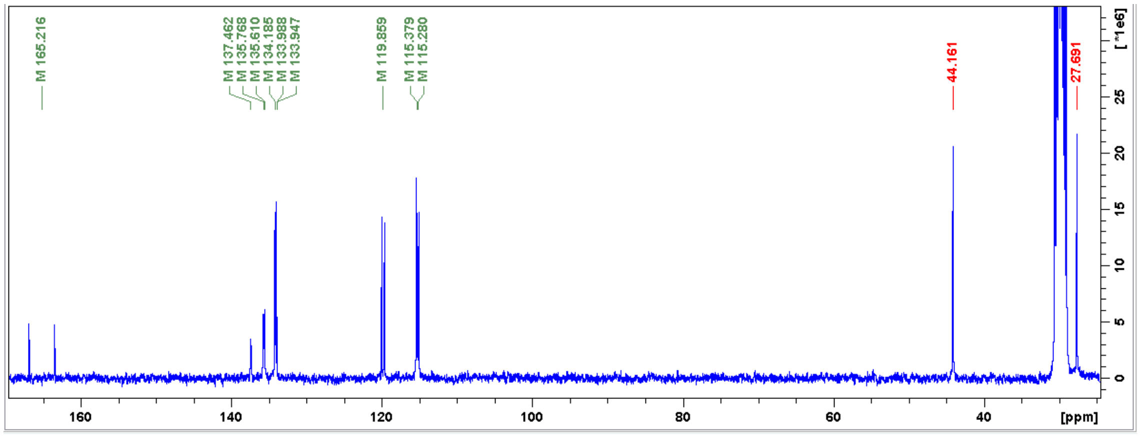

Compound Ig

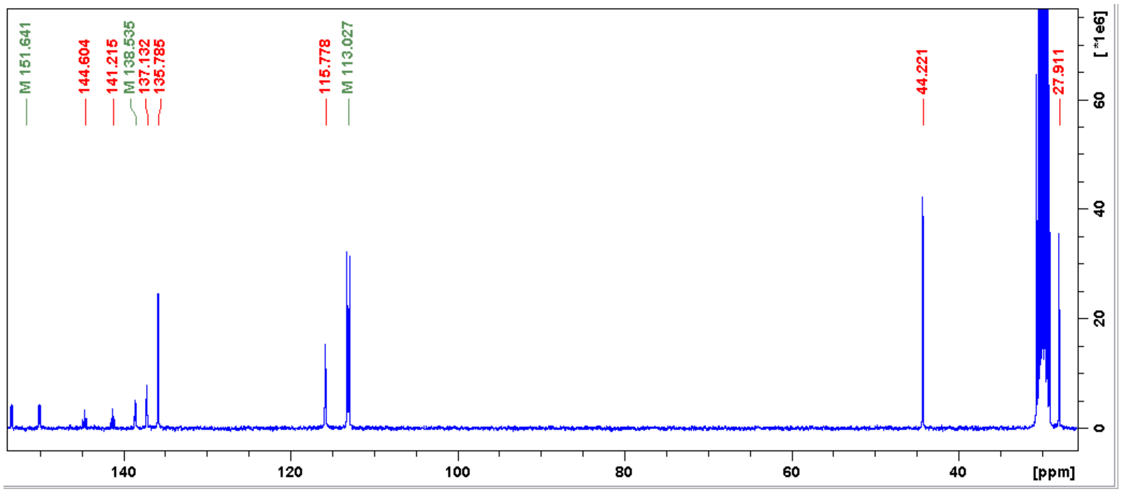

Compound Ih

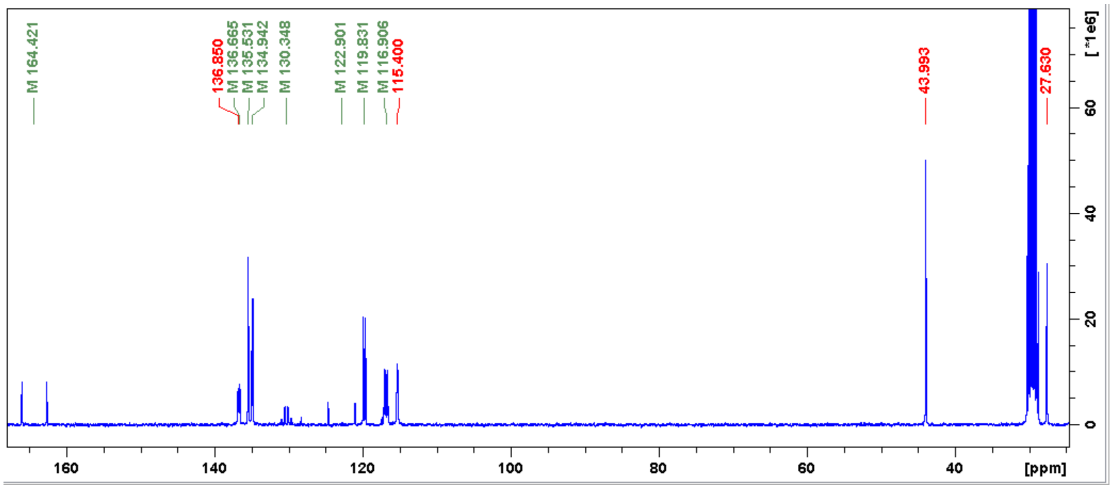

Compound Ii

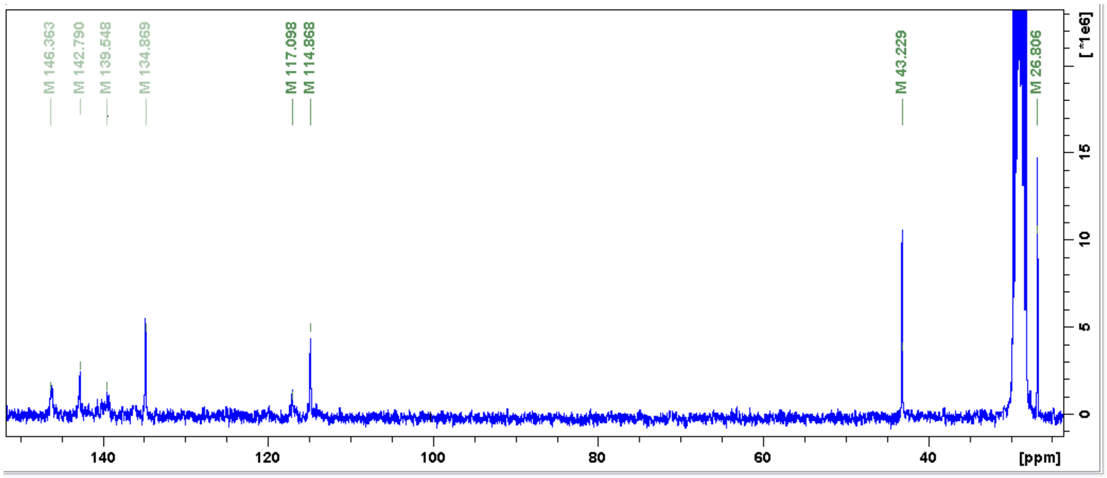

Compound Ij

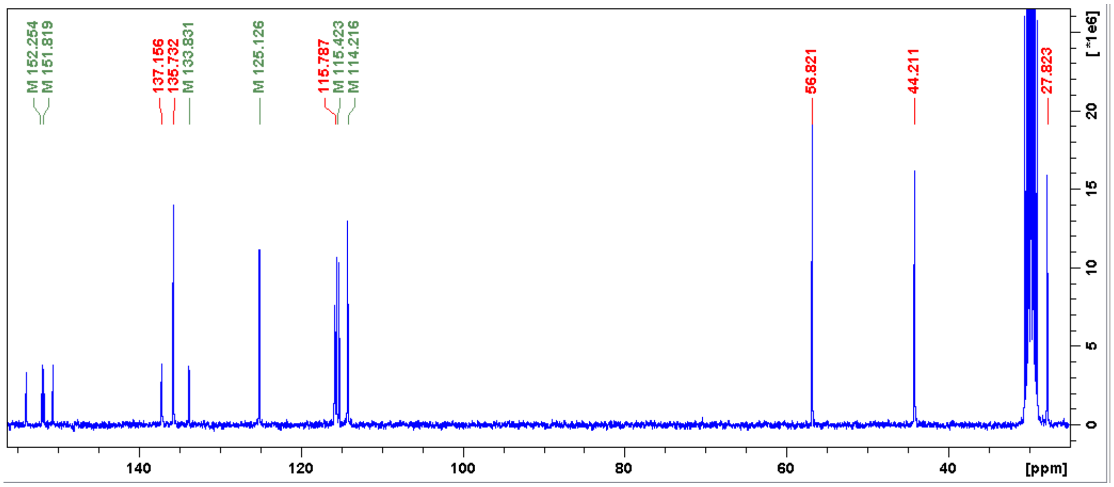

M 152.254
M 151.819
137.156
135.732
M 133.831
M 125.126
115.787
M 115.423
M 114.216
56.821
44.211
27.823
[*1e6]
20
15
10
5
0
140
120
100
80
60
40
[ppm]

^{13}C NMR(300 MHz) of compounds IIa-IIj

Compound IIa

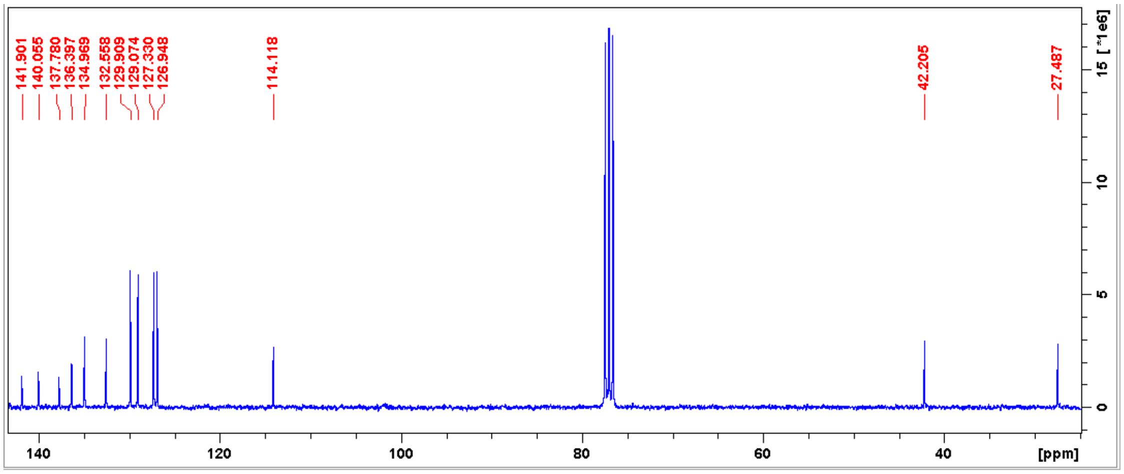

Compound IIb

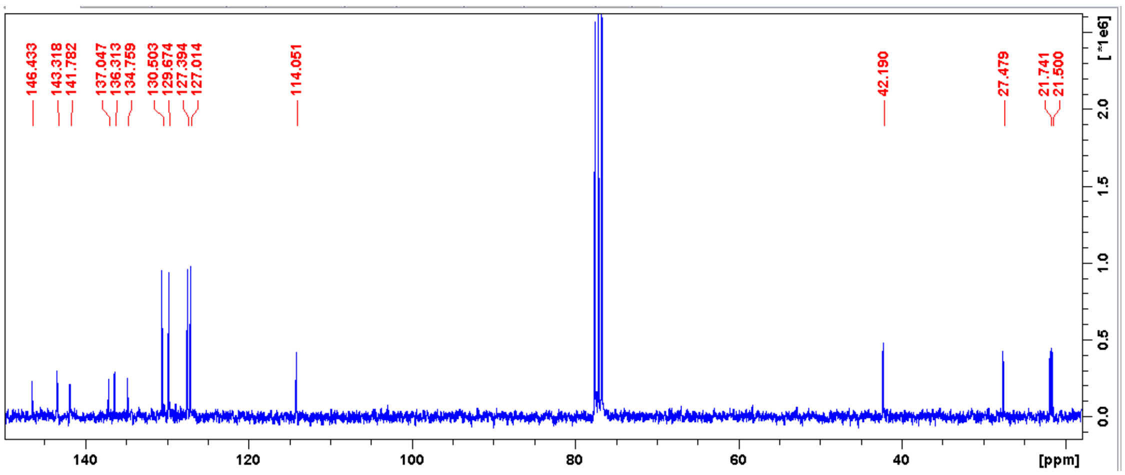

Compound IIc

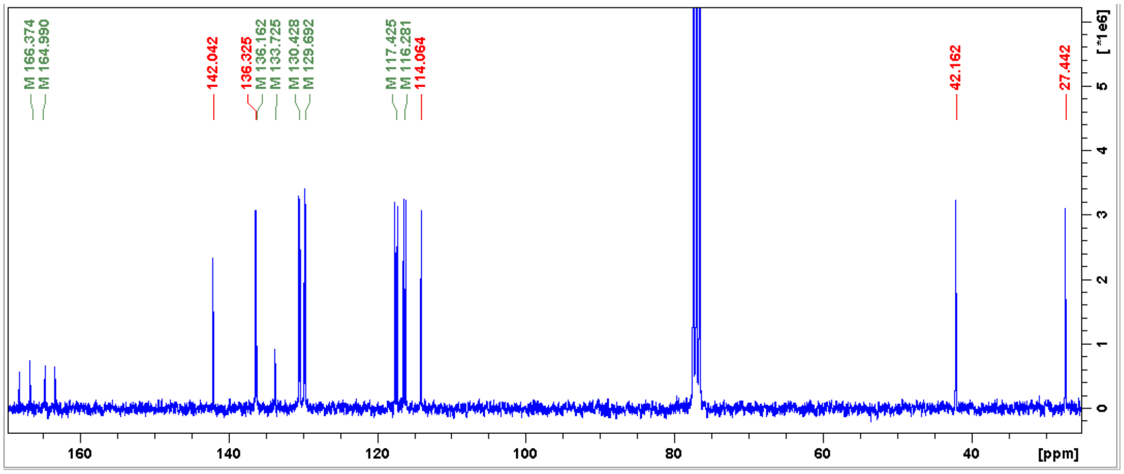

Compound IId

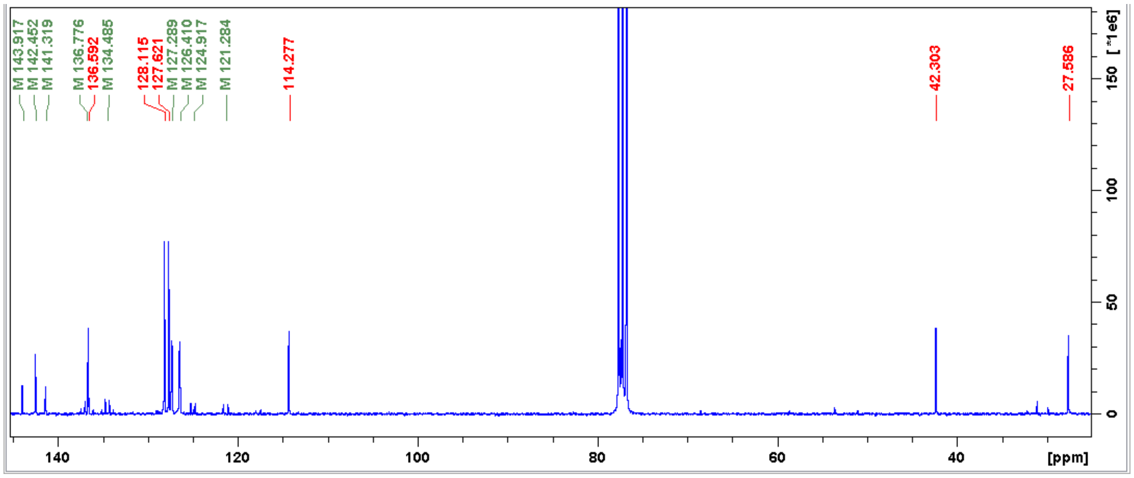

Compound IIe

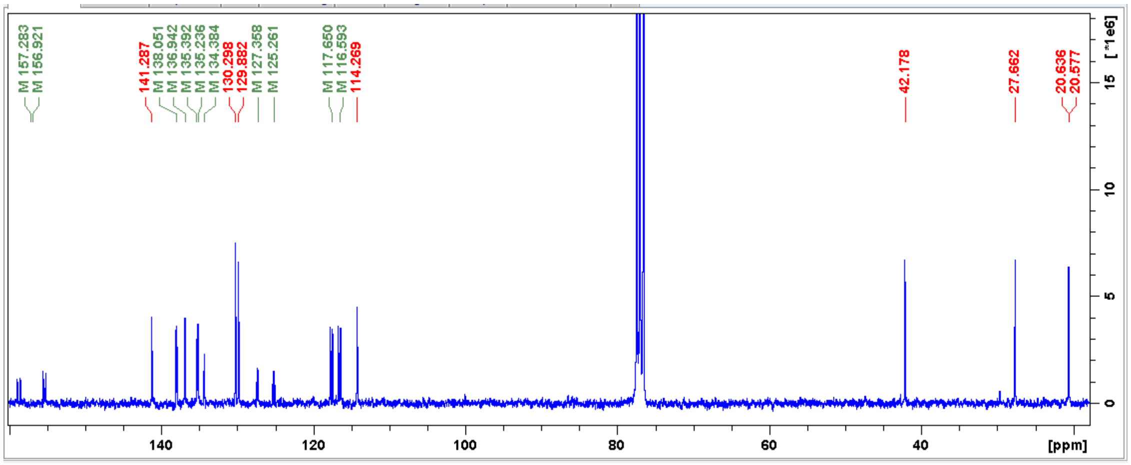

Compound IIf

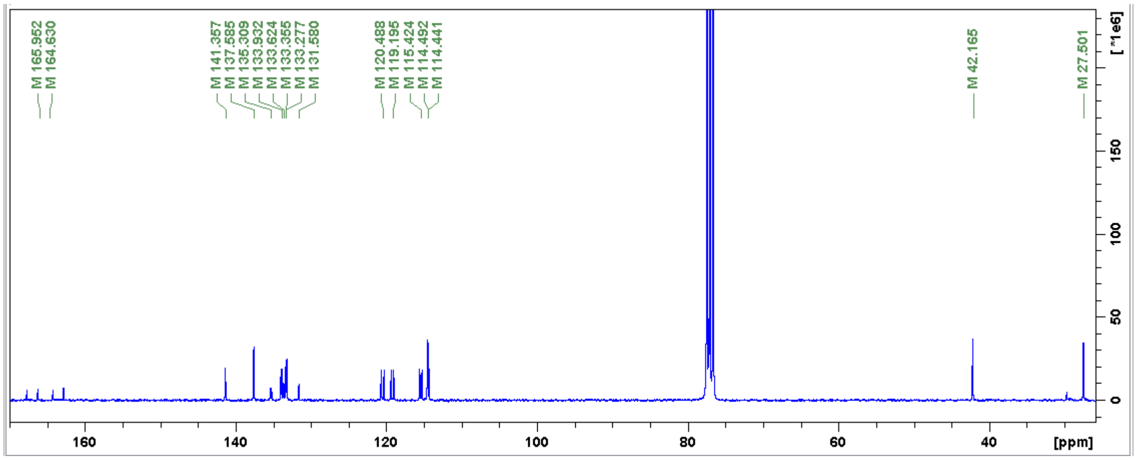

Compound IIg

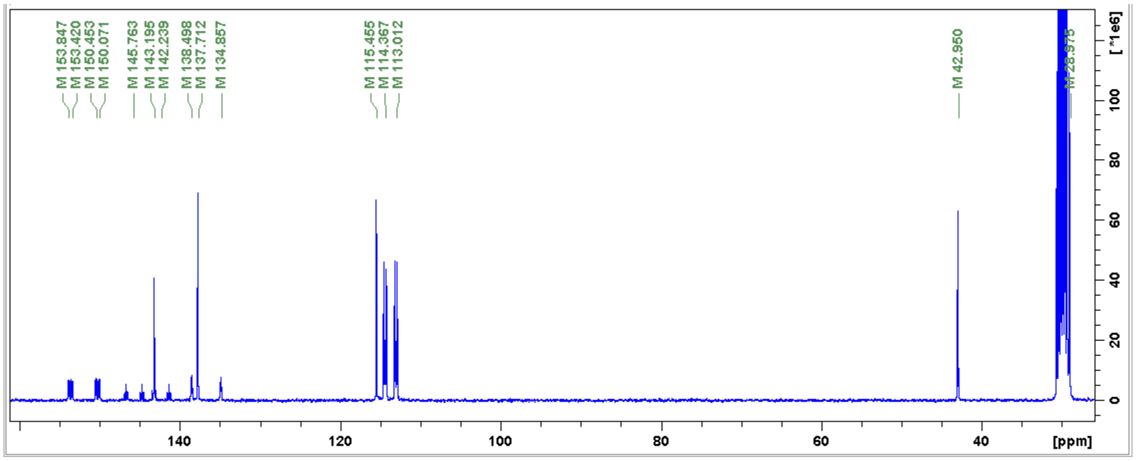

Compound IIh

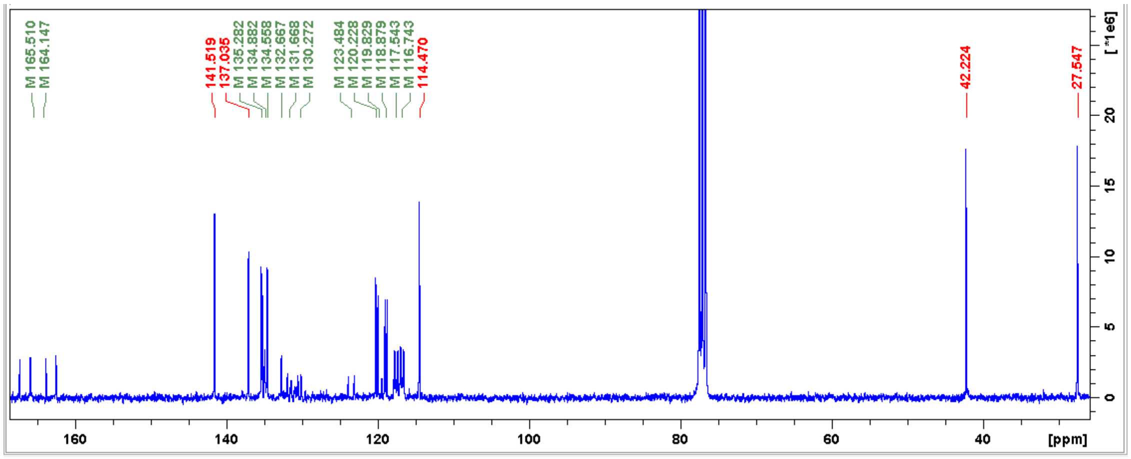

Compound IIi

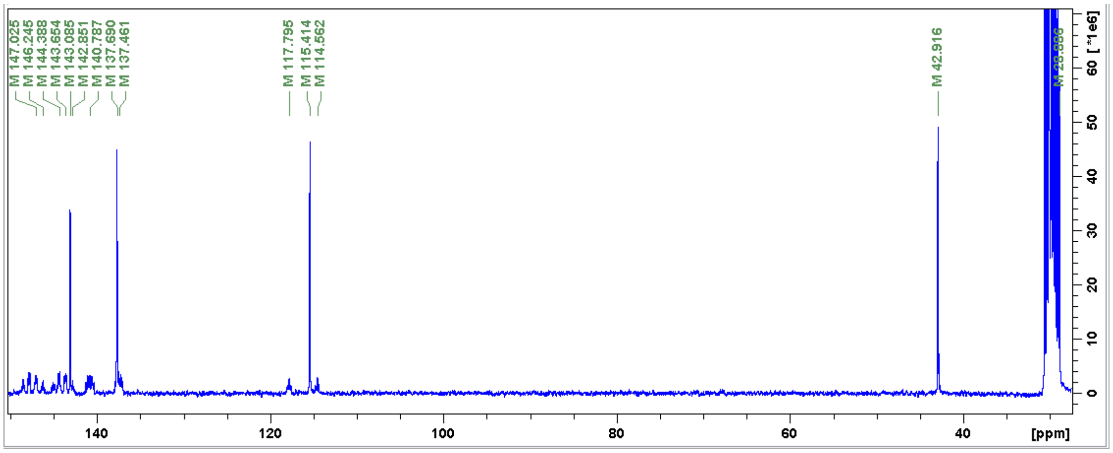

Compound IIj

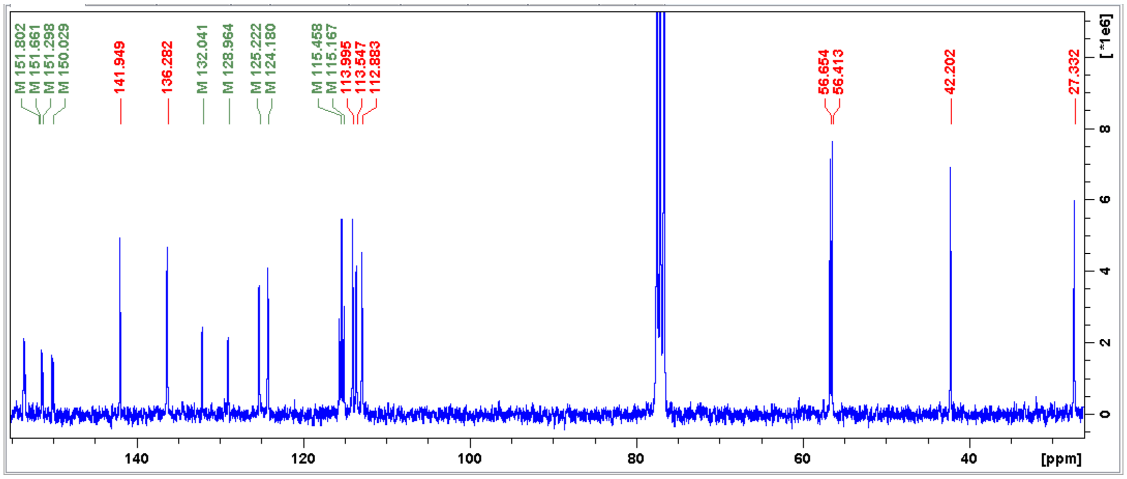

^{19}F NMR(300 MHz) of compounds Ic-Ij..S66

Compound Ic

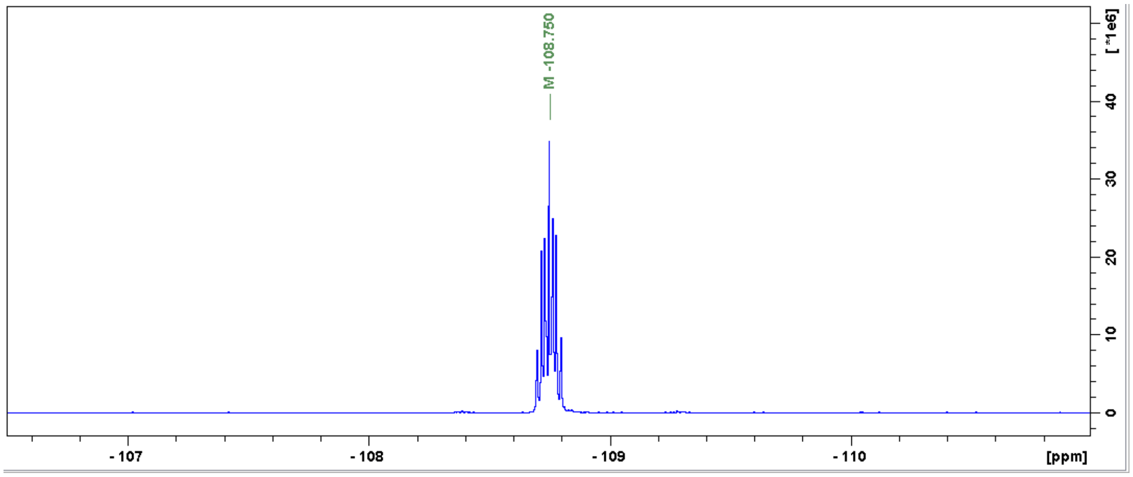

Compound Id

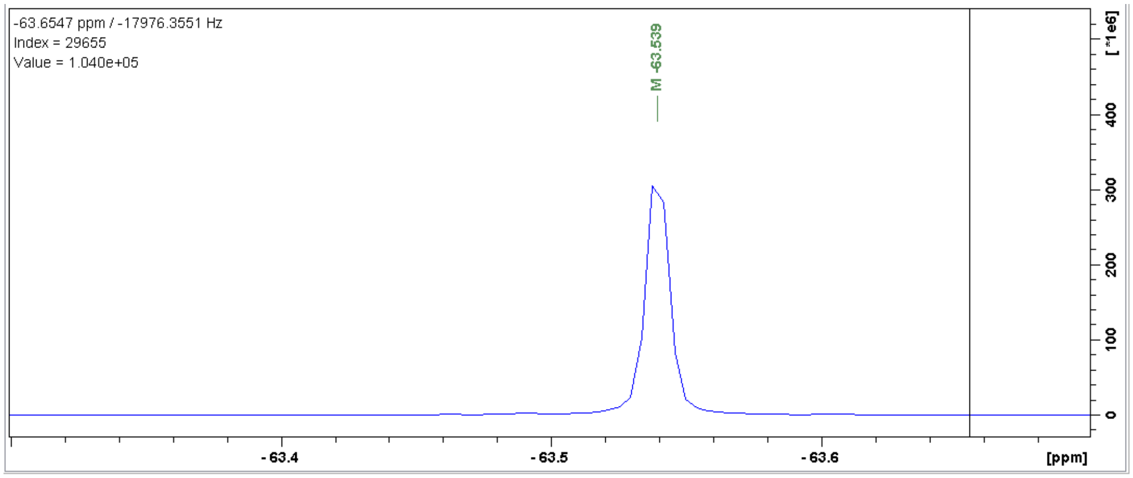

Compound Ie

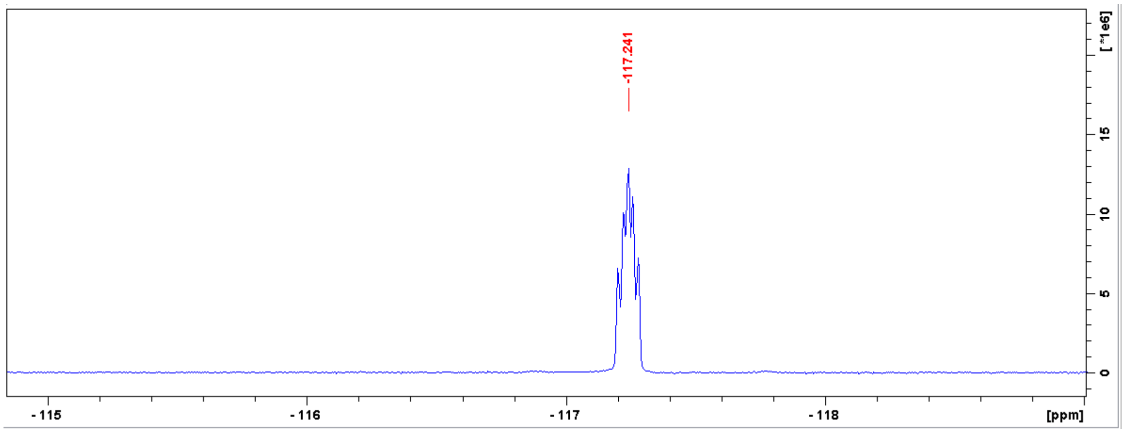

Compound If

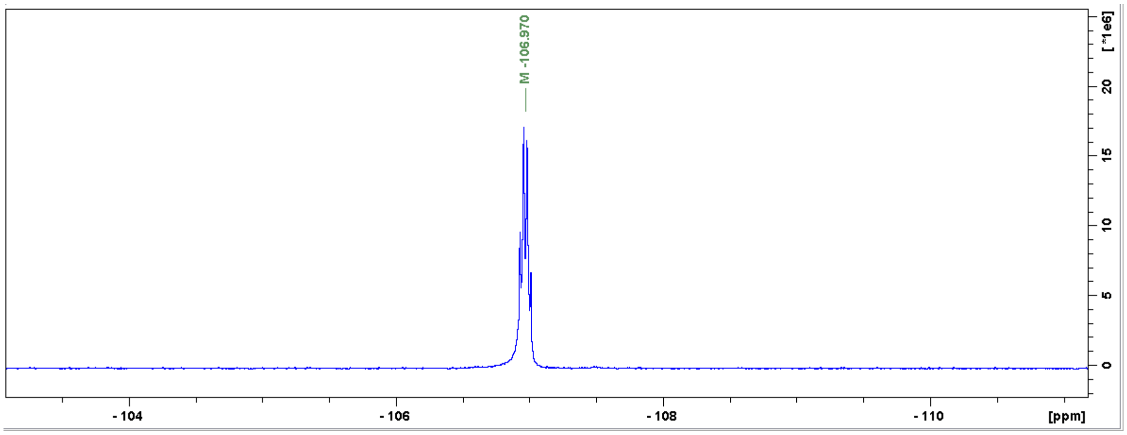

Compound Ig

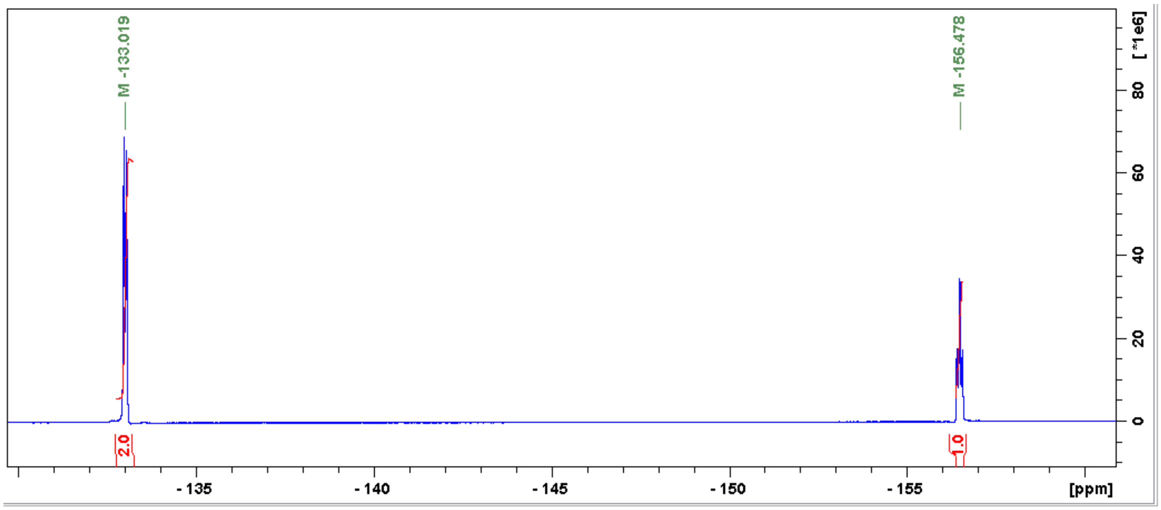

Compound Ih

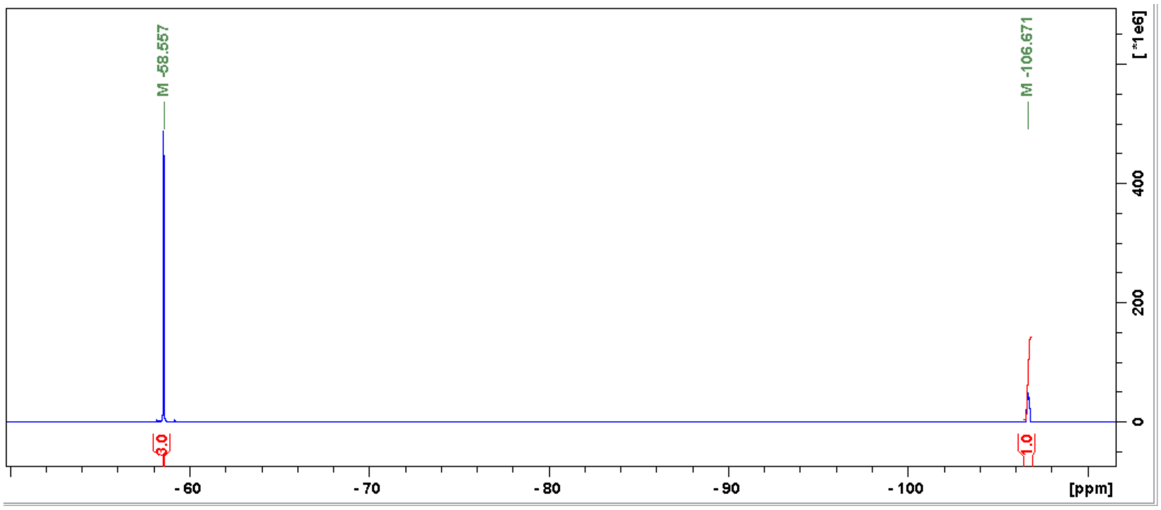

Compound Ii

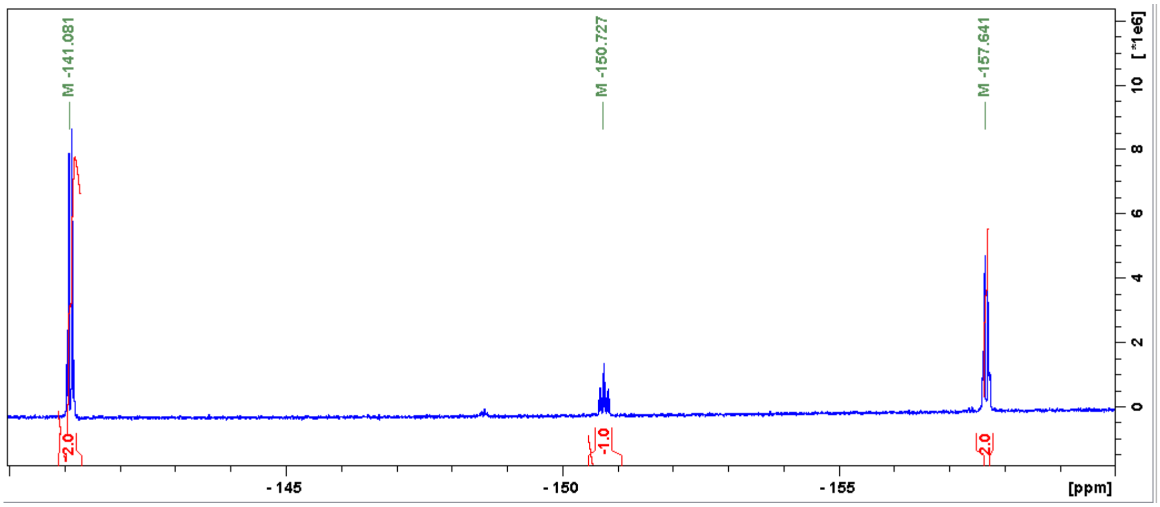

Compound Ij

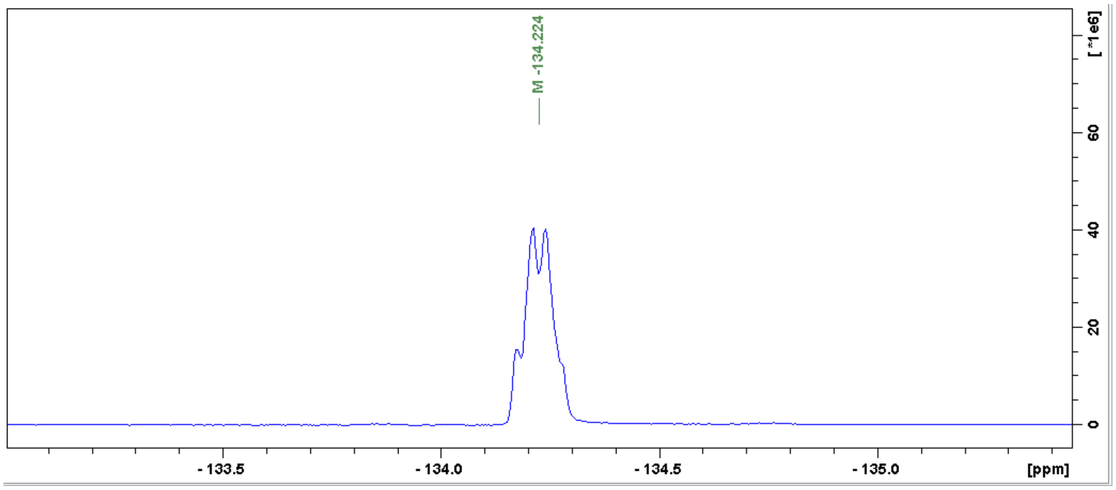

^{19}F NMR(300 MHz) of compounds IIc-IIj

Compound IIc

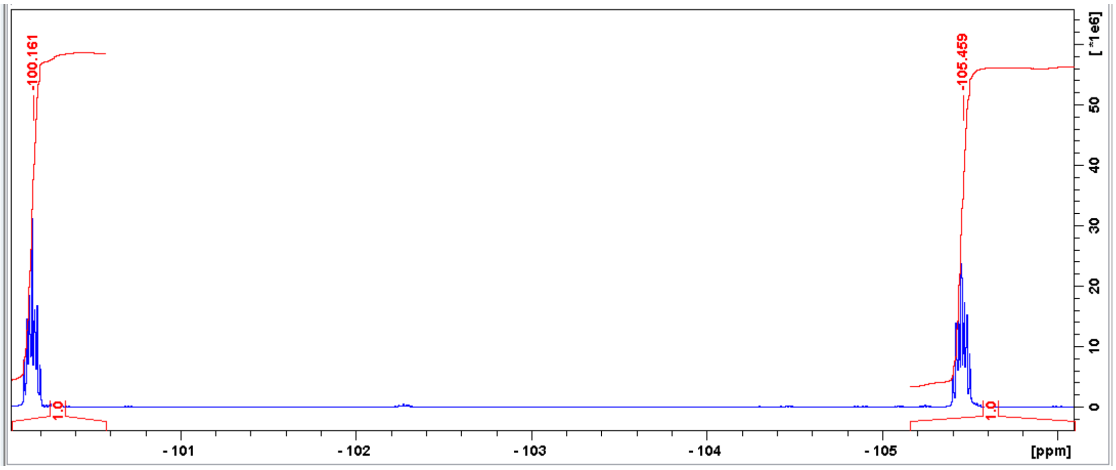

Compound IId

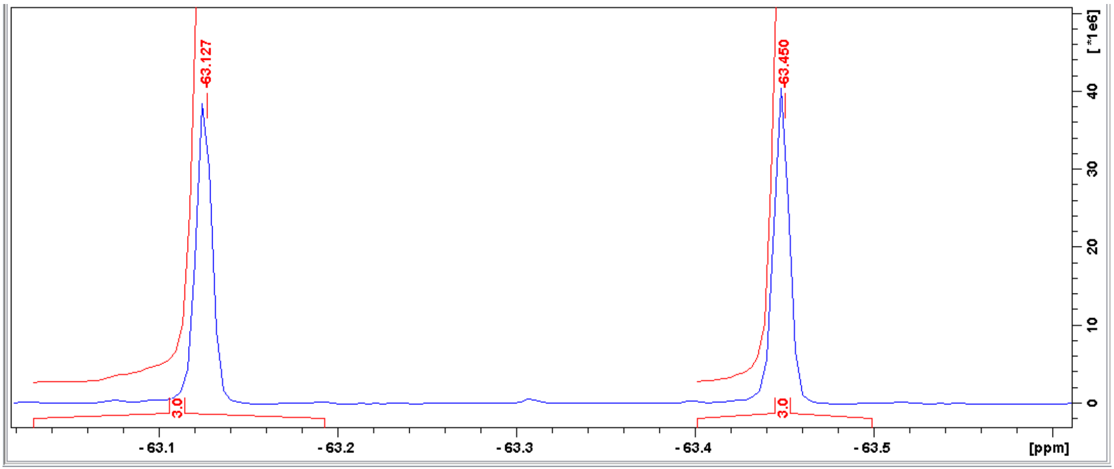

Compound IIe

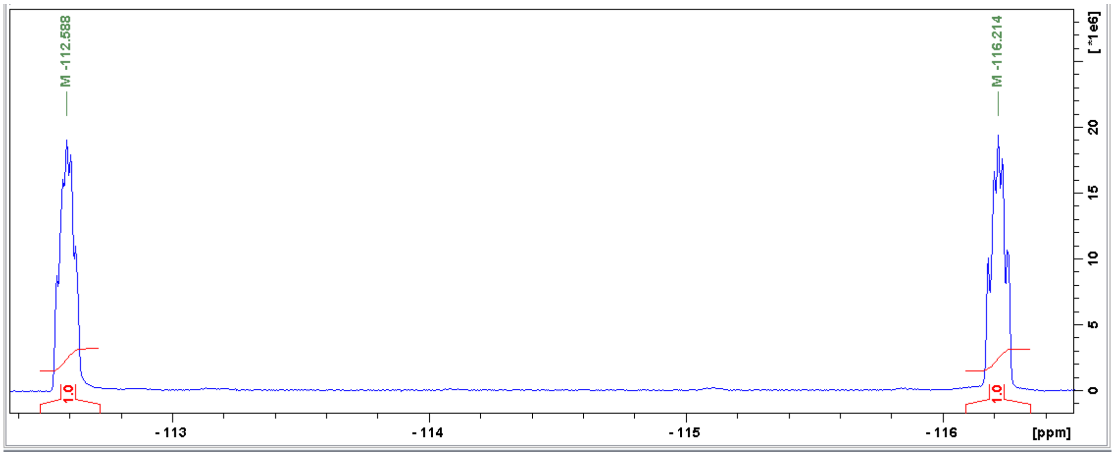

Compound IIf

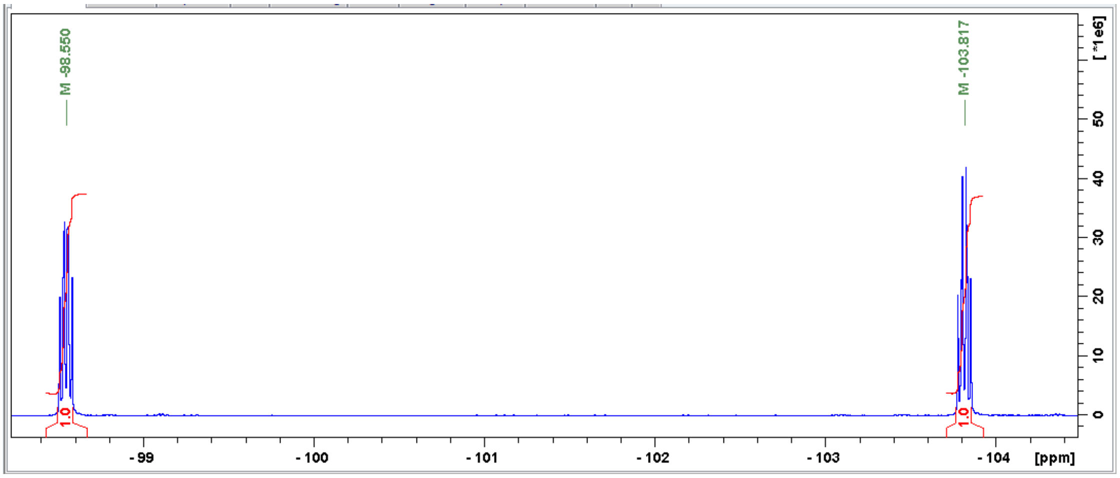

Compound IIg

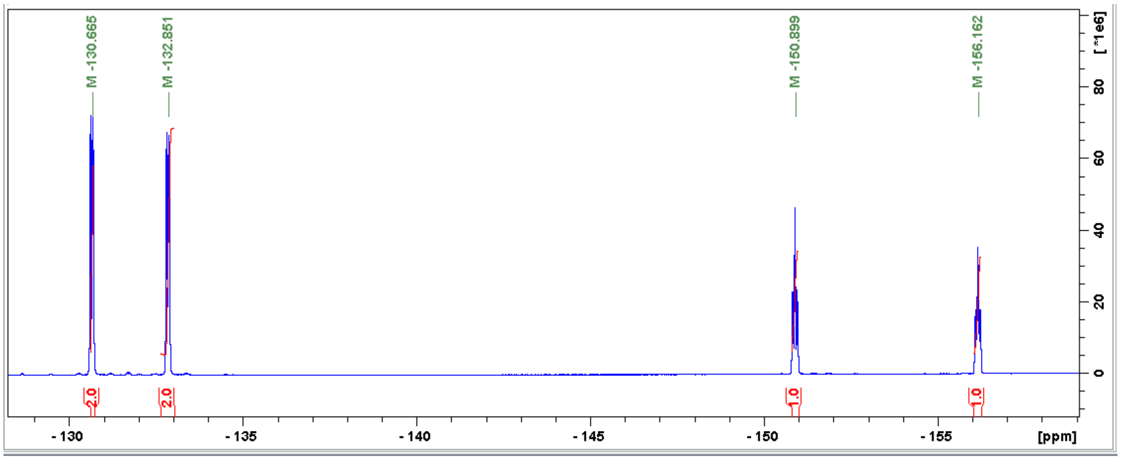

Compound IIh

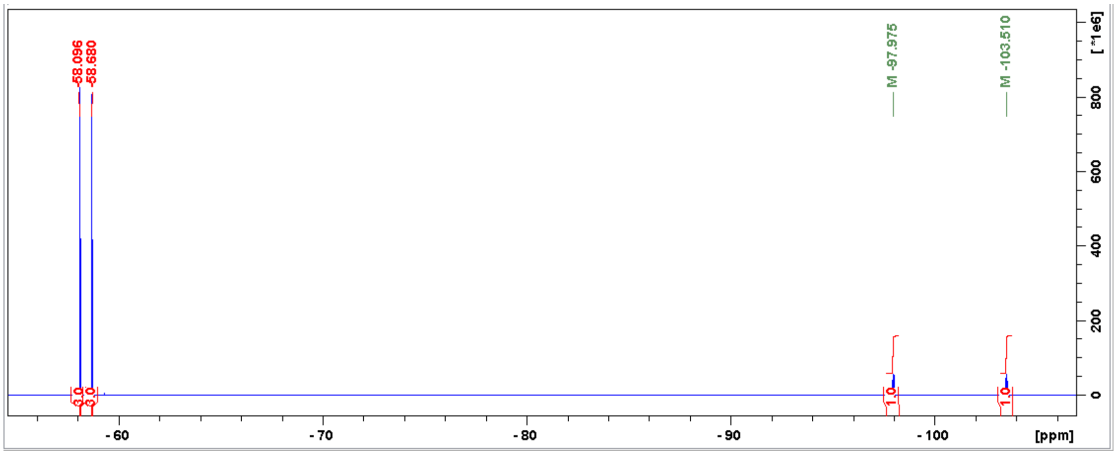

Compound IIi

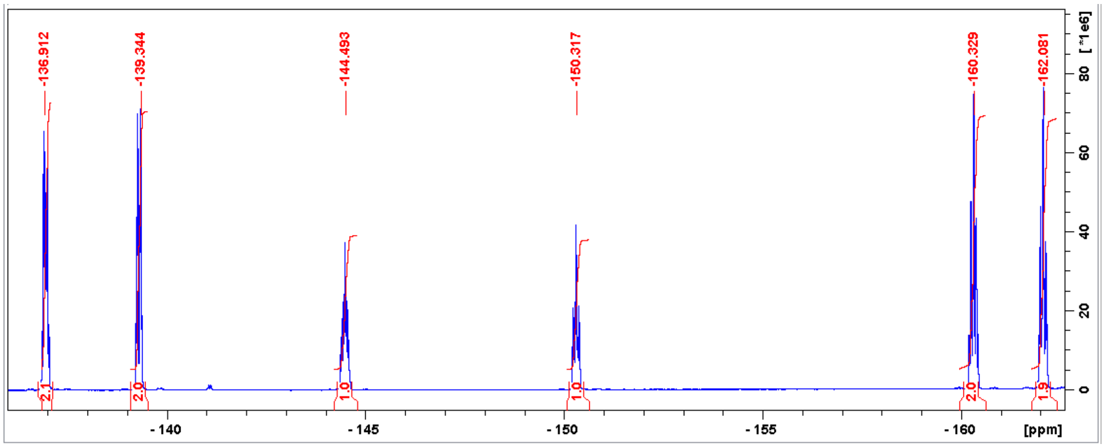

Compound IIj

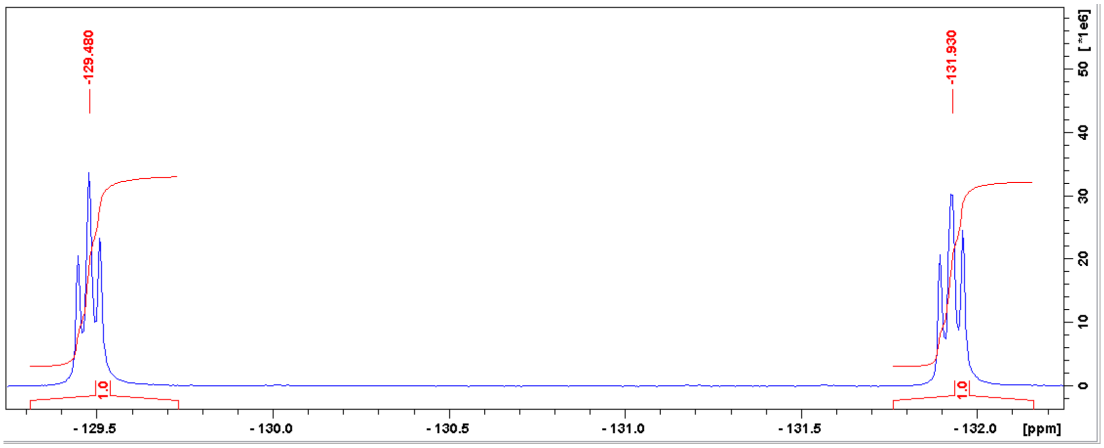

I-a

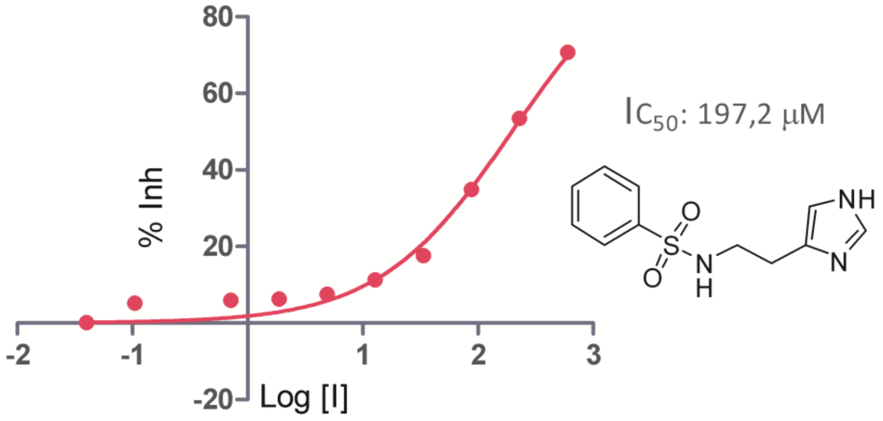

80
60
40
20
-20
% Inh
-2
-1
1
2
3
Log [I]
IC50: 197,2 μM
O
S
O
N
H
NH
N

I-b

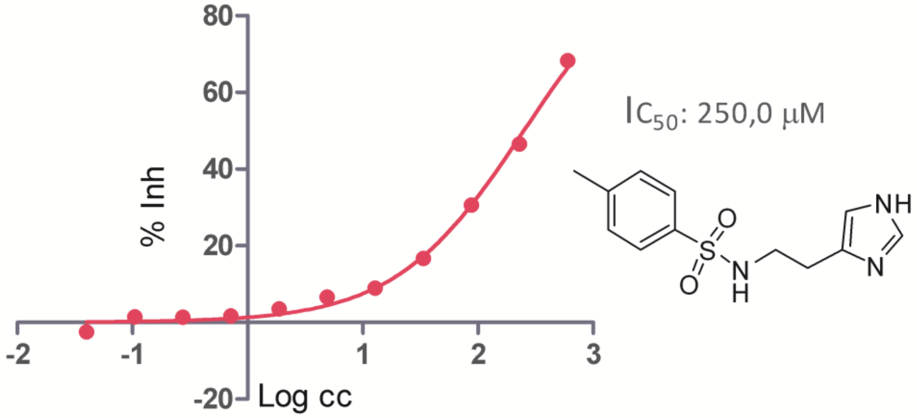

80
60
40
20
-20
% Inh
-2
-1
1
2
3
Log cc
IC50: 250,0 μM
O
S
O
N
H
NH
N

I-c

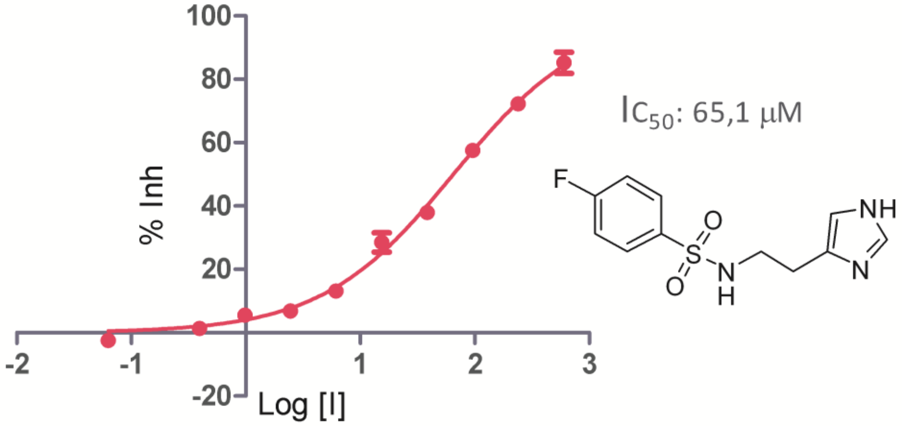

100
80
60
40
20
-20
% Inh
-2
-1
1
2
3
Log [I]
IC50: 65,1 μM
F
O
S
O
N
H
NH
N

I-d

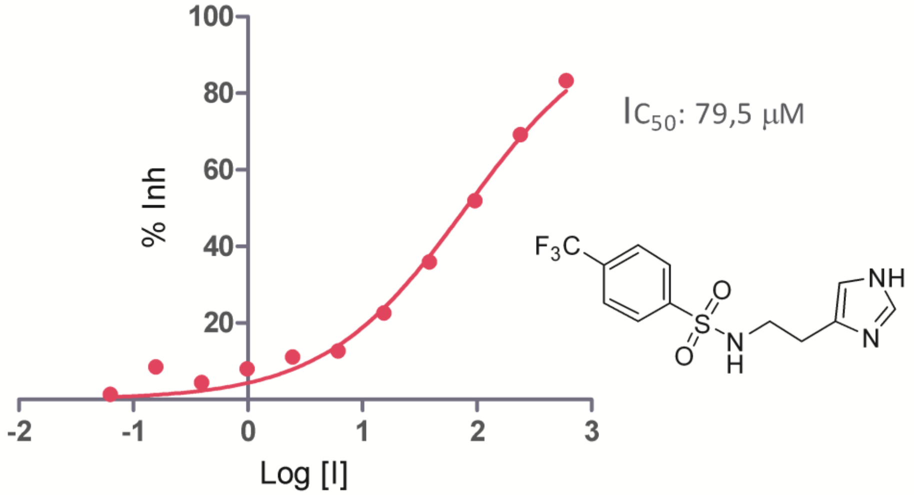
100
80
60
40
20
% Inh
-2
-1
0
1
2
3
Log [I]
IC50: 79,5 µM
F3C
O
S
O
N
H
NH
N

I-e

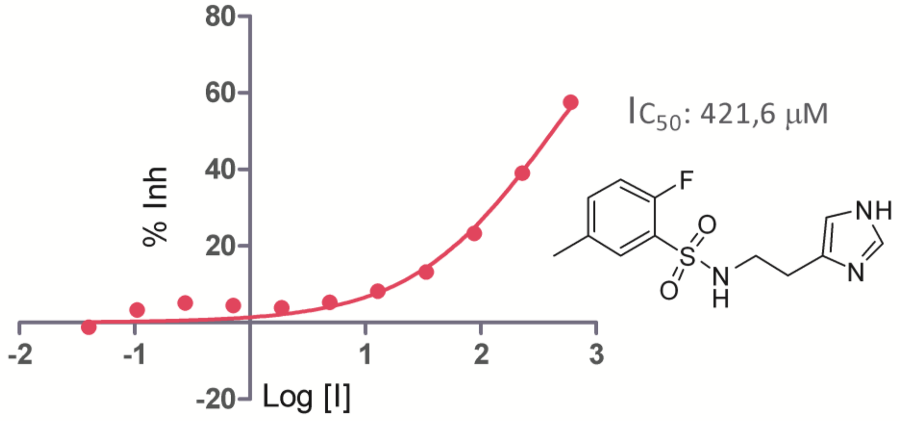
80
60
40
20
-20
% Inh
-2
-1
1
2
3
Log [I]
IC50: 421,6 µM
F
O
S
O
N
H
NH
N

I-f

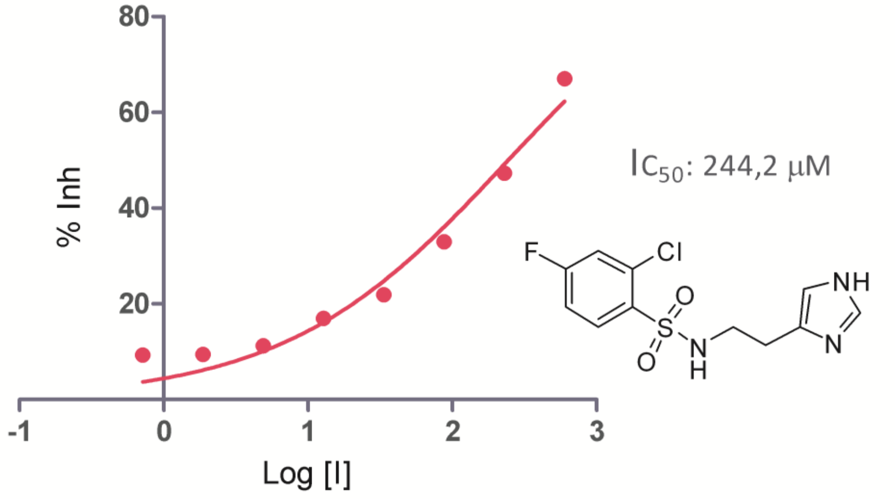

80
60
40
20
% Inh
-1
0
1
2
3
Log [I]
IC50: 244,2 μM
F
Cl
O
S
O
N
H
NH
N

I-g

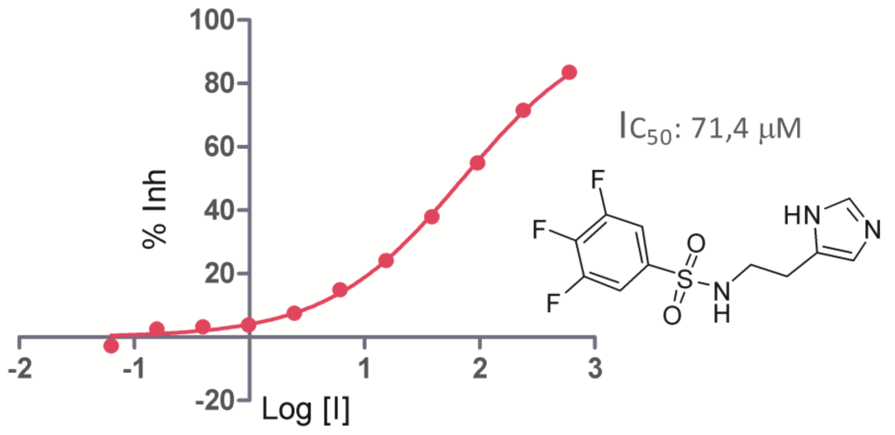

100
80
60
40
20
-20
% Inh
-2
-1
1
2
3
Log [I]
IC50: 71,4 μM
F
F
F
O
S
O
N
H
HN
N

I-h

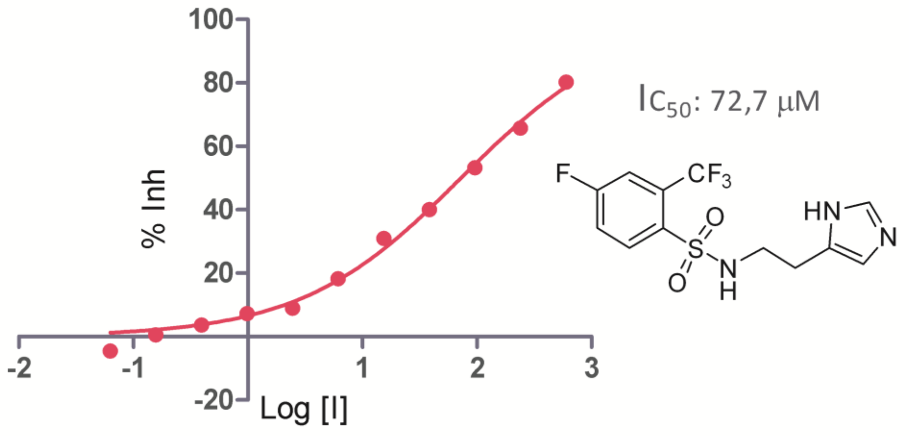

100
80
60
40
20
-20
% Inh
-2
-1
1
2
3
Log [I]
IC50: 72,7 μM
F
CF3
O
S
O
N
H
HN
N

I-i

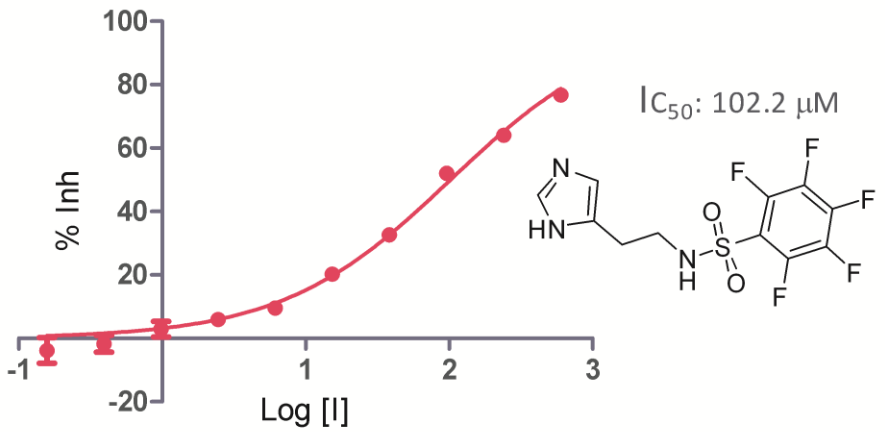
100
80
60
40
20
-20
% Inh
-1
1
2
3
Log [I]
IC50: 102.2 µM
N
HN
F
F
F
F
F
O
S
O
N
H

I-j

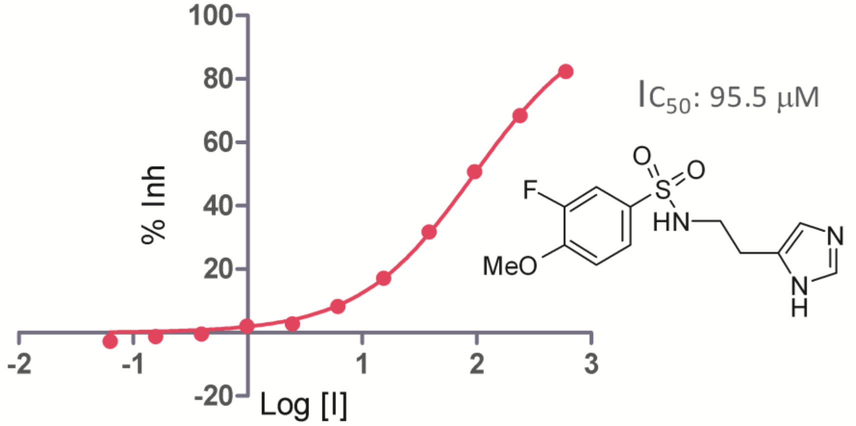
100
80
60
40
20
-20
% Inh
-2
-1
1
2
3
Log [I]
IC50: 95.5 µM
O
S
O
F
HN
MeO
N
N
H
